# Adherence and persistence to oral anticoagulants in patients with atrial fibrillation: A Belgian nationwide cohort study

**DOI:** 10.3389/fcvm.2022.994085

**Published:** 2022-09-29

**Authors:** Maxim Grymonprez, Andreas Capiau, Stephane Steurbaut, Els Mehuys, Koen Boussery, Tine L. De Backer, Lies Lahousse

**Affiliations:** ^1^Department of Bioanalysis, Pharmaceutical Care Unit, Faculty of Pharmaceutical Sciences, Ghent University, Ghent, Belgium; ^2^Center for Pharmaceutical Research, Research Group of Clinical Pharmacology and Clinical Pharmacy, Vrije Universiteit Brussel, Jette, Belgium; ^3^Department of Hospital Pharmacy, Universitair Ziekenhuis Brussel, Jette, Belgium; ^4^Department of Cardiology, Ghent University Hospital, Ghent, Belgium; ^5^Department of Epidemiology, Erasmus Medical Center, Rotterdam, Netherlands

**Keywords:** atrial fibrillation, oral anticoagulants, NOAC, VKA, persistence, adherence, reinitiation, switching

## Abstract

**Background:**

Since non-vitamin K antagonist oral anticoagulants (NOACs) do not require coagulation monitoring, concerns of lower adherence and persistence to NOACs than vitamin K antagonists (VKAs) have been raised. Moreover, little is known on the frequency of permanent cessation and switching between anticoagulants in patients with atrial fibrillation (AF). Therefore, persistence, reinitiation, switching and adherence to oral anticoagulants (OACs) were investigated.

**Materials and methods:**

AF patients with a first OAC prescription claim between 2013 and 2019 were identified in Belgian nationwide data. Persistence, reinitiation and switching were estimated using Kaplan-Meier analyses. Adherence was investigated using the proportion of days covered (PDC). Predictors for non-adherence and non-persistence were identified by multivariable logistic regression.

**Results:**

Among 277,782 AF patients, 69.6% NOAC and 37.2% VKA users were persistent after 1 year, whereas 44.3% and 18.9% after 5 years, respectively. After one year, 67.1% rivaroxaban, 68.1% dabigatran, 69.8% apixaban, and 76.9% edoxaban users were persistent. Among subjects having discontinued NOAC or VKA treatment, 75.4% and 46.1% reinitiated any OAC within 5 years, respectively. VKAs were more frequently switched to NOACs than vice versa (17.6% versus 2.5% after 1 year). After 1 year, a high PDC (≥ 90%) was observed in 87.8% apixaban, 88.6% dabigatran, 91.3% rivaroxaban, and 94.7% edoxaban users (90.2% NOAC users). Adherence and persistence were higher in older, female subjects, while lower in subjects with dementia or hyperpolypharmacy.

**Conclusion:**

Adherence and persistence to NOACs were high. However, 10% of subjects were non-adherent after 1 year and one-fourth did not reinitiate anticoagulation within 5 years after NOAC discontinuation.

## Introduction

Oral anticoagulants (OAC) are crucial for thromboembolic prevention in patients with atrial fibrillation (AF) (1). For decades, vitamin K antagonists (VKAs) were first-choice, but their use was limited by a slow onset of action, narrow therapeutic window requiring regular coagulation monitoring, and multiple drug and food interactions (1). Since 2010, non-vitamin K antagonist oral anticoagulants (NOACs) have emerged as effective and safe alternatives to VKAs (1). Following their approval, worldwide anticoagulant use has almost doubled in the last decade, with NOACs being more initiated than VKAs in newly-diagnosed AF patients since 2014 (2). In Belgium, NOAC uptake was remarkably fast, given the early experience with NOACs thanks to “compassionate use” programs, which allow the use of drugs with an approved European indication before they are commercially available (3, 4). In recent years, apixaban has become the most prescribed NOAC in Belgium (5).

However, NOACs are administered in fixed dosing regimens without the need for coagulation monitoring, which may result in less medical follow-up (1, 6). Moreover, NOACs have a shorter half-life and lower drug forgiveness than VKAs (6). Consequently, concerns of lower adherence (i.e., taking medication as prescribed) and persistence (i.e., continuation of treatment) to NOACs have been raised, especially since treatment non-adherence and discontinuation have been associated with higher thromboembolic and mortality risks (7–12). Although prior studies have shown that persistence to NOACs may be better than VKAs (13–16), they were often limited by a short follow-up and by inclusion of subjects with prior VKA use or subjects at low thromboembolic risk (CHA_2_DS_2_-VASc score 0 in men, 1 in women). Likewise, differences between the four NOACs are less established, especially since, to the best of our knowledge, no nationwide cohort study has investigated adherence and persistence in edoxaban users. Furthermore, anticoagulant reinitiation after discontinuation should also be investigated to distinguish between temporary treatment gaps and permanent treatment cessation, but this is less well-established on a full-population scale. Moreover, switching between anticoagulants is expected in clinical practice, but the rate and direction of switching are unclear, especially since some studies considered switching as discontinuation of treatment (17, 18).

Therefore, we aimed to investigate the persistence, reinitiation and switching of NOACs and VKAs in AF patients, and adherence to NOACs in persistent users on a full-population scale during long-term follow-up. In addition, factors associated with non-adherence and non-persistence were explored.

## Materials and methods

### Data sources

Two nationwide databases provided the source population, namely the InterMutualistic Agency (IMA) database and Minimal Hospital Dataset (MHD). The IMA centralizes all claims data from Belgian health insurance funds on reimbursed ambulatory and hospital care, including demographic characteristics (e.g., age, sex), medical procedures (diagnostic or therapeutic procedures and other reimbursed care) and drug prescription claims (19, 20). Since health insurance is legally mandatory in Belgium, the source population represents all legal residents with reimbursed medication or care. The MHD is managed by the Belgian Ministry of Health and aggregates hospital discharge diagnoses of every hospital admission (including hospitalizations, day-care stays, and emergency room contacts), coded in International Classification of Diseases (ICD) codes (ICD-9 up to 2014, ICD-10 from 2015 onward) (19, 21). Both databases were deterministically linked by the Trusted Third Party “eHealth” using the national social security number as unique patient identifier. Only pseudonymized data were available to the researchers on the secured IMA servers after applying an encrypting procedure for privacy protection. This study was approved by the IMA and MHD database administrators and by the “Sectoral Committee of Social Security and Health, Section Health,” a subcommittee of the Belgian Commission for the Protection of Privacy (approval code IVC/KSZG/20/344), waiving the need for individual informed consents (22). This study followed the Strengthening the Reporting of Observational Studies in Epidemiology (STROBE) reporting guideline (Supplementary Table 1; 23).

### Study population

Subjects ≥ 45 years old with ≥ 1 year coverage by a Belgian health insurance fund were included from the IMA database on the first date of filling an OAC prescription (= index date) during the study period from January 1st, 2013 to December 31st, 2019 (Supplementary Figure 1). NOAC (dabigatran, rivaroxaban, apixaban, and edoxaban) and VKA users (warfarin, acenocoumarol, and phenprocoumon) were included. Only OAC-naïve AF patients indicated for chronic anticoagulation were considered, excluding OAC-experienced subjects with an OAC prescription filled ≤ 1 year before the index date and subjects at low thromboembolic risk (CHA_2_DS_2_-VASc score 0 in men, 1 in women) on the index date (7, 12, 18, 24).

To avoid competing treatment indications for OACs, subjects were excluded in case of total hip or knee replacement surgery, or diagnosis of deep vein thrombosis or pulmonary embolism ≤ 6 months before the index date, based on specific ICD and/or medical procedure codes (Supplementary Table 2). Moreover, only AF patients eligible for NOACs and VKAs were examined, excluding subjects with valvular AF (mechanical prosthetic heart valve or moderate/severe mitral stenosis) and/or end-stage renal disease [chronic kidney disease (CKD) stage V and/or dialysis]. Lastly, subjects with ≥ 2 prescription claims of different types or doses of OACs on the index date, or subjects treated with NOAC doses not approved for stroke prevention in AF (e.g., rivaroxaban 10 mg) were excluded. Follow-up ended in case of death, emigration or end of the study period (December 31st, 2019).

### Outcomes

Persistence was defined as the time from OAC initiation (= index date) to discontinuation. Discontinuation was defined using a > 60-day supply gap after the calculated last day of supply, with the possibility to extend this gap for VKAs in case of intervening INR testing at least every 42 days (16, 25–27). This approach has shown to substantially reduce misclassification of VKA discontinuation (16, 26) and accounts for delays in refilling, minor non-adherence, variable VKA dosing regimens and residual drug effects (14–16, 27, 28). The date of discontinuation was defined as the calculated last day of supply (or last INR test date if VKA-treated, whichever came last) (16, 25–27). INR test dates were identified using medical procedure codes (Supplementary Table 2). The last day of supply was calculated based on ambulatory and hospital prescription claims data [dispensing date, Anatomical Therapeutic Chemical (ATC) classification code, tablet strength, package size, and number of packages supplied] and the recommended dosing regimen [twice-daily for dabigatran and apixaban; once-daily for rivaroxaban, and edoxaban; defined daily dose (DDD) of 7.5 mg for warfarin, 5 mg for acenocoumarol, and 3 mg for phenprocoumon (29)]. In case of overlap between two consecutive refills of the same OAC, the start date of the new dispensing was shifted to the day after the last day of supply of the prior dispensing (30). Stockpiling was allowed up to 180 days of accumulated days of supply (31).

Among subjects having discontinued treatment, the time from discontinuation to reinitiation of anticoagulation was investigated. The date of reinitiation was defined as the first OAC dispensing date after discontinuation.

Switching was defined as the dispensing of another OAC before or within 60 days after the last day of supply of the index OAC, considering the dispensing date of the new OAC as the switch date. The direction of switching (VKAs to NOACs or vice versa; specific NOAC type to any other OAC; and standard to reduced dose NOACs or vice versa) was assessed.

Lastly, adherence was investigated using the proportion of days covered (PDC) in patients persistent on NOAC treatment after a specific time interval (e.g., 1 year) (to avoid confusing non-adherence with non-persistence), in line with the taxonomy of the European ABC (Ascertaining Barriers to Compliance) Group (32, 33) and prior research (8, 34). Due to variable dosing regimens and lack of INR values, adherence to VKAs was not assessed. The PDC was calculated as the number of days covered with NOAC supply during a time interval, divided by the total number of days of that time interval (PDC range 0–100%). In order to specifically investigate patient-related NOAC adherence at home, only ambulatory prescription claims were considered, consistent with previous studies (7, 10, 12). In case of hospitalization, the length of inpatient stay was deducted from the number of days in the studied time interval (7, 10, 12). A PDC of < 90% was defined as non-adherence, as higher thromboembolic and mortality risks have been observed in OAC-treated patients with an adherence below 90% (7, 8, 34).

### Covariates

Age, sex, comorbidities, medication history, and prescriber type were assessed at baseline. Comorbidities were identified using specific ICD-coded diagnoses (e.g., cancer), medical procedure codes (e.g., cancer-related surgery) and/or ATC-coded prescription claims (e.g., antineoplastic drugs) ≤ 1 year before the index date (Supplementary Table 2). Comorbidities included hypertension, heart failure, coronary, and peripheral artery disease, valvular heart disease (aortic, mitral, pulmonary or tricuspid valve disease, valve repair or bioprosthetic heart valve replacement), dyslipidemia, CKD (stage III-IV or renal transplant), chronic liver disease (including liver cirrhosis or liver transplant), chronic lung disease (including COPD, asthma, or interstitial lung disease), obstructive sleep apnea, cancer, upper and lower gastrointestinal tract disorders (gastroesophageal reflux disease or peptic ulcer disease; and diverticulosis, angiodysplasia, colorectal polyposis or hemorrhoids, respectively), diabetes mellitus, thyroid disease, anemia, osteoporosis, dementia, Parkinson’s disease, history of falling, frailty [using the Claims-based Frailty Indicator (35)], prior thromboembolism (stroke or systemic embolism) and prior major or clinically relevant non-major bleeding (intracranial, gastrointestinal, urogenital or other bleeding event necessitating hospitalization).

Medication history was identified with ATC-coded prescription claims, considering recent use ≤ 6 months before the index date. Based on the total number of previously dispensed drugs, baseline polypharmacy and hyperpolypharmacy were defined as the dispensing of 5–9 and ≥ 10 concomitant drugs during ≥ 30 days (consecutive or not) in the last 6 months before the index date, respectively (36).

Prescriber type of the physician initiating the OAC was assessed using the last three digits of the physician’s identification code of the Belgian National Institute for Health and Disability Insurance, and categorized as primary care physician, cardiologist or other secondary care physician.

Lastly, the CHA_2_DS_2_-VASc score, HAS-BLED score and age-adjusted Charlson Comorbidity Index were calculated (37). Since only INR test dates but not values were available, a modified HAS-BLED score was used without the “labile INR” criterion. Alcohol abuse was indirectly identified with ICD (e.g., alcoholic liver disease), ATC (e.g., disulfiram) or medical procedure codes (e.g., visit to psychologist for alcohol abuse).

### Statistical analyses

Mean ± standard deviation, and counts and percentages were presented for continuous and categorical variables, respectively. Kaplan-Meier time-to-event analyses were used to estimate persistence, reinitiation and switching over time. The log-rank test was used to compare NOACs to VKAs. To assess persistence, patients were censored in case of switching before discontinuation or end of follow-up, whichever occurred first (7). When examining reinitiation rates, subjects were censored at end of follow-up. To investigate switching rates, subjects were censored in case of discontinuation before switching or end of follow-up, whichever occurred first. To examine adherence in persistent NOAC users, the PDC was calculated for several time intervals (3 months to 7 years) in the subgroup of subjects with a follow-up duration at least corresponding with the examined time interval (e.g., PDC after 1 year in the subgroup of subjects with ≥ 1 year of follow-up). The proportion of subjects with a PDC ≥ 90% was illustrated over time, stratified by NOAC type.

Predictors for non-persistence (versus persistence) and non-adherence (PDC < 90% versus ≥ 90%) among incident NOAC users after 1 year were explored using multivariable logistic regression in the subgroup of subjects with ≥ 1 year of follow-up. NOAC type (with dabigatran, the first approved NOAC, as reference), age (≥ 75 versus < 75 years), sex, cardiovascular comorbidities, geriatric traits (e.g., frailty), thromboembolism-and/or bleeding-related comorbidities (e.g., cancer), (hyper)polypharmacy, prescriber type and time period (dichotomized as July 2016-December 2019 versus January 2013-June 2016) were fitted in a multivariable model with backward elimination. Only statistically significant factors using a two-sided *p*-value of < 0.05 were retained in the final model. Odds ratios (OR) with 95% confidence intervals (CIs) were calculated and visualized in forest plots. Additionally, adjusted ORs for non-persistence and non-adherence when comparing other NOAC types were also calculated and visualized after adjusting for the same factors of the final model. Lastly, due to the transition from ICD-9 to ICD-10 in 2015, and incomplete data transfer in 2019, some ICD-coded hospital discharge diagnoses were missing at the time of data delivery (7.9% of the cohort). These missing data were accounted for through multiple imputations by chained equations. All analyses were performed in R (R version 3.6.0).

### Sensitivity analyses

Several sensitivity analyses were performed to check the robustness of results. First, persistence was assessed using a > 30-day and > 90-day supply gap. Second, adherence to NOACs was investigated in persistent and non-persistent NOAC users (censoring subjects only in case of death, emigration or end of study period). Third, ambulatory and hospital prescription claims were considered for the PDC calculation. Fourth, only subjects at high thromboembolic risk (CHA_2_DS_2_-VASc score ≥ 2 in men, ≥ 3 in women) were investigated. Fifth, the study population was restricted to subjects having initiated treatment between October 1st, 2016 and December 31st, 2019, when all NOACs were commercially available in Belgium, to avoid time-period bias and account for the shorter follow-up of edoxaban compared to other NOACs. Lastly, analyses were repeated in the subgroup of subjects with ≥ 1 year of follow-up.

## Results

### Baseline characteristics

**FIGURE 1:**
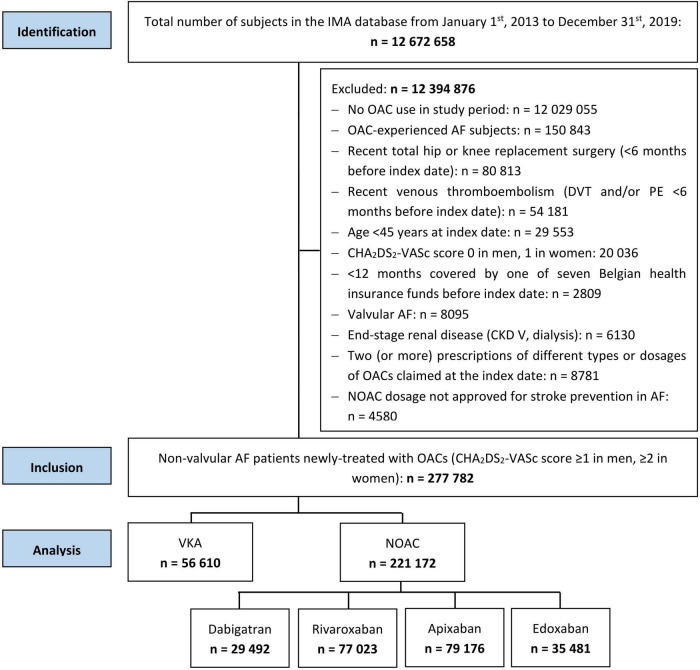
Strengthening the reporting of observational studies in epidemiology (STROBE) diagram of study population. AF, atrial fibrillation; CKD, chronic kidney disease; DVT, deep vein thrombosis; IMA, InterMutualistic Agency; NOAC, non-vitamin K antagonist oral anticoagulant; OAC, oral anticoagulant; PE, pulmonary embolism; STROBE, Strengthening the Reporting of Observational Studies in Epidemiology; VKA, vitamin K antagonist.

With a mean follow-up of 2.9 ± 2.0 years, 277,782 newly-treated AF patients at intermediate-to-high thromboembolic risk were identified (Figure 1). The 221,172 NOAC and 56,610 VKA users were on average 77.2 ± 9.4 and 73.1 ± 11.3 years old, and 48.1 and 46.9% were female, respectively. Baseline characteristics are summarized in Table 1.

**TABLE 1:** Baseline characteristics of oral anticoagulant (OAC)-naïve non-valvular atrial fibrillation (AF) subjects with a CHA_2_DS_2_-VASc score of ≥ 1 in men or ≥ 2 in women.

Patient characteristics	VKA (*n* = 56,610)	NOAC				
		Overall (*n* = 221,172)	Dabigatran (*n* = 29,492)	Rivaroxaban (*n* = 77,023)	Apixaban (*n* = 79,176)	Edoxaban (*n* = 35,481)
Age (years)	73.1 ± 11.3	77.2 ± 9.4	76.7 ± 9.2	76.7 ± 9.4	78.0 ± 9.3	77.1 ± 9.6
< 65 years	14,043 (24.8%)	19,569 (8.8%)	2804 (9.5%)	7280 (9.5%)	6142 (7.8%)	3343 (9.4%)
65–74 years	16,157 (28.5%)	66,950 (30.3%)	9168 (31.1%)	24,101 (31.3%)	22,444 (28.3%)	11,237 (31.7%)
75–84 years	17,645 (31.2%)	85,594 (38.7%)	11,763 (39.9%)	30,127 (39.1%)	30,769 (38.9%)	12,935 (36.5%)
≥ 85 years	8765 (15.5%)	49,059 (22.2%)	5757 (19.5%)	15,515 (20.1%)	19,821 (25.0%)	7966 (22.5%)
Female	26,575 (46.9%)	106,404 (48.1%)	13,852 (47.0%)	36,652 (47.6%)	39,284 (49.6%)	16,616 (46.8%)
Reduced dose	NA	82,170 (37.2%)	16,463 (55.8%)	30,697 (39.9%)	23,882 (30.2%)	11,128 (31.4%)
Follow-up (years)	3.7 ± 2.1	2.7 ± 1.9	3.4 ± 2.1	3.3 ± 2.0	2.4 ± 1.7	1.3 ± 0.9
**Comorbidities**						
Hypertension	39,183 (69.2%)	152,277 (68.9%)	20,006 (67.8%)	52,305 (67.9%)	56,070 (70.8%)	23,895 (67.3%)
Coronary artery disease	15,181 (26.8%)	39,085 (17.7%)	4662 (15.8%)	13,261 (17.2%)	15,019 (19.0%)	6143 (17.3%)
Congestive heart failure	10,330 (18.2%)	34,535 (15.6%)	3809 (12.9%)	11,621 (15.1%)	13,934 (17.6%)	5170 (14.6%)
Valvular heart disease	12,579 (22.2%)	27,564 (12.5%)	3284 (11.1%)	8619 (11.2%)	10,993 (13.9%)	4668 (13.2%)
Peripheral artery disease	7419 (13.1%)	17,251 (7.8%)	2090 (7.1%)	5445 (7.1%)	7064 (8.9%)	2651 (7.5%)
Dyslipidemia	34,295 (60.6%)	127,879 (57.8%)	17,522 (59.4%)	43,226 (56.1%)	46,705 (59.0%)	20,426 (57.6%)
Chronic kidney disease	9097 (16.1%)	24,524 (11.1%)	2002 (6.8%)	7428 (9.6%)	10,912 (13.8%)	4183 (11.8%)
Chronic liver disease	2648 (4.7%)	7206 (3.3%)	816 (2.8%)	2482 (3.2%)	2673 (3.4%)	1234 (3.5%)
Chronic lung disease	8509 (15.0%)	26,967 (12.2%)	3201 (10.9%)	9443 (12.3%)	10,299 (13.0%)	4024 (11.3%)
Obstructive sleep apnea	2296 (4.1%)	8270 (3.7%)	994 (3.4%)	2788 (3.6%)	2992 (3.8%)	1496 (4.2%)
Cancer	5991 (10.6%)	23,939 (10.8%)	2729 (9.3%)	8053 (10.5%)	8805 (11.1%)	4352 (12.3%)
Upper GI tract disorder	5192 (9.2%)	16,139 (7.3%)	1862 (6.3%)	5727 (7.4%)	6339 (8.0%)	2211 (6.2%)
Lower GI tract disorder	4137 (7.3%)	16,054 (7.3%)	1928 (6.5%)	5483 (7.1%)	5949 (7.5%)	2694 (7.6%)
Diabetes mellitus	24,136 (42.6%)	72,548 (32.8%)	8685 (29.4%)	24,253 (31.5%)	28,011 (35.4%)	11,598 (32.7%)
Thyroid disease	8711 (15.4%)	32,698 (14.8%)	4137 (14.0%)	11,495 (14.9%)	12,367 (15.6%)	4699 (13.2%)
Anemia	6801 (12.0%)	17,486 (7.9%)	1777 (6.0%)	5796 (7.5%)	7149 (9.0%)	2764 (7.8%)
Osteoporosis	3951 (7.0%)	16,315 (7.4%)	2079 (7.0%)	5743 (7.5%)	6017 (7.6%)	2475 (7.0%)
Dementia	2929 (5.2%)	12,527 (5.7%)	1393 (4.7%)	4330 (5.6%)	5054 (6.4%)	1750 (4.9%)
Parkinson’s disease	1631 (2.9%)	7039 (3.2%)	918 (3.1%)	2326 (3.0%)	2738 (3.5%)	1057 (3.0%)
History of falling	4255 (7.5%)	19,083 (8.6%)	1969 (6.7%)	5788 (7.5%)	8141 (10.3%)	3185 (9.0%)
Frailty	14,480 (25.6%)	70,711 (32.0%)	8405 (28.5%)	22,734 (29.5%)	28,895 (36.5%)	10,677 (30.1%)
Prior thromboembolism	9357 (16.5%)	31,358 (14.2%)	5472 (18.6%)	8473 (11.0%)	13,822 (17.5%)	3591 (10.1%)
Prior MB/CRNMB	4253 (7.5%)	12,726 (5.8%)	1523 (5.2%)	4018 (5.2%)	5122 (6.5%)	2063 (5.8%)
**Medication history**						
Number of concomitant drugs	7.4 ± 4.6	6.8 ± 4.2	6.4 ± 3.8	6.8 ± 4.1	7.1 ± 4.4	6.7 ± 4.2
Polypharmacy (5–9)	25,276 (44.6%)	102,867 (46.5%)	14,133 (47.9%)	36,325 (47.2%)	36,287 (45.8%)	16,122 (45.4%)
Hyperpolypharmacy (≥ 10)	15,243 (26.9%)	47,915 (21.7%)	5178 (17.6%)	16,136 (20.9%)	19,313 (24.4%)	7288 (20.5%)
Rate control therapy	33,766 (59.6%)	147,739 (66.8%)	19,352 (65.6%)	50,533 (65.6%)	54,041 (68.3%)	23,813 (67.1%)
Beta blockers	32,062 (56.6%)	140,018 (63.3%)	18,322 (62.1%)	47,560 (61.7%)	51,349 (64.9%)	22,787 (64.2%)
Verapamil, diltiazem	2093 (3.7%)	8554 (3.9%)	1166 (4.0%)	3286 (4.3%)	2982 (3.8%)	1120 (3.2%)
Digoxin	3679 (6.5%)	21,587 (9.8%)	2686 (9.1%)	7241 (9.4%)	8264 (10.4%)	3396 (9.6%)
Rhythm control therapy	13,529 (23.9%)	72,097 (32.6%)	9859 (33.4%)	26,916 (34.9%)	24,979 (31.5%)	10,343 (29.2%)
Class I AAD	2926 (5.2%)	21,065 (9.5%)	3111 (10.5%)	8053 (10.5%)	6549 (8.3%)	3352 (9.4%)
Class III AAD	11,408 (20.2%)	56,148 (25.4%)	7506 (25.5%)	20,951 (27.2%)	20,033 (25.3%)	7658 (21.6%)
Antiplatelet	23,467 (41.5%)	98,502 (44.5%)	13,120 (44.5%)	33,686 (43.7%)	36,075 (45.6%)	15,621 (44.0%)
Acetylsalicylic acid	21,770 (38.5%)	91,410 (41.3%)	12,247 (41.5%)	31,470 (40.9%)	33,381 (42.2%)	14,312 (40.3%)
P2Y12 inhibitor	3714 (6.6%)	14,359 (6.5%)	1736 (5.9%)	4386 (5.7%)	5495 (6.9%)	2742 (7.7%)
ACE inhibitor/ARB	30,107 (53.2%)	116,668 (52.7%)	15,474 (52.5%)	40,726 (52.9%)	42,270 (53.4%)	18,198 (51.3%)
DHP calcium channel blocker	18,807 (33.2%)	70,374 (31.8%)	9019 (30.6%)	23,590 (30.6%)	26,577 (33.6%)	11,188 (31.5%)
Loop diuretic	20,413 (36.1%)	65,853 (29.8%)	7567 (25.7%)	22,861 (29.7%)	25,297 (32.0%)	10,128 (28.5%)
Non-loop diuretic	21,015 (37.1%)	83,508 (37.8%)	10,813 (36.7%)	29,127 (37.8%)	30,225 (38.2%)	13,343 (37.6%)
Proton pump inhibitor	25,494 (45.0%)	90,800 (41.1%)	11,208 (38.0%)	30,849 (40.1%)	33,965 (42.9%)	14,778 (41.7%)
NSAID	14,819 (26.2%)	51,687 (23.4%)	7043 (23.9%)	18,730 (24.3%)	17,779 (22.5%)	8135 (22.9%)
Oral corticosteroids	13,528 (23.9%)	45,237 (20.5%)	5438 (18.4%)	15,952 (20.7%)	16,438 (20.8%)	7409 (20.9%)
SSRI/SNRI	7713 (13.6%)	26,959 (12.2%)	3460 (11.7%)	9658 (12.5%)	10,121 (12.8%)	3720 (10.5%)
**Clinical risk score**						
CHA_2_DS_2_-VASc score	3.6 ± 1.8	3.7 ± 1.6	3.6 ± 1.6	3.5 ± 1.6	3.9 ± 1.7	3.5 ± 1.6
HAS-BLED score	2.6 ± 1.3	2.6 ± 1.2	2.6 ± 1.1	2.5 ± 1.1	2.7 ± 1.2	2.5 ± 1.2
Charlson comorbidity index	4.5 ± 2.4	4.6 ± 2.2	4.4 ± 2.1	4.4 ± 2.2	4.8 ± 2.3	4.5 ± 2.3
**Prescriber**						
Primary care physician	33,162 (58.6%)	80,888 (36.6%)	11,146 (37.8%)	32,035 (41.6%)	27,350 (34.5%)	10,358 (29.2%)
Cardiologist	10,601 (18.7%)	89,477 (40.5%)	12,218 (41.4%)	28,980 (37.6%)	30,998 (39.2%)	17,282 (48.7%)
Other physician	12,847 (22.7%)	50,806 (23.0%)	6128 (20.8%)	16,008 (20.8%)	20,828 (26.3%)	7842 (22.1%)

Data shown as mean ± standard deviation, or counts and percentages. Incident VKA users included 27,392 acenocoumarol, 15,412 warfarin and 13,806 phenprocoumon users. AAD, antiarrhythmic drug; ACE, angiotensin-converting enzyme; ARB, angiotensin II receptor blocker; CRNMB, clinically relevant non-major bleeding; DHP, dihydropyridine; GI, gastrointestinal; MB, major bleeding; NA, not applicable; NOAC, non-vitamin K antagonist oral anticoagulant; NSAID, non-steroidal anti-inflammatory drug; OAC, oral anticoagulant; SNRI, serotonin and norepinephrine reuptake inhibitor; SSRI, selective serotonin reuptake inhibitor; VKA, vitamin K antagonist.

### Persistence

**FIGURE 2:**
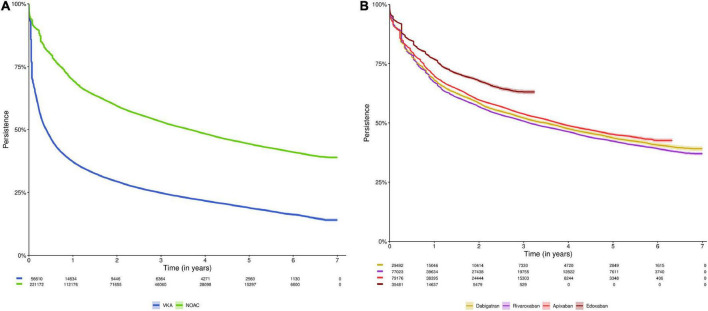
Kaplan-Meier analysis of persistence to **(A)** NOACs versus VKAs; and **(B)** NOAC types (dabigatran, rivaroxaban, apixaban, and edoxaban). Data shown as Kaplan-Meier estimates with 95% confidence interval and risk table (number of patients at risk). In analysis **(A)**, subjects were censored in case of switching from NOACs to VKAs or vice versa (allowing switching between NOAC types or between VKA types), death, emigration, or end of the study period. In analysis **(B)**, subjects were censored in case of switching to any other OAC type, death, emigration, or end of the study period. Due to their respective approval in September 2013 and October 2016, and the study period ending on December 31st, 2019, the maximum follow-up duration of apixaban and edoxaban users was limited to 6.3 years and 3.25 years, respectively. NOAC, non-vitamin K antagonist oral anticoagulant; OAC, oral anticoagulant; VKA, vitamin K antagonist.

Among NOAC and VKA users, 69.6% and 37.2% of subjects were persistent after 1 year, 59.4% and 29.4% after 2 years, and 44.3% and 18.9% after 5 years, respectively (Figure 2A, Supplementary Table 3). The median time until half of users discontinued treatment was 0.39 years [95%CI (0.38–0.40)] for VKA users, as compared to 3.65 years [95%CI (3.59–3.71)] in NOAC users (*p*-value log-rank test < 0.001). Among dabigatran, rivaroxaban, apixaban and edoxaban users, 68.1%, 67.1%, 69.8%, and 76.9% of subjects were persistent after 1 year; 58.3%, 56.8%, 59.7%, and 67.8% after 2 years; and 43.7%, 42.3%, and 45.2% after 5 years (not available for edoxaban due to limited follow-up), respectively (Figure 2B, Supplementary Table 3). After 3.49 years [95%CI (3.28–3.64)], 3.12 years [95%CI (3.06–3.21)], and 3.76 years [95%CI (3.68–3.87)], half of dabigatran, rivaroxaban and apixaban users had discontinued treatment, respectively (not available for edoxaban).

### Reinitiation

**FIGURE 3:**
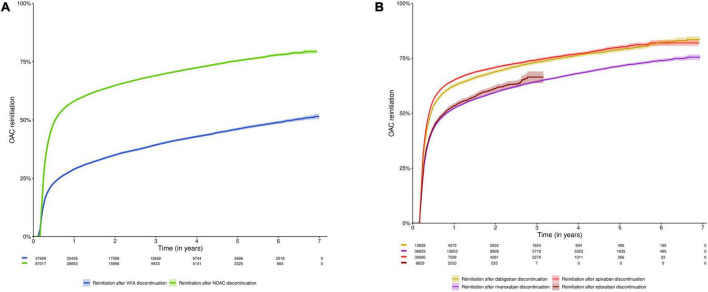
Cumulative incidence curve of reinitiation of an oral anticoagulant after discontinuation of **(A)** NOACs and VKAs; and **(B)** NOAC types (dabigatran, rivaroxaban, apixaban, and edoxaban). Data shown as cumulative incidence with 95% confidence interval and risk table (number of patients at risk). Subjects were included at the date of discontinuation (*n* = 125,873). Subjects were censored in case of death, emigration or end of the study period. The initial lag period in the cumulative incidence curves (60 days for NOACs, 42–60 days for VKAs) is due to the definition of discontinuation (arbitrary supply gap of > 60 days after the calculated last day of supply, with the possibility to extend this gap for VKAs in case of intervening INR testing at least every 42 days). Due to their respective approval in September 2013 and October 2016, and the study period ending on December 31st, 2019, the maximum follow-up duration of apixaban and edoxaban users was limited to 6.3 years and 3.25 years, respectively. NOAC, non-vitamin K antagonist oral anticoagulant; OAC, oral anticoagulant; VKA, vitamin K antagonist.

Half of subjects reinitiated oral anticoagulation within 0.53 years [95%CI (0.53–0.55)] after NOAC discontinuation (*n* = 87,017), as compared to 6.36 years [95%CI (6.13–6.66)] after VKA discontinuation (*n* = 37,609) (*p*-value < 0.001). Among subjects having discontinued NOAC or VKA treatment, 58.1% and 28.9% reinitiated anticoagulation within 1 year, 64.8% and 35.0% within 2 years, and 75.4% and 46.1% within 5 years, respectively (Figure 3A, Supplementary Table 3). In other words, 41.9% of NOAC discontinuers and 71.1% of VKA discontinuers did not reinitiate anticoagulation within 1 year (respectively 24.6% and 53.9% within 5 years).

Among subjects having discontinued dabigatran (*n* = 13,839), rivaroxaban (*n* = 36,663), apixaban (*n* = 30,665) or edoxaban (*n* = 8,631), oral anticoagulation was reinitiated by 62.4%, 52.2%, 65.1%, and 53.4% of subjects within 1 year after dabigatran, rivaroxaban, apixaban and edoxaban discontinuation; 68.9%, 59.5%, 70.8%, and 61.2% within 2 years; and 78.9%, 71.4%, and 80.2% within 5 years (not available for edoxaban), respectively (Figure 3B, Supplementary Table 3).

### Switching

**FIGURE 4:**
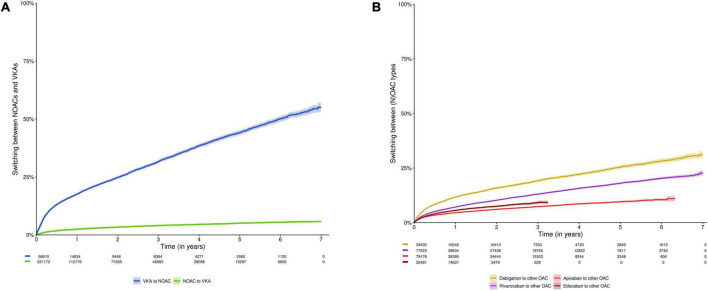
Cumulative incidence curve of switching **(A)** NOACs to VKAs or vice versa; and **(B)** switching between any type of OAC (e.g., from dabigatran to rivaroxaban). Data shown as cumulative incidence with 95% confidence interval and risk table (number of patients at risk). In analysis **(A)**, subjects were censored in case of discontinuation of NOACs or VKAs (allowing switching between NOAC types and VKA types, respectively), death, emigration or end of the study period. In analysis **(B)**, subjects were censored in case of discontinuation of the index OAC type, death, emigration, or end of the study period. Due to their respective approval in September 2013 and October 2016, and the study period ending on December 31st, 2019, the maximum follow-up duration of apixaban and edoxaban users was limited to 6.3 years and 3.25 years, respectively. NOAC, non-vitamin K antagonist oral anticoagulant; OAC, oral anticoagulant; VKA, vitamin K antagonist.

The proportions of subjects switching NOACs to VKAs, and VKAs to NOACs were 2.5% and 17.6% after 1 year, 3.4% and 24.8% after 2 years, and 5.1% and 44.2% after 5 years, respectively (*p*-value < 0.001) (Figure 4A, Supplementary Table 3). The proportions of subjects switching dabigatran, rivaroxaban, apixaban or edoxaban to any other OAC were 11.8%, 7.2%, 4.6%, and 5.6% after 1 year; 15.8%, 10.3%, 6.1%, and 7.5% after 2 years; and 25.4%, 18.0%, and 9.6% after 5 years (not available for edoxaban), respectively (Figure 4B, Supplementary Table 3). The proportions of subjects switching standard to reduced dose NOAC, or vice versa were 14.1% and 15.1% after 1 year, 20.5% and 18.7% after 2 years and 35.5% and 27.7% after 5 years, respectively (Supplementary Figure 2 and Supplementary Table 3).

### Adherence

**FIGURE 5:**
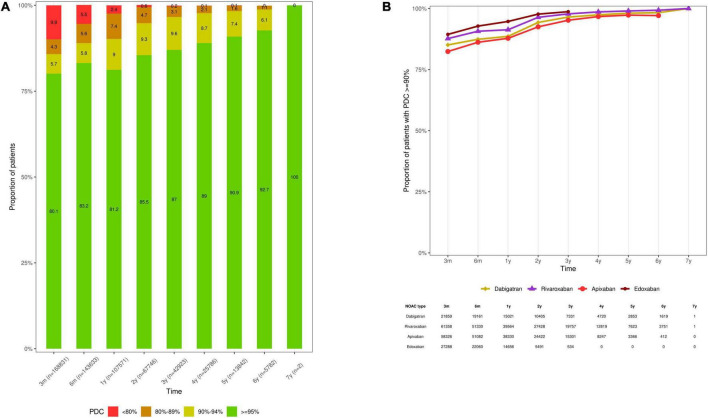
Therapy adherence of **(A)** persistent NOAC users, categorized according to a PDC of < 80%, 80- < 90%, 90- < 94%, and ≥ 95%, and **(B)** the proportion of persistent dabigatran, rivaroxaban, apixaban and edoxaban users with a PDC of ≥ 90% at specific time intervals. Subjects were censored in case of NOAC discontinuation, switching to any other OAC type, death, emigration, or end of the study period. Due to their respective approval in September 2013 and October 2016, and the study period ending on December 31st, 2019, the maximum follow-up duration of apixaban and edoxaban users was limited to 6.3 and 3.25 years, respectively. Only persistent NOAC users with a follow-up at least corresponding with the examined time interval were investigated (e.g., PDC after 1 year in the subgroup of subjects with ≥ 1 year of follow-up), as illustrated by the number of investigated subjects (n) per time point. M, months; NOAC, non-vitamin K antagonist oral anticoagulant; OAC, oral anticoagulant; PDC, proportion of days covered; VKA, vitamin K antagonist; y, years.

Among persistent NOAC users (*n* = 107,571), 2.4%, 7.4%, 9.0%, and 81.2% of subjects had a PDC of < 80%, 80- < 90%, 90- < 95%, and ≥ 95% over 1 year, respectively (mean PDC 97.3% ± 5.8%) (Figure 5A). The proportion of adherent NOAC users (PDC ≥ 90%) was 90.2%, 94.8%, and 98.3% over 1, 2, and 5 years of persistent use, respectively (Supplementary Table 3). Among persistent dabigatran (*n* = 15,021), rivaroxaban (*n* = 39,564), apixaban (*n* = 38,330), and edoxaban users (*n* = 14,656), 88.6%, 91.3%, 87.8%, and 94.7% had a PDC of ≥ 90% over 1 year, respectively (mean PDC 97.0% ± 6.0%, 97.7% ± 5.0%, 96.5% ± 6.8%, and 98.6% ± 4.1%, respectively) (Figure 5B, Supplementary Table 3).

### Predictors of non-persistence and non-adherence

**FIGURE 6:**
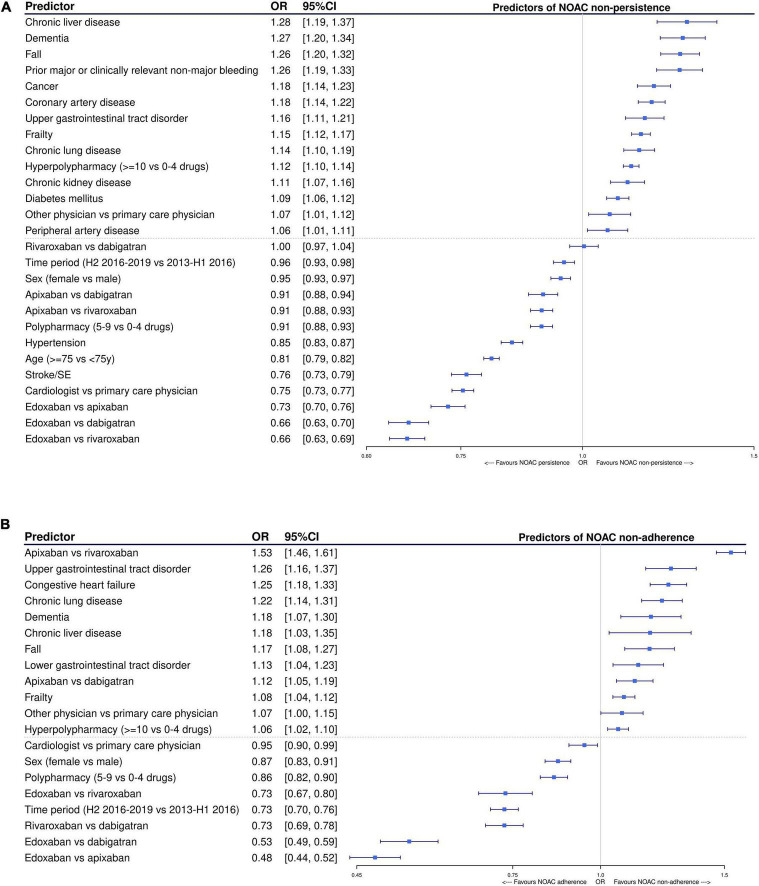
Factors significantly associated with being **(A)** non-persistent and **(B)** non-adherent (PDC < 90%) after 1 year of follow-up in NOAC-treated AF patients after multivariable adjustment, using multivariable logistic regression with backward elimination. Only statistically significant factors were retained in the final model. AF, atrial fibrillation; CI, confidence interval; H1/H2, first/second half-year; NOAC, non-vitamin K antagonist oral anticoagulant; OAC, oral anticoagulant; OR, odds ratio; PDC, proportion of days covered; SE, systemic embolism; VKA, vitamin K antagonist; vs., versus; y, year.

Among NOAC users with ≥ 1 year of follow-up (baseline characteristics summarized in Supplementary Table 4), edoxaban and apixaban were associated with significantly lower odds of non-persistence after 1 year compared to dabigatran [OR 0.66, 95%CI (0.63–0.70); and OR 0.91, 95%CI (0.88–0.94), respectively] and rivaroxaban [OR 0.66, 95%CI (0.63–0.69); and OR 0.91, 95%CI (0.88–0.93), respectively] after multivariable adjustment, while no difference between rivaroxaban and dabigatran was observed [OR 1.00, 95%CI (0.97–1.04)] (Figure 6A). Compared to apixaban, edoxaban was also associated with a significantly lower odds of non-persistence [OR 0.73, 95%CI (0.70–0.76)]. Other factors positively associated with persistence after multivariable adjustment were having a prescription from a cardiologist (compared to primary care physician); being treated in the second half of the study period (2016–2019); being older (≥ 75 years) or female; having hypertension or prior thromboembolism; and using 5–9 drugs (polypharmacy compared to no polypharmacy). Factors associated with significantly higher odds of non-persistence were having dementia, being frail or having recently fallen; having chronic liver, kidney or lung disease, cancer, vascular disease, diabetes, upper gastrointestinal tract disorders or previous major or clinically relevant non-major bleeding; having a prescription from another secondary care physician; and using ≥ 10 drugs (hyperpolypharmacy compared to no polypharmacy).

Among subjects persistent to NOACs for at least 1 year, edoxaban was associated with a significantly lower odds of non-adherence (PDC < 90%) during that year compared to dabigatran [OR 0.53, 95%CI (0.49–0.59)], rivaroxaban [OR 0.73, 95%CI (0.67–0.80)], and apixaban [OR 0.48, 95%CI (0.44–0.52)] after multivariable adjustment (Figure 6B). Moreover, rivaroxaban was also associated with a significantly lower odds of non-adherence compared to dabigatran [OR 0.73, 95%CI (0.69–0.78)], whereas apixaban was associated with a significantly higher odds of non-adherence compared to dabigatran [OR 1.12, 95%CI (1.05–1.19)] and rivaroxaban [OR 1.53, 95%CI (1.46–1.61)]. Other factors significantly associated with high adherence were having a prescription from a cardiologist, being recently treated, being female, and using 5–9 drugs compared to no polypharmacy. Factors associated with significantly higher odds of non-adherence were being dement, frail or having recently fallen; having chronic liver or lung disease, upper or lower gastrointestinal tract disorders, or heart failure; having a prescription from another secondary care physician; and using ≥ 10 drugs compared to no polypharmacy.

### Sensitivity analyses

Trends were consistent using a > 30-day or > 90-day permissible gap to define discontinuation (although persistence was considerably lower using the more stringent 30-day gap, especially among VKA users; Supplementary Figures 3, 4), when considering ambulatory and hospital prescription claims for the PDC calculation (Supplementary Figure 6), and when investigating AF subjects at high thromboembolic risk (Supplementary Figures 7–10), subjects having initiated treatment between October 2016 and December 2019 (Supplementary Figures 11–14), and subjects with ≥ 1 year of follow-up (Supplementary Figures 15–18). However, when assessing adherence in persistent and non-persistent NOAC users, the proportion of adherent users (PDC ≥ 90%) was considerably lower and decreased over time (e.g., 66.6%, 62.8%, and 60.2% over 1, 2, and 5 years of persistent and non-persistent use) (Supplementary Figure 5A). Nevertheless, higher adherence to edoxaban than other NOACs was still observed (e.g., 64.3%, 65.1%, 66.0%, and 75.7% of dabigatran, rivaroxaban, apixaban, and edoxaban users had a PDC of ≥ 90% over 1 year, respectively) (Supplementary Figure 5B).

## Discussion

In this nationwide cohort study including nearly 280,000 AF subjects, we have demonstrated that long-term persistence to NOACs was substantially higher than to VKAs (70% versus 37% after 1 year), that most (N)OAC users quickly reinitiated treatment after discontinuation and that VKAs were more frequently switched to NOACs than vice versa. Moreover, adherence and persistence to individual NOACs were very high and absolute differences between NOACs small. However, better adherence to once-daily than twice-daily dosed NOACs (high adherence in 91% of rivaroxaban and 95% of edoxaban users after 1 year versus 89% of dabigatran and 88% of apixaban users), and higher persistence to edoxaban and apixaban than to dabigatran or rivaroxaban were observed (77% and 70% versus 68% and 67% after 1 year, respectively). To the best of our knowledge, this is the first nationwide cohort study with long-term follow-up investigating adherence and persistence between NOAC types since the approval of edoxaban.

Despite the lack of monitoring and less regular medical follow-up, higher persistence to NOACs than VKAs has been consistently observed in different settings using variable definitions of discontinuation, although persistence rates to VKAs should be interpreted with caution due to their variable dosing regimens (13–16). The risk of discontinuation tends to be highest in the first 6–12 months of treatment, especially among VKA users, followed by a more comparable and gradual decline in persistence, as observed before (8, 9, 17, 18). Besides guideline recommendations and direct-to-prescriber marketing by pharmaceutical NOAC companies, lower persistence to, and predominant switching of VKAs may be due to poor patient satisfaction and high burden of treatment, given their complex dosing regimens, need for frequent coagulation monitoring, dietary and drug interactions, and anxiety for bleeding (1, 14, 38). Indeed, switching VKAs to NOACs has shown to increase patient satisfaction (38).

Differences in persistence to individual NOACs were in line with previous studies, which have demonstrated higher persistence to apixaban than to rivaroxaban or dabigatran (8, 11, 13–15, 17, 24, 34, 39, 40), except for one study (41) in which persistence to rivaroxaban was highest. Likewise, other studies also observed predominant switching of dabigatran followed by rivaroxaban (30, 39–41). Frequent switching and discontinuation of dabigatran may be due to its high dependency on renal clearance, specific side effects such as dyspepsia, longest market approval (resulting in increased switching when other NOACs became available) (40), twice-daily dosing regimen, and dabigatran being administered in capsules sensitive to moisture which should not be opened or repackaged to other dispensing systems (1, 11, 18, 34, 39–42).

However, discontinuation of anticoagulation was common in our study. Suggested reasons for treatment discontinuation include physician choice (e.g., due to high bleeding risks or underestimated stroke risks), patient preference (e.g., refusing chronic treatment or fear for bleeding complications), adverse drug reactions (e.g., bleeding events), restoration of sinus rhythm after cardioversion or ablation, invasive procedures or (rarely) treatment costs (6, 11, 12, 17, 18, 28, 43, 44). However, most subjects discontinue treatment without a precise reason (18, 43, 44). The importance of long-term persistence to anticoagulants should not be underestimated, given that non-persistence has been associated with significantly increased thromboembolic and mortality risks without influencing major bleeding risks (7–12). Ischemic strokes may occur soon after OAC discontinuation (e.g., within 7–9 days), due to a potential prothrombotic rebound phenomenon (44). Consequently, even short treatment gaps may expose patients to increased thromboembolic risks which could have been avoided.

Although most subjects quickly reinitiated treatment after discontinuation, more than half of subjects at intermediate-to-high thromboembolic risk did not reinitiate oral anticoagulation within 5 year after VKA discontinuation, and one-fourth of subjects after NOAC discontinuation. Lower reinitiation rates with VKAs than NOACs may be due to higher intracranial bleeding risks with VKAs (reluctance to reinitiate treatment after the most feared complication of anticoagulation), and NOACs having a positive net clinical benefit over stopping anticoagulation even in the oldest AF patients as opposed to VKAs (40, 42, 45). The latter has been confirmed by a growing body of evidence on the preserved effectiveness and safety of NOACs in several (vulnerable) patient subgroups over recent years (42, 45). Likewise, potential differences in safety outcomes among older AF patients may have contributed to differences in persistence, switching, and reinitiation of individual NOACs, due to a reluctance to use certain anticoagulants in vulnerable patients (11, 42). However, since apixaban and edoxaban have only been available since September 2013 and October 2016 respectively, whereas dabigatran and rivaroxaban since August and September 2012, differences in approval dates may have impacted our results. Physicians may be more familiar and confident with the use of NOACs over time, resulting in less discontinuation (15, 39). However, trends were consistent after restricting the study population to subjects having initiated treatment between October 2016 and December 2019, when all NOACs were commercially available in Belgium.

Importantly, > 90% of persistent NOAC users were adherent for ≥ 90% of the time during their first year of use, which was slightly more pronounced with once-daily dosed NOACs. Aiming for a high adherence to anticoagulants is crucial, as recent studies have shown that the risk of adverse outcomes was lowest in patients with a PDC of ≥ 90% (7, 8). Although the observed high adherence to NOACs is in line with previous literature (6, 8, 10, 12, 24, 34, 41), results on differences in adherence to individual NOACs are more conflicting, with some studies (6, 13, 24, 34) favoring adherence to once-daily dosed NOACs, whereas other studies (7, 10, 30) described a higher adherence to twice-daily dosed apixaban. However, missing one dose of twice-daily dosed NOACs may not result in a comparably reduced thromboembolic protection as missing one dose of once-daily dosed NOACs, especially since once-daily dosed rivaroxaban and edoxaban actually have the shortest half-lives of NOACs (6, 24, 46). Exemplary, Vrijens et al. simulated that a single missed dose of once-daily dosed NOACs equals about 2–3 consecutively missed doses of twice-daily dosed NOACs (6, 46). Consequently, higher adherence thresholds for once-daily than twice-daily dosed NOACs may be necessary, pending future research (6). Nevertheless, since absolute differences were small, these results should not be overemphasized. Moreover, it should be mentioned that the observed high adherence rates were in part driven by the assessment of adherence in persistent users only, in accordance with prior research (8, 32–34). When assessing adherence in persistent and non-persistent NOAC users, adherence was substantially lower and decreased over time, although higher adherence to edoxaban than other NOACs was still observed. However, these results reflect the combined effect of subjects having discontinued or permanently stopped treatment and subjects being non-adherent while treated, which makes it difficult to discriminate non-adherence from non-persistence (8). Therefore, these additional findings should be interpreted with caution and highlight the need for standardized approaches to assess adherence across different studies, although some efforts have already been made (32, 33).

Of note, NOAC persistence and adherence were significantly higher in more recent years and among older, female subjects with polypharmacy, history of thromboembolism, and a prescription from cardiologists, whereas significantly lower among patients with geriatric traits, renal dysfunction, bleeding risk factors, chronic lung disease (potentially driven by a subgroup of smokers), hyperpolypharmacy and a prescription from other secondary care physicians. Although these observations are mostly in line with prior research (6–9, 11, 16, 17, 34, 40), results on the impact of female sex are more conflicting, as some studies observed higher persistence rates in male AF patients (13, 24, 27).

Our results highlight that there is still room for improvement in real life practice. Therefore, whenever anticoagulants are initiated, patients should be educated on the importance of long-term persistence and good adherence, informed about adherence aids and instructed what to do in case of (minor) bleeding (e.g., contacting their prescribing physician instead of discontinuing treatment) in a multidisciplinary, patient-centered approach (1, 38). During each visit, adherence and treatment satisfaction should be checked (1, 6, 38).

### Strengths and limitations

Strengths of this study include the 7-year follow-up, investigation of real-world persistence, reinitiation, switching, and adherence on a full-population scale including many edoxaban users and difficult-to-reach subgroups (e.g., subjects with dementia), use of INR test dates to reduce misclassification of VKA discontinuation, consideration of stockpiling to identify supply gaps, and assessment of anticoagulant dispensing in ambulatory and hospital care. However, some limitations should be acknowledged. First, assessment of adherence and persistence to OACs was based on dispensing data, not on the patients’ actual intake and physicians’ prescriptions. Therefore, we could not distinguish between patients with extended treatment gaps (drug holidays), patients with frequently missed doses and treatment interruption by physicians (non-prescription). Second, due to missing INR values and considering fixed DDDs as the recommended dosing regimen of VKAs (29) (whereas dosing is highly variable in clinical practice), persistence to VKAs is likely underestimated. Nevertheless, trends were consistent using different supply gaps to define discontinuation. Third, due to variable approval dates and follow-up durations, persistence and adherence to edoxaban may be less comparable to other NOACs, as clinical practice may have changed over time (e.g., thanks to the increased experience and confidence with the use of NOACs) (13). Nevertheless, trends were consistent when restricting the study population to subjects having initiated treatment between October 2016 and December 2019, or in the subgroup of subjects with ≥ 1 year of follow-up. Fourth, we could not identify personal reasons for, and outcomes associated with non-persistence or non-adherence, as this was beyond the scope of our study. This may have affected our results on the predictors of non-adherence and non-persistence, as we could not distinguish patient-driven non-adherence or physician-recommended treatment interruption (e.g., due to major bleeding). Fifth, since adherence was assessed in persistent NOAC users only to avoid confusing non-adherence with non-persistence, adherence rates increased over time and may have been overestimated, given that subjects with low adherence in the first months/years of therapy who subsequently discontinued treatment, were excluded at later time intervals. In a sensitivity analysis investigating persistent and non-persistent NOAC users, lower and decreasing adherences rates over time were observed, though relative differences between individual NOACs remained. Sixth, coding errors, misclassification bias, and unmeasured confounding may be present due to our observational design using healthcare databases. However, by identifying comorbidities based on ICD, ATC and/or medical procedure codes assessed in ambulatory and hospital care, missing data and misclassification of characteristics were reduced. Seventh, the number of OAC-naïve AF patients may have been overestimated, since subjects having used and discontinued an OAC > 1 year before the start of our study would not have been identified as OAC-experienced. Lastly, data on certain lifestyle characteristics (e.g., weight, smoking) were not available or derived indirectly (e.g., alcohol abuse).

## Conclusion

Persistence to NOACs was substantially higher compared to VKAs, (N)OAC users quickly reinitiated treatment after discontinuation, and VKAs were more frequently switched to NOACs than vice versa. Moreover, adherence and persistence to individual NOACs were very high, especially in older females treated by cardiologists in recent years. Although absolute differences were small, persistence to edoxaban and apixaban was higher than to dabigatran or rivaroxaban, whereas adherence was higher with once-daily than twice-daily dosed NOACs. However, as 10% of persistent NOAC users were non-adherent in their first year of treatment and one-fourth of subjects did not reinitiate anticoagulation within 5 years after NOAC discontinuation, opportunities to further improve therapy adherence among AF patients should be taken.

## Data availability statement

The data analyzed in this study was obtained from the InterMutualistic Agency (IMA) database and Minimal Hospital Dataset (MHD) and access is subject to approval. Requests to access these datasets should be directed to the administrators of https://ima-aim.be/ and https://www.health.belgium.be/en/node/23607.

## Ethics statement

The studies involving human participants were reviewed and approved by the InterMutualistic Agency database and Minimal Hospital Dataset administrators and by the “Sectoral Committee of Social Security and Health, Section Health,” a subcommittee of the Belgian Commission for the Protection of Privacy (approval code IVC/KSZG/20/344). Written informed consent for participation was not required for this study in accordance with the national legislation and the institutional requirements.

## Author contributions

MG and LL contributed to the concept and design of the study. MG performed the statistical analysis, interpretation, and writing under the supervision of LL. AC, SS, EM, KB, TDB, and LL revised the manuscript critically. All authors contributed to the article and approved the final version of the manuscript.
