# Supplementary material for: Adherence and persistence to oral anticoagulants in patients with atrial fibrillation: A Belgian nationwide cohort study
Source: Front Cardiovasc Med. 2022 Sep 29;9:994085. doi: 10.3389/fcvm.2022.994085 (PMC9558210; doi:10.3389/fcvm.2022.994085)
Supplement: Supplementary file 1 [file Data_Sheet_1.DOCX]

**Supplementary materials**

Table of contents

[Supplemental tables 3](#_Toc113007796)

[eTable 1: STROBE reporting guideline 3](#_Toc113007797)

[eTable 2: Definition of in- and exclusion criteria, comorbidities, comedication, prescribing physician’s specialty and clinical risk scores 5](#_Toc113007798)

[eTable 3: Persistence, reinitiation, switching and adherence rates over time 14](#_Toc113007799)

[eTable 4: Baseline characteristics (subgroup of subjects with ≥1 year of follow-up) 15](#_Toc113007800)

[Supplemental figures 17](#_Toc113007801)

[eFigure 1: Overview of study design 17](#_Toc113007802)

[eFigure 2: Switching between NOAC doses 18](#_Toc113007803)

[eFigure 3: Persistence (30-day gap) (sensitivity analysis) 19](#_Toc113007804)

[eFigure 4: Persistence (90-day gap) (sensitivity analysis) 21](#_Toc113007805)

[eFigure 5: Adherence (PDC) in persistent and non-persistent NOAC users (sensitivity analysis) 23](#_Toc113007806)

[eFigure 6: Adherence (PDC) (ambulatory and hospital dispensings) (sensitivity analysis) 25](#_Toc113007807)

[eFigure 7: Persistence in subjects with a non-sex-related CHA_2_DS_2_-VASc score of ≥2 (sensitivity analysis) 27](#_Toc113007808)

[eFigure 8: Reinitiation in subjects with a non-sex-related CHA_2_DS_2_-VASc score of ≥2 (sensitivity analysis) 29](#_Toc113007809)

[eFigure 9: Switching in subjects with a non-sex-related CHA_2_DS_2_-VASc score of ≥2 (sensitivity analysis) 31](#_Toc113007810)

[eFigure 10: Adherence (PDC) in subjects with a non-sex-related CHA_2_DS_2_-VASc score of ≥2 (sensitivity analysis) 33](#_Toc113007811)

[eFigure 11: Persistence in subjects having initiated treatment between October 2016 and December 2019 (sensitivity analysis) 35](#_Toc113007812)

[eFigure 12: Reinitiation in subjects having initiated treatment between October 2016 and December 2019 (sensitivity analysis) 37](#_Toc113007813)

[eFigure 13: Switching in subjects having initiated treatment between October 2016 and December 2019 (sensitivity analysis) 39](#_Toc113007814)

[eFigure 14: Adherence (PDC) in subjects having initiated treatment between October 2016 and December 2019 (sensitivity analysis) 41](#_Toc113007815)

[eFigure 15: Persistence in subjects with ≥1 year of follow-up (sensitivity analysis) 43](#_Toc113007816)

[eFigure 16: Reinitiation in subjects with ≥1 year of follow-up (sensitivity analysis) 45](#_Toc113007817)

[eFigure 17: Switching in subjects with ≥1 year of follow-up (sensitivity analysis) 47](#_Toc113007818)

[eFigure 18: Adherence (PDC) in subjects with ≥1 year of follow-up (sensitivity analysis) 49](#_Toc113007819)

[References 51](#_Toc113007820)

# Supplemental tables

## eTable 1: STROBE reporting guideline

|  | **Item No.** | **Recommendation** | **Page No.** |
| --- | --- | --- | --- |
| **Title and abstract** | 1 | (*a*) Indicate the study’s design with a commonly used term in the title or the abstract | 1-2 |
|  |  | (*b*) Provide in the abstract an informative and balanced summary of what was done and what was found | 2 |
| **Introduction** | | |  |
| Background/rationale | 2 | Explain the scientific background and rationale for the investigation being reported | 3 |
| Objectives | 3 | State specific objectives, including any prespecified hypotheses | 3 |
| **Methods** | | |  |
| Study design | 4 | Present key elements of study design early in the paper | 4 |
| Setting | 5 | Describe the setting, locations, and relevant dates, including periods of recruitment, exposure, follow-up, and data collection | 4-5 |
| Participants | 6 | (*a*) Give the eligibility criteria, and the sources and methods of selection of participants. Describe methods of follow-up | 4-5 |
|  |  | (*b*) For matched studies, give matching criteria and number of exposed and unexposed | / |
| Variables | 7 | Clearly define all outcomes, exposures, predictors, potential confounders, and effect modifiers. Give diagnostic criteria, if applicable | 5-6, eTable 2 |
| Data sources/ measurement | 8* | For each variable of interest, give sources of data and details of methods of assessment (measurement). Describe comparability of assessment methods if there is more than one group | 4-6 |
| Bias | 9 | Describe any efforts to address potential sources of bias | 7-8 |
| Study size | 10 | Explain how the study size was arrived at | 4-5 |
| Quantitative variables | 11 | Explain how quantitative variables were handled in the analyses. If applicable, describe which groupings were chosen and why | 7-8 |
| Statistical methods | 12 | (*a*) Describe all statistical methods, including those used to control for confounding | 7-8 |
|  |  | (*b*) Describe any methods used to examine subgroups and interactions | 7-8 |
|  |  | (*c*) Explain how missing data were addressed | 7 |
|  |  | (*d*) If applicable, explain how loss to follow-up was addressed | / |
|  |  | (*e*) Describe any sensitivity analyses | 7-8 |
| **Results** | | |  |
| Participants | 13* | (a) Report numbers of individuals at each stage of study—eg numbers potentially eligible, examined for eligibility, confirmed eligible, included in the study, completing follow-up, and analysed | 9, Table 1 |
|  |  | (b) Give reasons for non-participation at each stage | / |
|  |  | (c) Consider use of a flow diagram | Figure 1 |
| Descriptive data | 14* | (a) Give characteristics of study participants (eg demographic, clinical, social) and information on exposures and potential confounders | Table 1 |
|  |  | (b) Indicate number of participants with missing data for each variable of interest | Table 1 |
|  |  | (c) Summarise follow-up time (eg, average and total amount) | Table 1 |
| Outcome data | 15* | Report numbers of outcome events or summary measures over time | 9-11, eTable 3 |
| Main results | 16 | (*a*) Give unadjusted estimates and, if applicable, confounder-adjusted estimates and their precision (eg, 95% confidence interval). Make clear which confounders were adjusted for and why they were included | 9-11 |
|  |  | (*b*) Report category boundaries when continuous variables were categorized | 9-11 |
|  |  | (*c*) If relevant, consider translating estimates of relative risk into absolute risk for a meaningful time period | 9-10 |
| Other analyses | 17 | Report other analyses done—eg analyses of subgroups and interactions, and sensitivity analyses | 11 |
| **Discussion** | | |  |
| Key results | 18 | Summarise key results with reference to study objectives | 12 |
| Limitations | 19 | Discuss limitations of the study, taking into account sources of potential bias or imprecision. Discuss both direction and magnitude of any potential bias | 14-15 |
| Interpretation | 20 | Give a cautious overall interpretation of results considering objectives, limitations, multiplicity of analyses, results from similar studies, and other relevant evidence | 11-15 |
| Generalisability | 21 | Discuss the generalisability (external validity) of the study results | 11-15 |
| **Other information** | | |  |
| Funding | 22 | Give the source of funding and the role of the funders for the present study and, if applicable, for the original study on which the present article is based | 17 |

*Give information separately for exposed and unexposed groups.

**eTable 1:** Compliance to the STROBE (Strengthening the Reporting of Observational Studies in Epidemiology) reporting guideline.^1^

## eTable 2: Definition of in- and exclusion criteria, comorbidities, comedication, prescribing physician’s specialty and clinical risk scores

| **VARIABLE** | | | | **ICD, ATC AND MEDICAL PROCEDURE CODES** |
| --- | --- | --- | --- | --- |
| **INCLUSION CRITERIA** | | | | |
| OAC use (NOAC or VKA) | | | | **ATC:** B01AE07, B01AF01, B01AF02, B01AF03, B01AA03, B01AA04, B01AA07 |
| ≥45 years | | | | Age ≥45 years on index date |
| ≥1 year coverage | | | | ≥1 year coverage by a Belgian health insurance fund |
| **EXCLUSION CRITERIA** | | | | |
| Recent total hip/knee replacement surgery | | | | **Medical procedure group code:** N32 (≤6 months before index date) |
| Recent venous thromboembolism  (deep vein thrombosis or pulmonary embolism) | | | | **ICD-9:** 451.1, 451.2, 451.81, 451.89, 451.9, 452, 453.2, 453.3, 453.4, 453.5, 453.77, 453.79, 453.87, 453.89, 671.3, 671.4 (≤6 months before index date)  **ICD-10:** I80.1, I80.2, I80.3, I80.8, I80.9, I81, I82.2, I82.3, I82.4, I82.5, I82.89, I82.9, O22.3, O22.5, O87.1, O87.3 (≤6 months before index date) |
| Valvular atrial fibrillation | | | |  |
|  | | | Moderate-severe mitral stenosis | **ICD-9:** 394.0, 394.2, 396.0, 396.1, 746.5  **ICD-10:** I05.0, I05.2, I34.2, Q23.2 |
|  | | | Mechanical prosthetic heart valve | **ICD-9:** V43.3  **ICD-10:** Z95.2  **Medical procedure code:** 159110, 159121, 159132, 159143, 159154, 159165 |
| End-stage renal disease | | | |  |
|  | | | CKD stage V (without dialysis) | **ICD-9:** 403.01, 403.11, 403.91, 404.02, 404.12, 404.92, 585.5, 585.6, 586  **ICD-10:** N18.5, N18.6, N19, I12.0, I13.11 |
|  | | | Dialysis | **ICD-9:** V45.11, V56  **ICD-10:** Z49, Z99.2  **Medical procedure group code:** N81 |
| **DEMOGRAFICS (on index date)** | | | | |
| Age | | | | Age on index date based on the year and month of birth, not the exact date due to patient privacy. |
| Sex | | | | Sex on index date |
| **COMORBIDITIES (≤1 year before index date)**^2-4^ | | | | |
| Hypertension | | | | **ICD-9:** 401-405, 437.2  **ICD-10:** I10-I13, I15, I16, I67.4  **ATC:** combination treatment with ≥2 of the following drug classes:  **I) Cardioselective beta blocker:** C07AB, C07AG, C07BB, C07BG, C07CB, C07CG, C07DB, C07FB, C07FX03, C07FX04, C07FX05, C07FX06, C09BX02, C09BX04, C09BX05, C09DX05  **II) ACE inhibitor or angiotensin II receptor blocker:** C09A, C09B, C09C, C09D, C10BX04, C10BX06, C10BX07, C10BX10, C10BX11, C10BX12, C10BX13, C10BX14, C10BX15, C10BX16, C10BX17, C10BX18  **III) Calcium channel blocker:** C07FB, C08C, C08G, C09BB, C09DB, C09BX01, C09BX03, C09BX04, C09DX01, C09DX03, C09DX06, C09DX07, C09XA53, C09XA54, C10BX03, C10BX07, C10BX09, C10BX11, C10BX14, C10BX18  **IV) Non-loop diuretic:** C02L, C03A, C03BA, C03BB, C03EA, C07B, C07C, C07D, C08GA, C09BA, C09BX01, C09BX03, C09DA, C09DX01, C09DX03, C09DX06, C09DX07, C09XA52, C09XA54, C10BX13, C03D, C03EA, C03EB  **V) Other antihypertensive (alpha adrenergic blocker, vasodilator)**: C02A, C02B, C02C, C02DB, C02DD, C02DG, C02L |
| Coronary artery disease  (stable or recent myocardial infarction) | | | | **ICD-9:** 410, 411, 412, 413, 414, 429.2, 429.7, V45.81, V45.82  **ICD-10:** I20, I21, I22, I23, I24, I25, Z95.1, Z95.5, Z98.61  **Medical procedure code:** 158992, 159003, 159014, 159025, 159036, 159040, 229515, 229526, 229574, 229585, 229611, 229622, 229633, 229644, 589013, 589024, 589153, 589164, 589934, 589945, 589956, 589960, 680315, 680326, 680352, 680363, 687875, 687886 |
| Congestive heart failure | | | | **ICD-9:** 398.91, 402.01, 402.11, 402.91, 404.01, 404.03, 404.11, 404.13, 404.91, 404.93, 425.4–425.9, 428  **ICD-10:** I09.81, I11.0, I13.0, I13.2, I42.0, I42.6-I42.9, I43, I50  **ATC:** combination treatment of all of the following drug classes:  **I) Cardioselective beta blocker:** C07AB, C07AG, C07BB, C07BG, C07CB, C07CG, C07DB, C07FB, C07FX03, C07FX04, C07FX05, C07FX06, C09BX02, C09BX04, C09BX05, C09DX05  **II) ACE-inhibitor or ARB:** C09A, C09B, C09C, C09D, C10BX04, C10BX06, C10BX07, C10BX10, C10BX11, C10BX12, C10BX13, C10BX14, C10BX15, C10BX16, C10BX17, C10BX18  **III) Potassium-sparing diuretic:** C03D, C03EA, C03EB  **IV) Loop diuretic:** C03C, C03EB |
| Valvular heart disease  (aortic, mitral or other/non-specific valve disease, valve repair, bioprosthetic heart valve) *(except for moderate-severe mitral stenosis or mechanical prosthetic heart valve)* | | | | **ICD 9:** 036.42, 074.22, 093.2, 098.84, 112.81, 391.1, 394.1, 394.2, 394.9, 395.0, 395.1, 395.2, 395.9, 396, 397, 421, 424.0, 424.1, 424.2, 424.3, 424.9, 746.0, 746.1, 746.3, 746.4, 746.6, V42.2  **ICD 10:** A32.82, A39.51, A52.03, A54.83, B33.21, B37.6, I01.1, I05.1, I05.2, I05.8, I05.9, I06.0, I06.1, I06.2, I06.8, I06.9, I07, I08, I09.1, I09.89, I33, I34.0, I34.1, I34.8, I34.9, I35.0, I35.1, I35.2, I35.8, I35.9, I36, I37, I38, I39, M32.11, Q22, Q23.0, Q23.1, Q23.3, Q23.8, Q23.9, Z95.3, Z95.4  **Medical procedure code:** 159176, 159180, 159191, 159202, 159213, 159224, 159235, 159246, 159250, 159261, 159272, 159283, 159294, 159305, 170634, 170645, 172491, 172502, 172513, 172524, 172734, 172745, 172756, 172760, 172771, 172782, 172955, 172966, 172970, 172981, 172992, 173003, 229515, 229526, 229596, 229600, 589190, 589201, 680153, 680164, 680175, 680186, 680993, 681004, 684736, 684740, 688192, 688203, 691950, 691961, 704616, 704620, 704631, 704642, 704653, 704664 |
| Peripheral artery disease | | | | **ICD-9:** 440, 441, 443.89, 443.9, 444, 447.1, 557.1, 557.9, V43.4  **ICD-10:** I70, I71, I73.8, I73.9, I74, I77.1, I79.0, K55.1, K55.8, K55.9, Z95.82, Z98.62  **Medical procedure code:** 229294, 229305, 229316, 229320, 229331, 229342, 235071, 235082, 235093, 235104, 235115, 235126, 235196, 235200, 235211, 235222, 236014, 236025, 236036, 236040, 236051, 236062, 237016, 237020, 237031, 237042, 237053, 237064, 237075, 237086, 237090, 237101, 237171, 237182, 589050, 589061, 589094, 589105, 589175, 589186, 589595, 589606, 589610, 589621, 589632, 589643, 589654, 589665 |
| Dyslipidemia | | | | **ICD-9:** 272.0, 272.1, 272.3, 272.4, 272.5, 272.8, 272.9  **ICD-10:** E78  **ATC:** C10 |
| Chronic kidney disease  (CKD stage III-IV, renal transplant, other/non-specific CKD) *(except for CKD stage V or dialysis)* | | | | **ICD-9:** 249.4, 250.4, 403.00, 403.10, 403.90, 404.00, 404.10, 404.90, 580, 581, 582, 583, 584, 585.3, 585.4, 585.9, 586, 588, 590.0, 753.12-753.15, 996.81, V42.0  **ICD-10:** E08.2, E09.2, E10.2, E11.2, E13.2, I12.9, I13.10, M32.14, M32.15, N00, N01, N02, N03, N04, N05, N07, N11, N14, N15.0, N17, N18.3, N18.4, N18.9, N19, N25, Q61.1-Q61.4, T86.1, Z94.0  **Medical procedure code:** 107096, 107111, 107133, 107155, 318010, 318021, 318290, 318301, 754294, 757433, 757492 |
| Chronic liver disease  (mild, moderate-severe, cirrhosis) | | | | **ICD-9:** 070.0, 070.2, 070.3, 070.4, 070.51, 070.52, 070.54, 070.6, 070.70, 070.71, 155.0, 155.1, 155.2, 197.7, 456.0, 456.1, 456.2, 567.23, 570, 571, 571.2, 571.5, 571.6, 572.2, 572.3, 572.4, 572.8, 573.0, 573.5, 573.8, 573.9, 789.59, V42.7  **ICD-10:** B15.0, B16.0, B16.2, B17.0, B17.10, B17.11, B18, B19.0, B19.10, B19.11, B19.20, B19.21, C22, C78.7, I85, I86.4, K65.2, K70.0, K70.1, K70.2, K70.3, K70.4, K70.9, K71.1, K71.3, K71.4, K71.5, K71.6, K71.7, K71.8, K71.9, K72, K73, K74, K75.3, K75. 4, K75.8, K75.9, K76.0, K76.1, K76.2, K76.5, K76.6, K76.7, K76.81, K76.89, K76.9, K77, R18.8, Z94.4  **ATC:** J05AB04, J05AF05, J05AF07, J05AF08, J05AF10, J05AE11, J05AE12, J05AE14, J05AX15, J05AX65, J05AP  **Medical procedure code:** 318076, 318080, 318334, 318345, 472113, 472124, 556754, 556765, 589352, 589363 |
| Chronic lung disease  (COPD, asthma, other) | | | | **ICD-9**: 416, 491-496, 500-505, 506.4, 508.1, 515, 516.3, 516.9, 518.1, 518.2, 518.83, 518.84  **ICD-10**: I27, J41-J45, J47, J60-J67, J68.4, J70.1, J70.3, J84.1, J84.9, J96.1, J96.2, J98.2, J98.3  **ATC:** R03DC, R03DX |
| Obstructive sleep apnea | | | | **ICD-9:** 327.23  **ICD-10**: G47.33  **Medical procedure code:** 765951, 779870, 779881, 779892, 779903, 779914, 779925, 779936, 779951, 788012, 788023 |
| Cancer | | | | **ICD-9:** 140-209, 223, 230-239, 258.0, V58.0, V58.11, V58.12  **ICD-10:** C00-C96, D00-D09, D37-D49, E31.2, Z51.0, Z51.11, Z51.12  **ATC:** L01  **Medical procedure code:** 154873, 154884, 154895, 154906, 157231, 157242, 201191, 201202, 201213, 201224, 220275, 220286, 220371, 220382, 201213, 201224, 226914, 226925, 226936, 226940, 227216, 227220, 227275, 227286, 227636, 227640, 227651, 227662, 227673, 227684, 227695, 227706, 227710, 227721, 227732, 227743, 227754, 227765, 227776, 227780, 227791, 227802, 227813, 227824, 227835, 227846, 228012, 228023, 228174, 228185, 228233, 228244, 228255, 228266, 228270, 228281, 228292, 228303, 228314, 228325, 228336, 228340, 230473, 230484, 231033, 231044, 241231, 241242, 241415, 241426, 241430, 241441, 241452, 241463, 241555, 241566, 242012, 242023, 242034, 242045, 242292, 242303, 242314, 242325, 242830, 242841, 242852, 242863, 242874, 242885, 242896, 242900, 243051, 243062, 243073, 243084, 243235, 243246, 243736, 243740, 243751, 243762, 243773, 243784, 244016, 244020, 244031, 244042, 244075, 244086, 244790, 244801, 244856, 244860, 244893, 244904, 244915, 244926, 244930, 244941, 244952, 244963, 244974, 244985, 245512, 245523, 245534, 245545, 246050, 246061, 246072, 246083, 247111, 247122, 247133, 247144, 251753, 251764, 251775, 251786, 254892, 254903, 256115, 256126, 256336, 256340, 256572, 256583, 257191, 257202, 258355, 258366, 258370, 258381, 258392, 258403, 258451, 258462, 258554, 258565, 258856, 258860, 258871, 258882, 258893, 258904, 259033, 259044, 259114, 259125, 260190, 260201, 260411, 260422, 260433, 260444, 260551, 260562, 260654, 260665, 260750, 260761, 261111, 261122, 261391, 261402, 261472, 261483, 261671, 261682, 261774, 261785, 261796, 261800, 262334, 262345, 262570, 262581, 277756, 277760, 277771, 277782, 278795, 278806, 278810, 278821, 281831, 281842, 281956, 281960, 282310, 282321, 282671, 282682, 284056, 284060, 288455, 288466, 288470, 288481, 289892, 289903, 291056, 291060, 310494, 310505, 311312, 311323, 312550, 312561, 312572, 312583, 312594, 312605, 312653, 312664, 312970, 312981, 350114, 350125, 350136, 350140, 350276, 350280, 350291, 350302, 350372, 350383, 350674, 350685, 350696, 350700, 431174, 431185, 431336, 431340, 431351, 431362, 432294, 432305, 444113, 444124, 444135, 444146, 444150, 444161, 444172, 444183, 444194, 444205, 444216, 444220, 444231, 444242, 444253, 444264, 444275, 444286, 444290, 444301, 444312, 444323, 444334, 444345, 444474, 444485, 444592, 444603, 473970, 473981, 474795, 474806, 565073, 565084, 565095, 565106, 565110, 565121, 565132, 565143, 565154, 565165, 587834, 587845, 587871, 587882, 587893, 587904, 587915, 587926, 588431, 588442, 588453, 588464, 588475, 588486, 588490, 588501, 588512, 588523, 588534, 588545, 588556, 588560, 588571, 588582, 588593, 588604, 588770, 588781, 588976, 588980, 589691, 589702, 589713, 589724, 589831, 589842, 589875, 589886, 594016, 594020, 594031, 594042, 594053, 594064, 594075, 594086, 594090, 594101, 594112, 594123, 594252, 594263, 594274, 594285, 594296, 594300, 594311, 594322, 594333, 594344, 594355, 594366, 594370, 594381, 594392, 594403, 594414, 594425, 594436, 594440, 594451, 594462, 594495, 594506, 594510, 594521, 594532, 594543, 594554, 594565, 594576, 594580, 594591, 594602, 594613, 594624, 594635, 594646, 594694, 594705, 594716, 594720, 594753, 594764, 594775, 594786, 594790, 594801, 594812, 594823, 594834, 594845, 594856, 594860, 594871, 594882, 594893, 594904, 594915, 594926, 594930, 594941, 598581, 682636, 682640, 682732, 682743, 687934, 687945, 698051, 698062, 698095, 698106, 698390, 698401, 698456, 698460, 698471, 698482, 698493, 698504, 698530, 698541, 745010, 745021, 745032, 745043, 745113, 745124, 745135, 745146, 745150, 745161 |
| Upper gastrointestinal tract disorder (gastroesophageal reflux disease or peptic ulcer disease) | | | | **ICD-9:** 041.86, 530.1, 530.2, 530.81, 530.85, 531, 532, 533, 534, 535, V12.71  **ICD-10:** B96.81, K20, K21, K22.1, K22.7, K25, K26, K27, K28, K29, Z87.11  **ATC:** A02BD04, A02BD11  **Medical procedure code:** 172616, 172620, 172631, 172642, 172653, 172664, 474854, 474865, 550093, 550104, 552370, 552381 |
| Lower gastrointestinal tract disorder (polyposis, diverticulosis, angiodysplasia, hemorrhoids) | | | | **ICD-9:** 211.3, 211.4, 448.0, 455, 537.82, 537.83, 537.84, 562, 569.84, 569.85, 569.86, V12.72  **ICD-10:** D12, I78.0, K31.81, K31.82, K55.2, K57, K63.5, K64, K63.81, Z86.010  **Medical procedure code:** 112313, 112324, 243294, 243305, 244311, 244322, 244355, 244366, 244370, 244381, 244554, 244565, 244576, 244580, 244591, 244602, 472150, 472161, 473211, 473222, 473476, 473480, 473675, 473686, 473955, 473966, 473970, 473981, 474795, 474806 |
| Diabetes mellitus | | | | **ICD-9:** 249, 250, 357.2, 362.0, 366.41, V45.85, V53.91, V65.46  **ICD-10:** E8, E9, E10, E11, E13, Z46.81, Z96.41  **ATC:** A10  **Medical procedure code:** 102852, 107015, 107030, 107052, 107074, 109594, 174370, 174381, 174392, 174403, 174414, 174425, 174436, 174440, 174451, 174462, 174473, 174484, 174495, 174506, 174510, 174521, 653671, 653682, 697093, 697104, 754176, 754191, 754250, 754272, 754736, 757352, 757374, 757396, 757411, 757514, 757536, 757551, 770070, 773393, 773496, 784630, 784641, 784652, 784663, 785735, 785750, 785772, 785794, 785816, 785831, 785853, 785875, 785890, 785912, 785934, 785956, 786015, 786030, 786100, 788756, 788771, 788793, 788815, 788830, 788852, 788874, 788896, 788911, 788933, 788955, 789751, 789773, 789795, 789810, 789832, 789854, 789876, 789891, 789913, 789935, 794032, 794113, 794135, 794150, 794194, 794216, 794231, 794253, 794275, 794290, 794312, 794334, 794356, 794371, 794393, 794415, 794430, 794452, 961295, 961306, 961332, 961343 |
| Thyroid disease  (hypo/hyperthyroidism, other/non-specific thyroid disease) | | | | **ICD-9:** 240-245, 246.1, 246.3, 246.8, 246.9, 648.1  **ICD-10:** E00-E06, E07.1, E07.89, E07.89, E07.9, E89.0, O09.5  **ATC:** H03AA, H03B, H03CA  **Medical procedure code:** 257014, 257025, 257036, 257040 |
| Anemia | | | | **ICD-9:** 280-285  **ICD-10:** D46.0-D46.4, D50-D53, D56-D64 |
| Osteoporosis | | | | **ICD-9:** 733.0  **ICD-10:** M80, M81  **ATC:** M05BA, M05BB, M05BX03, M05BX04, M05BX06, M05BX53, G03XC01, G03XC02, H05AA02 |
| Dementia  (Alzheimer’s disease, vascular, other) | | | | **ICD-9:** 046.11, 046.19, 290.0, 290.1, 290.2, 290.3, 290.4, 291.1, 294, 331.0, 331.11, 331.19, 331.82  **ICD-10:** A81.0, F01, F02, F03, F10.27, F10.97, G30, G31.0, G31.83, G31.85  **ATC:** N06D |
| Parkinson’s disease | | | | **ICD-9:** 331.6, 331.82, 332.0, 332.1, 333.0  **ICD-10:** G20, G21, G23.1, G31.83, G31.85, G90.3  **ATC:** N04AB, N04AC, N04B |
| History of falling | | | | **ICD-9:** E804, E833, E834, E835, E843, E880-E886, E888, E917.5-E917.9, E987, V15.88  **ICD-10:** R29.6, V00.141, V00.811, V00.831, V81.5, V81.6, V82.5, V82.6, V92.0, V93.3, V94.0, V97.0, W00, W01, W03, W05-W15, W16.0-W16.4, W17, W18, W19, Y21.1, Y21.3, Y30, Z91.81 |
| Prior thromboembolism | | | |  |
|  | | Stroke | | **ICD-9:** 431, 432.9, 433.01, 433.1, 433.21, 433.31, 433.81, 433.91, 434.01, 434.11, 434.91, 436, 438, V12.54  **ICD-10:** I61, I62.9, I63.0, I63.1, I63.2, I63.3, I63.4, I63.5, I63.8, I63.9, I67.89, I69.1, I69.2, I69.3, I69.8, I69.9, Z86.73  **Medical procedure code:** 182136, 182140, 182151, 182162, 182173, 182184, 477724, 477746, 477761, 477783 |
|  | | Systemic embolism | | **ICD-9:** 444, 557.0, 593.81  **ICD-10:** D73.5, I74, K55.01, K55.02, N28.0  **Medical procedure code:** 235130, 235141, 237112, 237123, 589175, 589186 |
| Prior major or clinically relevant non-major bleeding | | | | **ICD-9:** 285.1, 287.8, 287.9, 336.1, 360.43, 362.43, 362.81, 363.6, 363.72, 364.41, 372.72, 376.32, 377.42, 379.23, 388.69, 423.0, 430, 431, 432.0, 432.1, 432.9, 455.8, 456.0, 456.20, 459.0, 530.21, 530.7, 530.82, 531.00, 531.20, 531.40, 531.60, 532.00, 532.20, 532.40, 532.60, 533.00, 533.20, 533.40, 533.60, 534.00, 534.20, 534.40, 534.60, 535.01, 535.11, 535.21, 535.31, 535.41, 535.51, 535.61, 535.71, 537.83, 537.84, 562.02, 562.03, 562.12, 562.13, 568.81, 569.3, 569.85, 569.86, 578.0, 578.1, 578.9, 596.7, 599.7, 602.1, 620.7, 621.4, 623.6, 626.2, 626.5, 626.6, 626.7, 626.8, 626.9, 627.0, 627.1, 719.1, 729.92, 784.7, 784.8, 786.30, 786.39, 852.0, 852.2, 852.4, 853.0, 958.2  **ICD-10:** D62, D68.32, D69.8, D69.9, G95.19, H05.23, H11.3, H21.0, H31.3, H31.41, H35.6, H35.73, H43.1, H44.81, H47.02, H92.2x, I23.0, I31.2, I60, I61, I62.0, I62.1, I62.9, I85.01, I85.11, J94.2, J95.01, K22.11, K22.6, K22.8, K25.0, K25.2, K25.4, K25.6, K26.0, K26.2, K26.4, K26.6, K27.0, K27.2, K27.4, K27.6, K28.0, K28.2, K28.4, K28.6, K29.01, K29.21, K29.31, K29.41, K29.51, K29.61, K29.71, K29.81, K29.91, K31.811, K31.82, K50.011, K50.111, K50.811, K50.911, K51.011, K51.211, K51.311, K51.411, K51.511, K51.811, K51.911, K55.21, K57.01, K57.11, K57.13, K57.21, K57.31, K57.33, K57.41, K57.51, K57.53, K57.81, K57.91, K57.93, K62.5, K63.81, K64.9, K66.1, K92.0, K92.1, K92.2, K94.01, K94.11, K94.21, K94.31, M25.0, M79.81, N02, N30.01, N30.11, N30.21, N30.31, N30.41, N30.81, N30.91, N42.1, N83.6, N83.7, N85.7, N89.7, N92.0, N92.1, N92.3, N92.4, N93.0, N93.8, N93.9, N95.0, N99.510, N99.520, N99.530, R04.0, R04.1, R04.2, R04.89, R04.9, R31, R58, S06.340A, S06.341A, S06.342A, S06.343A, S06.344A, S06.345A, S06.346A, S06.347A, S06.348A, S06.349A, S06.350A, S06.351A, S06.352A, S06.353A, S06.354A, S06.355A, S06.356A, S06.357A, S06.358A, S06.359A, S06.360A, S06.361A, S06.362A, S06.363A, S06.364A, S06.365A, S06.366A, S06.367A, S06.368A, S06.369A, S06.4X0A, S06.4X1A, S06.4X2A, S06.4X3A, S06.4X4A, S06.4X5A, S06.4X6A, S06.4X7A, S06.4X8A, S06.4X9A, S06.5X0A, S06.5X1A, S06.5X2A, S06.5X3A, S06.5X4A, S06.5X5A, S06.5X6A, S06.5X7A, S06.5X8A, S06.5X9A, S06.6X0A, S06.6X1A, S06.6X2A, S06.6X3A, S06.6X4A, S06.6X5A, S06.6X6A, S06.6X7A, S06.6X8A, S06.6X9A, T79.2  **Medical procedure code:** 144605, 144620, 144642, 144664, 144686, 227441, 230403, 230425, 230440, 243600, 254940, 255242, 257445, 431620, 431944, 472124, 473686, 473782 |
| **PREVIOUS COMEDICATION USE (≤6 months before index date)^4^** | | | | |
| Antiplatelet | | | | **ATC:** B01AC56, B01AC06, C07FX02, C07FX03, C07FX04, C10BX01, C10BX02, C10BX04, C10BX05, C10BX06, C10BX08, C10BX12, B01AC04, B01AC05, B01AC22, B01AC24, B01AC25 |
|  | | Acetylsalicylic acid | | **ATC:** B01AC56, B01AC06, C07FX02, C07FX03, C07FX04, C10BX01, C10BX02, C10BX04, C10BX05, C10BX06, C10BX08, C10BX12 |
|  | | P2Y12-inhibitor | | **ATC:** B01AC04, B01AC05, B01AC22 , B01AC24 , B01AC25 |
| Rate control therapy | | | | **ATC:** C07AB, C07AG, C07BB, C07BG, C07CB, C07CG, C07DB, C07FB, C07FX03, C07FX04, C07FX05, C07FX06, C09BX02, C09BX04, C09BX05, C09DX05, C08D, C01AA05, C01AA08 |
|  | Cardioselective beta-blockers | | | **ATC:** C07AB, C07AG, C07BB, C07BG, C07CB, C07CG, C07DB, C07FB, C07FX03, C07FX04, C07FX05, C07FX06, C09BX02, C09BX04, C09BX05, C09DX05 |
|  | Verapamil, diltiazem | | | **ATC:** C08D |
|  | | Digoxin | | **ATC:** C01AA05, C01AA08 |
| Rhythm control therapy | | | | **ATC:** C01BA, C01BB, C01BC, C01BD, C07AA07, C07BA07, C07FX02 |
|  | | Class I AAD | | **ATC:** C01BA, C01BB, C01BC |
|  | | Class III AAD | | **ATC:** C01BD, C07AA07, C07BA07, C07FX02 |
| ACE-inhibitor/ARB | | | | **ATC:** C09A, C09B, C09C, C09D, C10BX04, C10BX06, C10BX07, C10BX10, C10BX11, C10BX12, C10BX13, C10BX14, C10BX15, C10BX16, C10BX17, C10BX18 |
| DHP calcium channel blocker | | | | **ATC:** C07FB, C08C, C08G, C09BB, C09DB, C09BX01, C09BX03, C09BX04, C09DX01, C09DX03, C09DX06, C09DX07, C09XA53, C09XA54, C10BX03, C10BX07, C10BX09, C10BX11, C10BX14, C10BX18 |
| Loop diuretic | | | | **ATC:** C03C, C03EB |
| Non-loop diuretic | | | | **ATC:** C02L, C03A, C03BA, C03BB, C03EA, C07B, C07C, C07D, C08GA, C09BA, C09BX01, C09BX03, C09DA, C09DX01, C09DX03, C09DX06, C09DX07, C09XA52, C09XA54, C10BX13, C03D, C03EA, C03EB |
| Proton pump inhibitor | | | | **ATC:** A02BC |
| NSAID | | | | **ATC:** M01AA, M01AB, M01AC, M01AE, M01AG, M01AH, N02AJ08, N02AJ14, N02AJ19, C08CA51 |
| Oral corticosteroid | | | | **ATC:** H02AB, H02BX01 |
| SSRI/SNRI | | | | **ATC**: N06AB, N06AX16, N06AX21, N06CA03 |
| **PRESCRIBING PHYSICIAN’S SPECIALTY (on index date)** *(based on last three digits of the physician’s RIZIV/INAMI number^5^)* | | | | |
| Primary care physician | | | | **RIZIV/INAMI number:** 001, 002, 003, 004, 005, 006, 007, 008 |
| Cardiologist | | | | **RIZIV/INAMI number:** 073, 131, 591, 595, 631, 730, 731, 733, 734, 735, 736, 737, 738, 739, 989 |
| Other | | | | **RIZIV/INAMI number:** 000, 009, 010, 011, 014, 017, 018, 021, 034, 037, 040, 041, 044, 045, 048, 052, 055, 058, 062, 065, 066, 068, 069, 073, 076, 077, 078, 079, 080, 083, 084, 086, 087, 090, 093, 096, 097, 100, 109, 119, 140, 149, 170, 173, 180, 182, 184, 192, 200, 201, 210, 300, 301, 340, 370, 373, 374, 400, 404, 410, 414, 418, 422, 440, 450, 458, 473, 480, 489, 494, 496, 500, 518, 520, 550, 570, 573, 580, 581, 583, 584, 586, 589, 593, 597, 598, 600, 603, 620, 623, 624, 628, 650, 653, 659, 660, 673, 690, 694, 696, 698, 700, 714, 760, 764, 770, 774, 779, 780, 784, 790, 794, 795, 800, 830, 834, 860, 862, 867, 870, 873, 900, 930, 939, 960, 970, 973, 983, 985, 994, 995, 996, 999 |
| **CLINICAL RISK SCORES** | | | | |
| CHA_2_DS_2_-VASc score^6^ | | | | - **Congestive heart failure:** 1 point (definition mentioned above: ‘Congestive heart failure’)  - **Hypertension**: 1 point (definition mentioned above: ‘Hypertension’)  - **Age ≥75 years**: 2 point  - **Diabetes mellitus**: 1 point (definition mentioned above: ‘Diabetes mellitus’)  - **Stroke or systemic embolism**: 2 point (definition mentioned above: ‘Thromboembolism (stroke and/or systemic embolism)’)  - **Vascular disease**: 1 point (definition mentioned above: ‘Coronary artery disease’ & ‘Peripheral artery disease’)  - **Age 65-74 years**: 1 point  - **Sex category (female)**: 1 point |
| HAS-BLED score^6^ | | | | - **Hypertension**: 1 point (definition mentioned above: ‘Hypertension’)  - **Abnormal renal function**: 1 point (definition mentioned above: ‘Chronic kidney disease’)  - **Abnormal liver function**: 1 point (definition mentioned above: ‘Chronic liver disease’)  - **Stroke**: 1 point (definition mentioned above: ‘Stroke’)  - **Bleeding history or predisposition**: 1 point (definition mentioned above: ‘Prior major or clinically relevant non-major bleeding’)  - **Labile** INR: not available  - **Elderly (>65 years or frailty):** 1 point  - **Drugs (antiplatelet, NSAID):** 1 point (definition mentioned above: ‘Antiplatelet’ & ‘NSAID’)  - **Excessive alcohol drinking**: 1 point:   - **ICD-9:** 265.1, 291, 303, 305.0, 357.5, 425.5, 535.3, 571.0-571.3, 980.0, E860.1, V11.3 - **ICD-10:** E51, F10, G31.2, G62.1, G72.1, I42.6, K29.2, K70, K85.2, K86, O35.4, T51.0, T51.9, Z71.4 - **ATC:** N07BB - **Medical procedure code:** 790090 |
| Charlson Comorbidity Index^7,8^ | | | | - **Myocardial infarction:** 1 point:   - **ICD-9:** 410.00, 410.01, 410.10, 410.11, 410.20, 410.21, 410.30, 410.31, 410.40, 410.41, 410.50, 410.51, 410.60, 410.61, 410.70, 410.71, 410.80, 410.81, 410.90, 410.91 - **ICD-10:** I21, I22   - **Congestive heart failure**: 1 point (definition mentioned above: ‘Congestive heart failure’)  - **Peripheral vascular disease**: 1 point (definition mentioned above: ‘Peripheral artery disease’)  - **Cerebrovascular disease:** 1 point   - **ICD-9:** 362.34, 430, 431, 432, 433, 434, 435, 436, 437, 438 - **ICD-10:** I61, I62, I63, I65, I66, I67, I68, I69, G45, G46, H34.0   - **Dementia**: 1 point (definition mentioned above: ‘Dementia’)  - **Chronic pulmonary disease**: 1 point (definition mentioned above: ‘Chronic lung disease’)  - **Connective tissue disease**: 1 point:   - **ICD-9:** 136.1, 287.0, 446, 447.5, 447.6, 710, 711.2, 725, 728.5, 729.30 - **ICD-10:** D69.0, M30, M31, M32, M33, M34, M35, M36.0, M36.8   - **Peptic ulcer disease**: 1 point:   - **ICD-9:** 041.86, 530.2, 531, 532, 533, 534, V12.71 - **ICD-10:** B96.81, K22.1, K25, K26, K27, K28, Z87.11 - **ATC:** A02BD04, A02BD11 - **Medical procedure code:** 550093, 550104, 552370, 552381   - **Mild liver disease**: 1 point   - **ICD-9:** 070.3, 070.51, 070.52, 070.54, 070.70, 571, 572.8, 573.0, 573.8, 573.9 - **ICD-10:** B17.0, B17.10, B18, B19.10, B19.20, K70.0, K70.1, K70.2, K70.3, K70.9, K71.3, K71.4, K71.5, K71.6, K71.7, K71.8, K71.9, K73, K74, K75.3, K75.4, K75.8, K75.9, K76.0, K76.1, K76.2, K76.89, K76.9, K77 - **ATC:** J05AB04, J05AF05, J05AF07, J05AF08, J05AF10, J05AE11, J05AE12, J05AE14, J05AX15, J05AX65, J05AP - **Medical procedure code:** 556754, 556765   - **Diabetes without chronic complications**: 1 point   - **ICD-9:** 249.4-249.7, 249.9, 250.4-250.7, 250.9, 357.2, 362.0, 364.41 - **ICD-10:** E08.2-E08.5, E08.8, E09.2-E09.5, E09.8, E10.2-E10.5, E10.8, E11.2-E11.5, E11.8, E13.2-E13.5, E13.8 - **Medical procedure code:** 653671, 653682, 697093, 697104, 770070, 773393, 773496   - **Diabetes with chronic complications**: 2 points   - **ICD-9:** 249.0-249.3, 249.8, 250.0-250.3, 250.8 - **ICD-10:** E08.0, E08.1, E08.6, E08.9, E09.0, E09.1, E09.6, E09.9, E10.1, E10.6, E10.9, E11.0, E11.1, E11.6, E11.9, E13.0, E13.1, E13.6, E13.9   - **Hemiplegia or paraplegia**: 2 points:   - **ICD-9:** 334.1, 342, 343.0, 343.1, 343.2, 343.4, 344.0, 344.1, 344.2, 344.9, 438.2 - **ICD-10:** G04.1, G11.4, G80.0, G80.1, G80.2, G81, G82, G83.0, G86.9, I69.05, I69.15, I69.25, I69.35, I69.85, I69.95 - **Medical procedure code:** 643414, 643425   - **Renal disease:** 2 points   - **ICD-9:** 403.01, 403.11, 403.91, 404.02, 404.12, 404.92, 585.5, 585.6, 586, 996.81, V42.0, V45.11, V56 - **ICD-10:** N18.5, N18.6, N19, I12.0, I13.11, T86.1, Z49, Z94.0, Z99.2 - **Medical procedure code:** N81 (group code), 318010, 318021, 318290, 318301   - **Any malignancy, including leukemia and lymphoma:** 2 points:   - **ICD-9:** 140-195, 199.1, 199.2, 200-209, 223, 230-239, 258.0, V58.0, V58.11, V58.12 - **ICD-10:** C00-C76, C80.1, C80.2, C81-C96, D00-D09, D37-D49, E31.2, Z51.0, Z51.11, Z51.12 - **ATC:** L01 - **Medical procedure code:** 154873, 154884, 154895, 154906, 157231, 157242, 201191, 201202, 201213, 201224, 220275, 220286, 220371, 220382, 201213, 201224, 226914, 226925, 226936, 226940, 227216, 227220, 227275, 227286, 227636, 227640, 227651, 227662, 227673, 227684, 227695, 227706, 227710, 227721, 227732, 227743, 227754, 227765, 227776, 227780, 227791, 227802, 227813, 227824, 227835, 227846, 228012, 228023, 228174, 228185, 228233, 228244, 228255, 228266, 228270, 228281, 228292, 228303, 228314, 228325, 228336, 228340, 230473, 230484, 231033, 231044, 241231, 241242, 241415, 241426, 241430, 241441, 241452, 241463, 241555, 241566, 242012, 242023, 242034, 242045, 242292, 242303, 242314, 242325, 242830, 242841, 242852, 242863, 242874, 242885, 242896, 242900, 243051, 243062, 243073, 243084, 243235, 243246, 243736, 243740, 243751, 243762, 243773, 243784, 244016, 244020, 244031, 244042, 244075, 244086, 244790, 244801, 244856, 244860, 244893, 244904, 244915, 244926, 244930, 244941, 244952, 244963, 244974, 244985, 245512, 245523, 245534, 245545, 246050, 246061, 246072, 246083, 247111, 247122, 247133, 247144, 251753, 251764, 251775, 251786, 254892, 254903, 256115, 256126, 256336, 256340, 256572, 256583, 257191, 257202, 258355, 258366, 258370, 258381, 258392, 258403, 258451, 258462, 258554, 258565, 258856, 258860, 258871, 258882, 258893, 258904, 259033, 259044, 259114, 259125, 260190, 260201, 260411, 260422, 260433, 260444, 260551, 260562, 260654, 260665, 260750, 260761, 261111, 261122, 261391, 261402, 261472, 261483, 261671, 261682, 261774, 261785, 261796, 261800, 262334, 262345, 262570, 262581, 277756, 277760, 277771, 277782, 278795, 278806, 278810, 278821, 281831, 281842, 281956, 281960, 282310, 282321, 282671, 282682, 284056, 284060, 288455, 288466, 288470, 288481, 289892, 289903, 291056, 291060, 310494, 310505, 311312, 311323, 312550, 312561, 312572, 312583, 312594, 312605, 312653, 312664, 312970, 312981, 350114, 350125, 350136, 350140, 350276, 350280, 350291, 350302, 350372, 350383, 350674, 350685, 350696, 350700, 431174, 431185, 431336, 431340, 431351, 431362, 432294, 432305, 444113, 444124, 444135, 444146, 444150, 444161, 444172, 444183, 444194, 444205, 444216, 444220, 444231, 444242, 444253, 444264, 444275, 444286, 444290, 444301, 444312, 444323, 444334, 444345, 444474, 444485, 444592, 444603, 473970, 473981, 474795, 474806, 565073, 565084, 565095, 565106, 565110, 565121, 565132, 565143, 565154, 565165, 587834, 587845, 587871, 587882, 587893, 587904, 587915, 587926, 588431, 588442, 588453, 588464, 588475, 588486, 588490, 588501, 588512, 588523, 588534, 588545, 588556, 588560, 588571, 588582, 588593, 588604, 588770, 588781, 588976, 588980, 589691, 589702, 589713, 589724, 589831, 589842, 589875, 589886, 594016, 594020, 594031, 594042, 594053, 594064, 594075, 594086, 594090, 594101, 594112, 594123, 594252, 594263, 594274, 594285, 594296, 594300, 594311, 594322, 594333, 594344, 594355, 594366, 594370, 594381, 594392, 594403, 594414, 594425, 594436, 594440, 594451, 594462, 594495, 594506, 594510, 594521, 594532, 594543, 594554, 594565, 594576, 594580, 594591, 594602, 594613, 594624, 594635, 594646, 594694, 594705, 594716, 594720, 594753, 594764, 594775, 594786, 594790, 594801, 594812, 594823, 594834, 594845, 594856, 594860, 594871, 594882, 594893, 594904, 594915, 594926, 594930, 594941, 598581, 682636, 682640, 682732, 682743, 687934, 687945, 698051, 698062, 698095, 698106, 698390, 698401, 698456, 698460, 698471, 698482, 698493, 698504, 698530, 698541, 745010, 745021, 745032, 745043, 745113, 745124, 745135, 745146, 745150, 745161   - **Moderate or severe liver disease:** 3 points   - **ICD-9:** 070.0, 070.2, 070.4, 070.6, 070.71, 155.0, 155.1, 155.2, 197.7, 456.0, 456.1, 456.2, 567.23, 570, 571.2, 571.5, 571.6, 572.2, 572.3, 572.4, 573.0, 573.5, 789.59, V42.7 - **ICD-10:** B15.0, B16.0, B16.2, B17.11, B19.0, B19.11, B19.21, C22, C78.7, I85, I86.4, K65.2, K70.2, K70.3, K70.4, K71.1, K71.7, K72, K74, K76.1, K76.5, K76.6, K76.7, K76.81, R18.8, Z94.4 - **Medical procedure code:** 318076, 318080, 318334, 318345, 472113, 472124, 589352, 589363   - **Metastatic solid tumor:** 6 points   - **ICD-9:** 196-198, 199.0 - **ICD-10:** C77-C79, C80.0   - **AIDS/HIV:** 6 points   - **ICD-9:** 042, V08 - **ICD-10:** B20, Z21 - **ATC:** J05AE01, J05AE02, J05AE03, J05AE04, J05AE05, J05AE07, J05AE08, J05AE09, J05AE10, J05AF01, J05AF02, J05AF03, J05AF04, J05AF05, J05AF06, J05AF09, J05AF11, J05AF12, J05AF13, J05AG, J05AR, J05AX07, J05AX08, J05AX09, J05AX12   - **Age:**   - <50 years: 0 points - 50-59 years: 1 point - 60-69 years: 2 points - 70-79 years: 3 points - ≥80 years: 4 points   *The following comorbid conditions were mutually exclusive: diabetes with chronic complications and diabetes without chronic complications; mild liver disease and moderate or severe liver disease; and any malignancy and metastatic solid tumor*. |
| John Hopkins Claims-based Frailty Indicator^9^ | | | | - **Impaired mobility**: beta coefficient 1.24:   - **ICD-9:** 334, 719.7, 781.2, V46.3, V49.84, V57.81 - **ICD-10:** G11, G32.81, M62.3, R26, R29.6, Z74.01, Z74.09, Z99.3 - **Medical procedure code:** N83, 643451, 643462, 653656, 653660, 770394, 770405, 770416, 770420   - **Depression**: beta coefficient 0.54:   - **ICD-9**: 293.83, 296, 300.4, 301.12, 309.0, 309.1, 309.28, 311 - **ICD-10**: F06.31, F06.32, F30, F31, F32, F33, F34.1, F43.21, F43.23 - **ATC:** N06A   - **Congestive heart failure**: beta coefficient 0.50: (definition mentioned above: ‘Congestive heart failure’)  - **Parkinson’s disease**: beta coefficient 0.50: (definition mentioned above: ‘Parkinson’s disease’)  - **White race**: beta coefficient -0.49: not available  - **Arthritis (any type):** beta coefficient 0.43:   - **ICD-9:** 099.3, 696.0, 711.1, 711.3, 713.1, 714.0, 714.1, 714.2, 714.3, 714.4, 714.8, 714.9, 716.5, 716.6, 720.0, 720.2, 720.89, 720.9, V13.4 - **ICD-10:** L40.5, M02.1, M02.3, M05, M06, M07, M08, M13.0, M13.1, M45, M46.1, M46.8, M46.9, Z87.39 - **ATC:** L04AA13, L04AA24, L04AA29, L04AA37 - **Medical procedure code:** 478030, 478041   - **Cognitive impairment**: beta coefficient 0.33:   - **ICD-9:** 331.2, 331.83, 331.89, 331.9, 797 - **ICD-10:** G31.1, G31.84, G31.89, G31.9, R41.81   - **Charlson comorbidity index** **(> 0)**: beta coefficient 0.31  - **Stroke**: beta coefficient 0.28: (definition mentioned above: ‘Stroke’)  - **Paranoia**: beta coefficient 0.24:   - **ICD-9:** 293.81, 293.82, 295, 297, 298 - **ICD-10:** F06.0, F06.2, F20, F22, F23, F24, F28, F29   - **Chronic skin ulcer**: beta coefficient 0.23:   - **ICD-9:** 707 - **ICD-10:** E08.621, E08.622, E09.621, E09.622, E10.621, E10.622, E11.621, E11.622, E13.621, E13.622, L89, L97, L98.4 - **Medical procedure code:** 114074, 114085   - **Pneumonia**: beta coefficient 0.21:   - **ICD-9:** 480, 481, 482, 483, 484, 485, 486, 487.0 - **ICD-10:** A48.1, J11.0, J12, J13, J14, J15, J16, J17, J18   - **Male sex**: beta coefficient -0.19  - **Skin and soft tissue infection**: beta coefficient 0.18:   - **ICD-9:** 680, 681, 682, 683, 684, 685, 686, 695.81 - **ICD-10:** L00, L01, L02, L03, L04, L05, L08   - **Mycoses**: beta coefficient 0.14:   - **ICD-9:** 110, 111, 112, 114, 115, 116, 117, 118 - **ICD-10:** B35, B36, B37, B38, B39, B40, B41, B42, B43, B44, B45, B46, B47, B48, B49   - **Age (in 5 year categories)**: beta coefficient 0.09  - **Admission in past 6 months**: beta coefficient 0.09  - **Gout or other crystal-induced arthropathy**: beta coefficient 0.08:   - **ICD-9:** 274, 712.3, 712.8, 712.90 - **ICD-10:** M10, M11, M1A - **ATC:** M04A   - **Falls**: beta coefficient 0.08: (definition mentioned above: ‘History of falling’)  - **Musculoskeletal problems**: beta coefficient 0.05:   - **ICD-9:** 713, 716.0, 716.2, 716.3, 716.4, 716.5, 716.6, 716.8, 716.9, 718.1, 718.2, 718.5, 718.6, 718.7, 718.8, 718.9, 719-724, 733.0, 733.1, 733.93, 733.94, 733.95, 733.96, 733.97, 733.98, V13.51, V13.52 - **ICD-10:** M07, M12.0, M12.1, M12.2, M12.3, M12.4, M12.8, M12.9, M13, M14, M24.0, M24.3, M24.6, M24.7, M24.8, M24.9, M25, M45, M46.0, M46.1, M46.4, M46.8, M46.9, M47-M51, M53, M54, M80, M81, M84.3, M84.4, M84.5, M84.6, Z87.31   - **Urinary tract infection**: beta coefficient 0.05:   - **ICD-9:** 590.1, 590.8, 590.9, 595.0, 595.4, 595.89, 595.9, 597, 599.0 - **ICD-10:** N30.0, N30.8, N30.9, N10, N12, N13.6, N15.9, N16, N34, N39.0 - **ATC:** J01XE01, J01XX01 |
| **LABORATORY TESTING** | | | | |
| INR measurement | | | | **Medical procedure code:** 554573, 554584 |

**eTable 2:** Definition of in- and exclusion criteria, comorbidities, comedication, prescribing physician’s specialty and clinical risk scores based on ICD-coded hospital discharge diagnoses (ICD-9-CM up to 2014 and ICD-10-BE from 2015 onward)^2^, medical procedure codes^3^, ATC-coded prescription claims^4^ and/or the last three digits of the physician’s identification code of the Belgian National Institute for Health and Disability Insurance (RIZIV/INAMI)^5^, respectively.

AAD: antiarrhythmic drug; ACE inhibitor: Angiotensin-converting enzyme inhibitor; ARB: Angiotensin II receptor blocker; AF: Atrial fibrillation; ATC: Anatomical Therapeutic Chemical Classification; CKD: Chronic kidney disease; DHP: dihydropyridine; ICD-9-CM: International Classification of Diseases (ICD) codes, 9^th^ revision, Clinical Modification; ICD-10-BE: International Classification of Diseases (ICD) codes, 10^th^ Revision, Belgian Modification; INR: International Normalized Ratio; NOAC: non-vitamin K antagonist oral anticoagulant; NSAID: non-steroidal anti-inflammatory drug; OAC: oral anticoagulant; RIZIV/INAMI: Rijksinstituut voor ziekte- en invaliditeitsverzekering/Institut national d'assurance maladie-invalidité; VKA: vitamin K antagonist; y: year.

## eTable 3: Persistence, reinitiation, switching and adherence rates over time

| **Time interval** | **PERSISTENCE** | | | | | |
| --- | --- | --- | --- | --- | --- | --- |
|  | **VKA** | **NOAC** | **Dabigatran** | **Rivaroxaban** | **Apixaban** | **Edoxaban** |
| **3 months** | 56.5% [56.1-57.0] | 87.4% [87.3-87.6] | 84.2% [83.8-84.6] | 89.0% [88.7-89.2] | 85.4% [85.1-85.6] | 91.9% [91.6-92.1] |
| **6 months** | 46.5% [46.1-46.9] | 79.8% [79.7-80.0] | 77.8% [77.3-78.3] | 78.6% [78.3-78.9] | 80.0% [79.7-80.2] | 84.6% [84.2-85.0] |
| **9 months** | 40.8% [40.4-41.3] | 74.7% [74.5-74.9] | 73.1% [72.6-73.6] | 72.6% [72.2-72.9] | 75.2% [74.8-75.5] | 80.3% [79.9-80.8] |
| **1 year** | 37.2% [36.8-37.7] | 69.6% [69.4-69.8] | 68.1% [67.5-68.7] | 67.1% [66.8-67.5] | 69.8% [69.5-70.2] | 76.9% [76.4-77.4] |
| **2 years** | 29.4% [28.9-29.8] | 59.4% [59.2-59.6] | 58.3% [57.7-59.0] | 56.8% [56.4-57.2] | 59.7% [59.3-60.1] | 67.8% [67.1-68.4] |
| **3 years** | 24.8% [24.4-25.3] | 53.2% [52.9-53.4] | 52.1% [51.4-52.8] | 50.8% [50.4-51.2] | 53.8% [53.3-54.2] | 63.1% [62.2-64.0] |
| **4 years** | 21.7% [21.3-22.1] | 48.4% [48.1-48.6] | 47.6% [46.9-48.3] | 46.3% [45.8-46.7] | 48.9% [48.4-49.4] | NA |
| **5 years** | 18.9% [18.4-19.3] | 44.3% [44.0-44.6] | 43.7% [43.0-44.5] | 42.3% [41.8-42.8] | 45.2% [44.6-45.8] | NA |
| **Time interval** | **SWITCHING** | | | | | |
|  | **VKA** | **NOAC** | **Dabigatran** | **Rivaroxaban** | **Apixaban** | **Edoxaban** |
| **3 months** | 9.0% [8.8-9.3] | 1.3% [1.2-1.3] | 5.9% [5.7-6.2] | 3.5% [3.4-3.7] | 2.6% [2.5-2.7] | 2.8% [2.6-3.0] |
| **6 months** | 13.1% [12.7-13.4] | 1.8% [1.7-1.9] | 8.4% [8.0-8.7] | 5.1% [4.9-5.3] | 3.4% [3.3-3.6] | 4.1% [3.9-4.3] |
| **9 months** | 15.6% [15.2-16.0] | 2.2% [2.1-2.2] | 10.1% [9.7-10.5] | 6.2% [6.0-6.4] | 4.1% [3.9-4.2] | 4.9% [4.7-5.2] |
| **1 year** | 17.6% [17.2-18.1] | 2.5% [2.4-2.5] | 11.8% [11.4-12.2] | 7.2% [6.9-7.4] | 4.6% [4.4-4.8] | 5.6% [5.3-5.9] |
| **2 years** | 24.8% [24.2-25.4] | 3.4% [3.3-3.5] | 15.8% [15.3-16.3] | 10.3% [10.0-10.6] | 6.1% [5.9-6.3] | 7.5% [7.1-7.9] |
| **3 years** | 31.6% [30.9-32.3] | 4.0% [3.9-4.1] | 19.2% [18.6-19.8] | 13.1% [12.8-13.4] | 7.4% [7.1-7.6] | 9.2% [8.5-9.8] |
| **4 years** | 38.5% [37.6-39.3] | 4.6% [4.4-4.7] | 22.2% [21.5-22.8] | 15.8% [15.4-16.2] | 8.7% [8.3-9.0] | NA |
| **5 years** | 44.2% [43.2-45.1] | 5.1% [4.9-5.2] | 25.4% [24.6-26.2] | 18.0% [17.5-18.5] | 9.6% [9.2-10.0] | NA |
| **Time interval** | **REINITIATION** | | | | | |
|  | **VKA** | **NOAC** | **Dabigatran** | **Rivaroxaban** | **Apixaban** | **Edoxaban** |
| **3 months** | 13.6% [13.3-14.0] | 25.6% [25.3-25.9] | 28.2% [27.5-29.0] | 21.5% [21.1-21.9] | 31.2% [30.7-31.7] | 21.8% [20.9-22.7] |
| **6 months** | 22.9% [22.5-23.4] | 48.7% [48.4-49.0] | 53.2% [52.4-54.1] | 42.7% [42.2-43.2] | 56.0% [55.5-56.6] | 43.5% [42.4-44.6] |
| **9 months** | 26.4% [26.0-26.9] | 54.9% [54.5-55.2] | 59.4% [58.5-60.2] | 48.9% [48.3-49.4] | 62.2% [61.6-62.7] | 49.7% [48.6-50.9] |
| **1 year** | 28.9% [28.5-29.4] | 58.1% [57.7-58.4] | 62.4% [61.6-63.3] | 52.2% [51.7-52.8] | 65.1% [64.5-65.7] | 53.4% [52.2-54.5] |
| **2 years** | 35.0% [34.5-35.5] | 64.8% [64.4-65.1] | 68.9% [68.0-69.7] | 59.5% [59.0-60.1] | 70.8% [70.2-71.4] | 61.2% [59.8-62.6] |
| **3 years** | 39.3% [38.8-39.8] | 69.0% [68.6-69.4] | 73.3% [72.4-74.1] | 64.3% [63.8-64.9] | 74.2% [73.6-74.8] | 66.4% [63.7-69.0] |
| **4 years** | 42.9% [42.3-43.5] | 72.4% [72.0-72.8] | 76.5% [75.6-77.3] | 68.2% [67.6-68.8] | 77.1% [76.4-77.8] | NA |
| **5 years** | 46.1% [45.5-46.7] | 75.4% [74.9-75.8] | 78.9% [77.9-79.8] | 71.4% [70.8-72.1] | 80.2% [79.3-81.0] | NA |
| **Time interval** | **ADHERENCE (PDC ≥90%)** | | | | | |
|  | **VKA** | **NOAC** | **Dabigatran** | **Rivaroxaban** | **Apixaban** | **Edoxaban** |
| **3 months** | NA | 168 831 (85.8%) | 21 859 (85.1%) | 61 358 (87.7%) | 58 326 (82.4%) | 27 288 (89.4%) |
| **6 months** | NA | 143 633 (89.0%) | 19 161 (87.4%) | 51 330 (90.7%) | 51 082 (86.2%) | 22 060 (92.8%) |
| **9 months** | NA | 124 606 (90.4%) | 17 025 (89.1%) | 45 019 (92.0%) | 44 521 (88.0%) | 18 041 (93.7%) |
| **1 year** | NA | 107 571 (90.2%) | 15 021 (88.6%) | 39 564 (91.3%) | 38 330 (87.8%) | 14 656 (94.7%) |
| **2 years** | NA | 67 746 (94.7%) | 10 405 (94.3%) | 27 428 (96.4%) | 24 422 (92.5%) | 5491 (97.7%) |
| **3 years** | NA | 42 923 (96.6%) | 7331 (96.5%) | 19 757 (97.8%) | 15 301 (95.2%) | 534 (98.7%) |
| **4 years** | NA | 25 786 (97.8%) | 4720 (97.4%) | 12 819 (98.6%) | 8247 (96.7%) | NA |
| **5 years** | NA | 13 842 (98.4%) | 2853 (98.1%) | 7623 (99.0%) | 3366 (97.3%) | NA |

**eTable 3:** Kaplan-Meier estimates with 95% confidence interval of persistence, switching and reinitiation of VKAs, NOACs and individual NOAC types (dabigatran, rivaroxaban, apixaban and edoxaban). Moreover, the number and proportion of adherent NOAC (type) users (PDC ≥90%) over time are shown.

NA: not available; NOAC: non-vitamin K antagonist oral anticoagulant; PDC: proportion of days covered; VKA: vitamin K antagonist.

## eTable 4: Baseline characteristics (subgroup of subjects with ≥1 year of follow-up)

| **Patient characteristics** | | **VKA**  **(n = 47 914)** | **NOAC** | | | | |
| --- | --- | --- | --- | --- | --- | --- | --- |
|  |  |  | **Overall**  **(n = 166 226)** | **Dabigatran**  **(n = 24 795)** | **Rivaroxaban**  **(n = 63 227)** | **Apixaban**  **(n = 57 803)** | **Edoxaban**  **(n = 20 401)** |
|  | Age (years) | 72.2 ± 11.1 | 76.6 ± 9.2 | 76.3 ± 9.0 | 76.3 ± 9.2 | 77.2 ± 9.1 | 76.3 ± 9.3 |
|  | <65 years | 12 720 (26.5%) | 15 327 ( 9.2%) | 2401 ( 9.7%) | 6088 ( 9.6%) | 4790 ( 8.3%) | 2048 (10.0%) |
|  | 65-74 years | 14 188 (29.6%) | 53 175 (32.0%) | 8010 (32.3%) | 20 649 (32.7%) | 17 611 (30.5%) | 6905 (33.8%) |
|  | 75-84 years | 14 860 (31.0%) | 65 991 (39.7%) | 10 045 (40.5%) | 25 263 (40.0%) | 23 103 (40.0%) | 7580 (37.2%) |
|  | ≥85 years | 6146 (12.8%) | 31 733 (19.1%) | 4339 (17.5%) | 11 227 (17.8%) | 12 299 (21.3%) | 3868 (19.0%) |
|  | Female | 22 484 (46.9%) | 79 700 (47.9%) | 11 628 (46.9%) | 30 149 (47.7%) | 28 479 (49.3%) | 9444 (46.3%) |
|  | Reduced dose | NA | 59 102 (35.6%) | 13 553 (54.7%) | 24 180 (38.2%) | 15 593 (27.0%) | 5776 (28.3%) |
|  | Follow-up (years) | 4.3 ± 1.7 | 3.4 ± 1.7 | 4.0 ± 1.8 | 3.9 ± 1.7 | 3.2 ± 1.4 | 1.9 ± 0.6 |
| **Comorbidities** | | | | | | | |
|  | Hypertension | 32 768 (68.4%) | 114 418 (68.8%) | 16 766 (67.6%) | 42 874 (67.8%) | 40 963 (70.9%) | 13 815 (67.7%) |
|  | Coronary artery disease | 12 313 (25.7%) | 28 876 (17.4%) | 3807 (15.4%) | 10 522 (16.6%) | 10 913 (18.9%) | 3634 (17.8%) |
|  | Congestive heart failure | 7822 (16.3%) | 24 022 (14.5%) | 2931 (11.8%) | 8732 (13.8%) | 9475 (16.4%) | 2885 (14.1%) |
|  | Valvular heart disease | 10 185 (21.3%) | 19 744 (11.9%) | 2624 (10.6%) | 6632 (10.5%) | 7756 (13.4%) | 2732 (13.4%) |
|  | Peripheral artery disease | 5833 (12.2%) | 11 541 (6.9%) | 1579 (6.4%) | 3968 (6.3%) | 4692 (8.1%) | 1302 (6.4%) |
|  | Dyslipidemia | 29 261 (61.1%) | 98 172 (59.1%) | 14 856 (59.9%) | 36 148 (57.2%) | 35 078 (60.7%) | 12 090 (59.3%) |
|  | Chronic kidney disease | 6693 (14.0%) | 16 410 (9.9%) | 1499 (6.0%) | 5457 (8.6%) | 7206 (12.5%) | 2247 (11.0%) |
|  | Chronic liver disease | 1936 (4.0%) | 4390 (2.6%) | 554 (2.2%) | 1641 (2.6%) | 1649 (2.9%) | 546 (2.7%) |
|  | Chronic lung disease | 6647 (13.9%) | 18 663 (11.2%) | 2446 (9.9%) | 7020 (11.1%) | 6962 (12.0%) | 2234 (11.0%) |
|  | Obstructive sleep apnea | 1893 (4.0%) | 5664 (3.4%) | 793 (3.2%) | 2112 (3.3%) | 1998 (3.5%) | 761 (3.7%) |
|  | Cancer | 4317 (9.0%) | 14 555 (8.8%) | 1943 (7.8%) | 5489 (8.7%) | 5282 (9.1%) | 1842 (9.0%) |
|  | Upper GI tract disorder | 4055 (8.5%) | 11 399 (6.9%) | 1434 (5.8%) | 4381 (6.9%) | 4401 (7.6%) | 1183 (5.8%) |
|  | Lower GI tract disorder | 3312 (6.9%) | 11 450 (6.9%) | 1527 (6.2%) | 4304 (6.8%) | 4195 (7.3%) | 1425 (7.0%) |
|  | Diabetes mellitus | 19 731 (41.2%) | 51 663 (31.1%) | 6983 (28.2%) | 18 945 (30.0%) | 19 391 (33.5%) | 6344 (31.1%) |
|  | Thyroid disease | 7108 (14.8%) | 23 882 (14.4%) | 3388 (13.7%) | 9180 (14.5%) | 8681 (15.0%) | 2632 (12.9%) |
|  | Anemia | 5066 (10.6%) | 10 910 (6.6%) | 1238 (5.0%) | 3976 (6.3%) | 4428 (7.7%) | 1267 (6.2%) |
|  | Osteoporosis | 3076 (6.4%) | 11 425 (6.9%) | 1679 (6.8%) | 4430 (7.0%) | 4109 (7.1%) | 1207 (5.9%) |
|  | Dementia | 1945 (4.1%) | 7743 (4.7%) | 974 (3.9%) | 2913 (4.6%) | 3041 (5.3%) | 815 (4.0%) |
|  | Parkinson’s disease | 1262 (2.6%) | 4768 (2.9%) | 705 (2.8%) | 1706 (2.7%) | 1810 (3.1%) | 547 (2.7%) |
|  | History of falling | 3061 (6.4%) | 12 484 (7.5%) | 1460 (5.9%) | 4068 (6.4%) | 5300 (9.2%) | 1656 (8.1%) |
|  | Frailty | 10 413 (21.7%) | 46 676 (28.1%) | 6340 (25.6%) | 16 593 (26.2%) | 18 474 (32.0%) | 5268 (25.8%) |
|  | Prior thromboembolism | 7418 (15.5%) | 22 536 (13.6%) | 4269 (17.2%) | 6488 (10.3%) | 9881 (17.1%) | 1898 (9.3%) |
|  | Prior MB/CRNMB | 3105 (6.5%) | 7997 (4.8%) | 1091 (4.4%) | 2760 (4.4%) | 3250 (5.6%) | 896 (4.4%) |
| **Medication history** | | | | | | | |
|  | Number of concomitant drugs | 7.1 ± 4.4 | 6.5 ± 3.9 | 6.2 ± 3.7 | 6.5 ± 3.9 | 6.8 ± 4.1 | 6.3 ± 3.9 |
|  | Polypharmacy (5-9) | 21 715 (45.3%) | 78 942 (47.5%) | 12 018 (48.5%) | 30 382 (48.1%) | 27 154 (47.0%) | 9388 (46.0%) |
|  | Hyperpolypharmacy (≥10) | 11 761 (24.5%) | 31 605 (19.0%) | 3879 (15.6%) | 11 836 (18.7%) | 12 375 (21.4%) | 3515 (17.2%) |
|  | Rate control therapy | 28 222 (58.9%) | 111 046 (66.8%) | 16 256 (65.6%) | 41 575 (65.8%) | 39 498 (68.3%) | 13 717 (67.2%) |
|  | Beta blockers | 26 848 (56.0%) | 105 195 (63.3%) | 15 407 (62.1%) | 39 170 (62.0%) | 37 520 (64.9%) | 13 098 (64.2%) |
|  | Verapamil, diltiazem | 1747 (3.6%) | 6580 (4.0%) | 982 (4.0%) | 2706 (4.3%) | 2232 (3.9%) | 660 (3.2%) |
|  | Digoxin | 2817 (5.9%) | 15 359 (9.2%) | 2134 (8.6%) | 5695 (9.0%) | 5673 (9.8%) | 1857 (9.1%) |
|  | Rhythm control therapy | 11 299 (23.6%) | 56 280 (33.9%) | 8462 (34.1%) | 22 751 (36.0%) | 18 785 (32.5%) | 6282 (30.8%) |
|  | Class I AAD | 2596 (5.4%) | 17 307 (10.4%) | 2754 (11.1%) | 7082 (11.2%) | 5318 (9.2%) | 2153 (10.6%) |
|  | Class III AAD | 9420 (19.7%) | 43 194 (26.0%) | 6385 (25.8%) | 17 495 (27.7%) | 14 759 (25.5%) | 4555 (22.3%) |
|  | Antiplatelet | 19 523 (40.7%) | 73 942 (44.5%) | 10 995 (44.3%) | 27 719 (43.8%) | 26 274 (45.5%) | 8954 (43.9%) |
|  | Acetylsalicylic acid | 18 204 (38.0%) | 69 048 (41.5%) | 10 311 (41.6%) | 25 998 (41.1%) | 24 513 (42.4%) | 8226 (40.3%) |
|  | P2Y12 inhibitor | 2882 (6.0%) | 9852 (5.9%) | 1337 (5.4%) | 3410 (5.4%) | 3614 (6.3%) | 1491 (7.3%) |
|  | ACE inhibitor/ARB | 25 367 (52.9%) | 88 089 (53.0%) | 12 995 (52.4%) | 33 627 (53.2%) | 30 950 (53.5%) | 10 517 (51.6%) |
|  | DHP calcium channel blocker | 15 739 (32.8%) | 52 144 (31.4%) | 7560 (30.5%) | 19 160 (30.3%) | 19 048 (33.0%) | 6376 (31.3%) |
|  | Loop diuretic | 15 754 (32.9%) | 44 401 (26.7%) | 5819 (23.5%) | 17 072 (27.0%) | 16 461 (28.5%) | 5049 (24.7%) |
|  | Non-loop diuretic | 17 589 (36.7%) | 62 836 (37.8%) | 9083 (36.6%) | 23 939 (37.9%) | 22 188 (38.4%) | 7626 (37.4%) |
|  | Proton pump inhibitor | 20 808 (43.4%) | 64 636 (38.9%) | 9061 (36.5%) | 24 221 (38.3%) | 23 462 (40.6%) | 7892 (38.7%) |
|  | NSAID | 12 914 (27.0%) | 39 982 (24.1%) | 5998 (24.2%) | 15 600 (24.7%) | 13 596 (23.5%) | 4788 (23.5%) |
|  | Oral corticosteroids | 10 860 (22.7%) | 30 914 (18.6%) | 4247 (17.1%) | 12 027 (19.0%) | 10 939 (18.9%) | 3701 (18.1%) |
|  | SSRI/SNRI | 6247 (13.0%) | 19 056 (11.5%) | 2759 (11.1%) | 7513 (11.9%) | 6850 (11.9%) | 1934 (9.5%) |
| **Clinical risk score** | | | | | | | |
|  | CHA_2_DS_2_-VASc score | 3.5 ± 1.7 | 3.6 ± 1.6 | 3.6 ± 1.6 | 3.5 ± 1.6 | 3.8 ± 1.7 | 3.5 ± 1.6 |
|  | HAS-BLED score | 2.5 ± 1.3 | 2.6 ± 1.1 | 2.5 ± 1.1 | 2.5 ± 1.1 | 2.7 ± 1.2 | 2.5 ± 1.1 |
|  | Charlson Comorbidity Index | 4.2 ± 2.2 | 4.3 ± 2.0 | 4.3 ± 1.9 | 4.2 ± 1.9 | 4.5 ± 2.0 | 4.2 ± 1.9 |
| **Prescriber** | | | | | | | |
|  | Primary care physician | 28 064 (58.6%) | 60 098 (36.2%) | 9310 (37.5%) | 25 868 (40.9%) | 19 171 (33.2%) | 5748 (28.2%) |
|  | Cardiologist | 9247 (19.3%) | 70 206 (42.2%) | 10 599 (42.7%) | 25 026 (39.6%) | 24 113 (41.7%) | 10 469 (51.3%) |
|  | Other physician | 10 603 (22.1%) | 35 923 (21.6%) | 4886 (19.7%) | 12 333 (19.5%) | 14 519 (25.1%) | 4184 (20.5%) |

**eTable 4:** Baseline characteristics of OAC-naïve non-valvular AF subjects with a CHA_2_DS_2_-VASc score of ≥1 in men or ≥2 in women and a follow-up of ≥1 year.

Data shown as mean ± standard deviation, or counts and percentages. Incident VKA users included 22 813 acenocoumarol, 13 148 warfarin and 11 953 phenprocoumon users. Follow-up ended in case of death, emigration or end of the study period (December 31^st^, 2019). AAD: antiarrhythmic drug; ACE: angiotensin-converting enzyme; ARB: angiotensin II receptor blocker; CRNMB: clinically relevant non-major bleeding; DHP: dihydropyridine; GI: gastrointestinal; MB: major bleeding; NA: not applicable; NOAC: non-vitamin K antagonist oral anticoagulant; NSAID: non-steroidal anti-inflammatory drug; OAC: oral anticoagulant; SNRI: serotonin and norepinephrine reuptake inhibitor; SSRI: selective serotonin reuptake inhibitor; VKA: vitamin K antagonist.

# Supplemental figures

## eFigure 1: Overview of study design

**
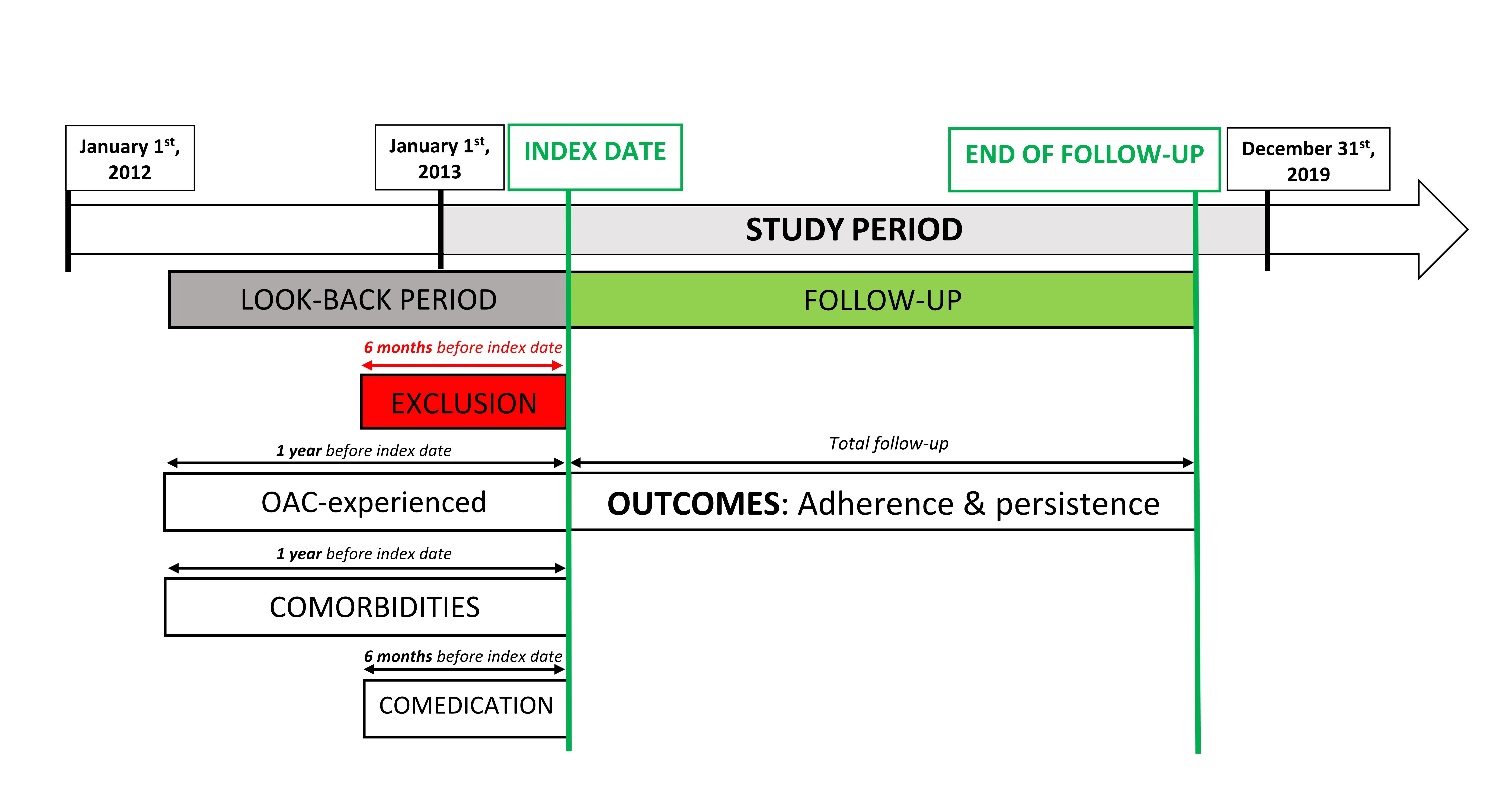
**

**eFigure 1:** Overview of study design.

**Study period:** January 1^st^, 2013 – December 31^st^, 2019. **Look-back period:** Up to 1 year before index date (maximum up until January 1^st^, 2012). **Index date:** First dispensing of oral anticoagulant during study period to subjects ≥45 years. **End of follow-up:** Follow-up ended in case of discontinuation, switching, death, emigration or end of the study period, whichever occurred first. **Exclusion:** Total hip or knee replacement surgery, or diagnosis of deep vein thrombosis or pulmonary embolism ≤6 months before the index date. Moreover, subjects with valvular AF (mechanical prosthetic heart valve or moderate/severe mitral stenosis), end-stage renal disease (chronic kidney disease stage V and/or dialysis), subjects with ≥2 prescription claims of different oral anticoagulant types or dosages on the index date, or subjects treated with NOAC dosages not approved for stroke prevention in AF (e.g. rivaroxaban 10 mg) were excluded. **OAC-experienced:** Subjects were considered as OAC-experienced if an OAC prescription was filled ≤1 year before the index date. Only OAC-naïve subjects were included. **Comorbidities:** Comorbidities were identified using specific ICD-coded diagnoses (e.g. cancer) from the MHD, medical procedure codes (e.g. cancer-related surgery) from the IMA database and/or ATC-coded prescription claims (e.g. antineoplastic drugs) from the IMA database ≤1 year before the index date. **Previous comedication use:** Comedication dispensed up to 6 months before the index date. **Outcome:** Persistence and adherence to oral anticoagulants.

AF: Atrial fibrillation; ATC: Anatomical Therapeutic Chemical Classification; IMA: InterMutualistic Agency; MHD: Minimal Hospital Dataset; NOAC: non-vitamin K antagonist oral anticoagulant; OAC: oral anticoagulant; vitamin K antagonist.

## eFigure 2: Switching between NOAC doses


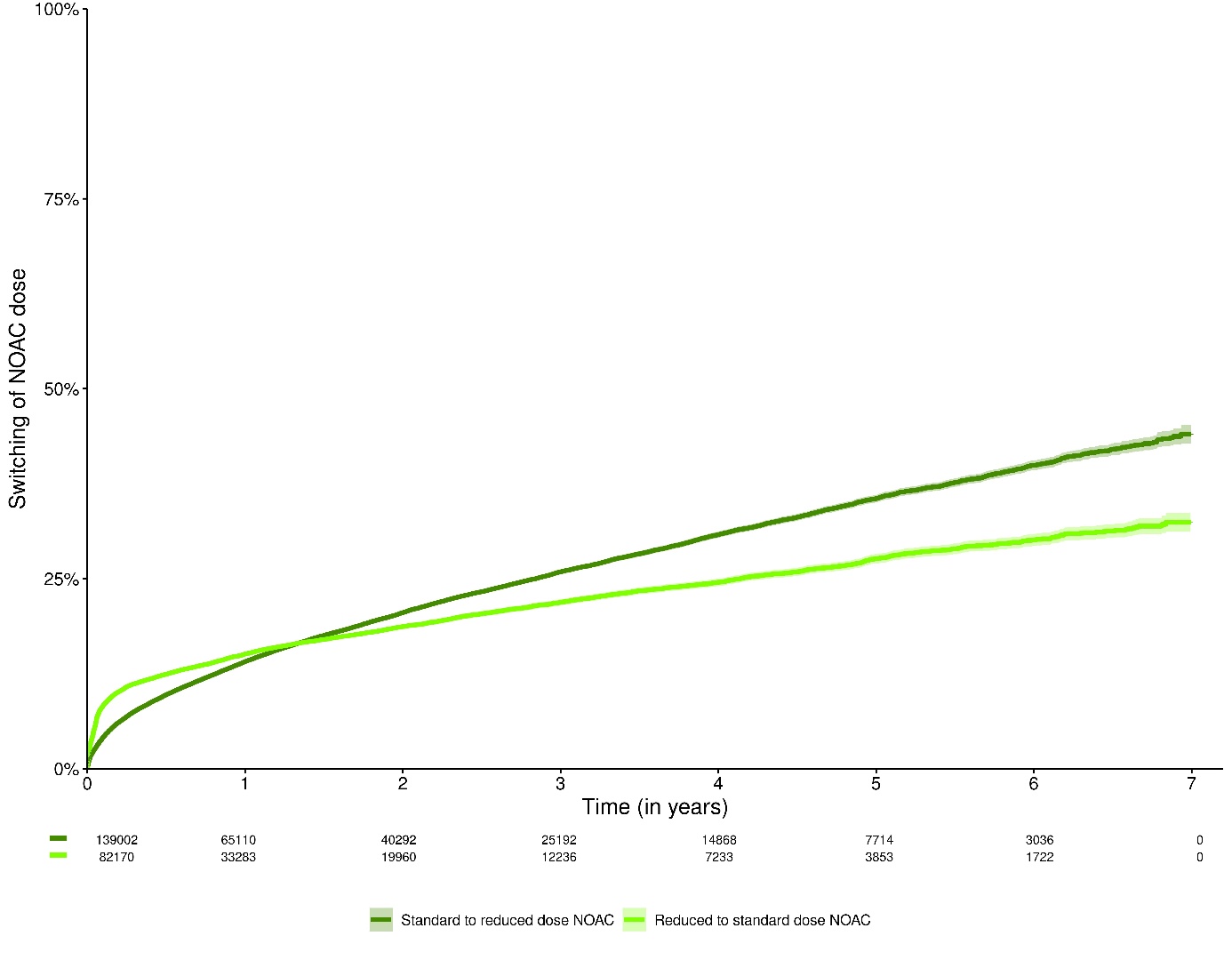


**eFigure 2:** Cumulative incidence curve of switching standard to reduced NOAC dose or vice versa among NOAC users.

Data shown as cumulative incidence with 95% confidence interval and risk table (number of patients at risk). Subjects were censored in case of NOAC discontinuation, switching to VKAs, death, emigration or end of the study period. NOAC: non-vitamin K antagonist oral anticoagulant; VKA: vitamin K antagonist.

## eFigure 3: Persistence (30-day gap) (sensitivity analysis)

**A)**

**
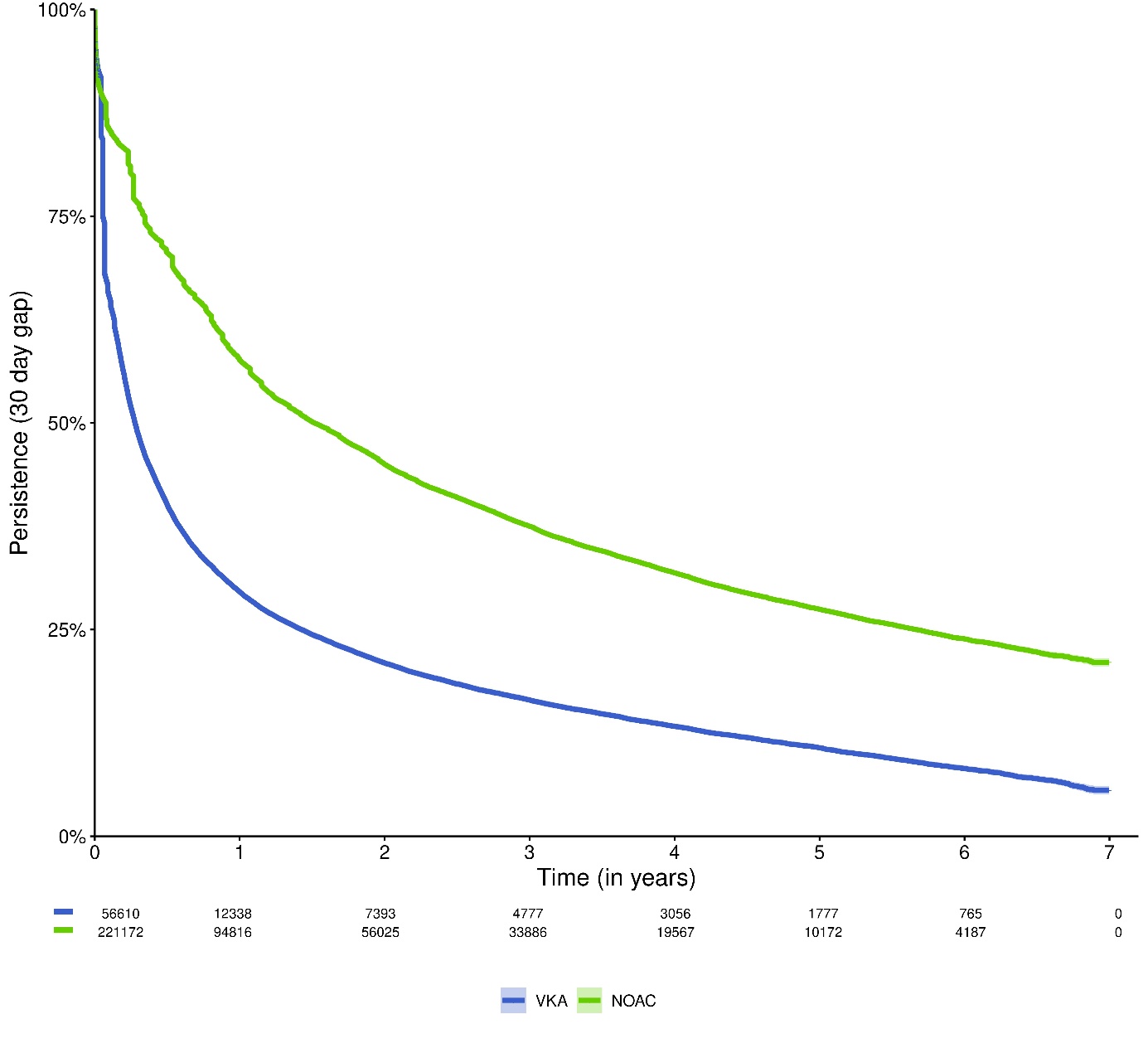
**

**B)**


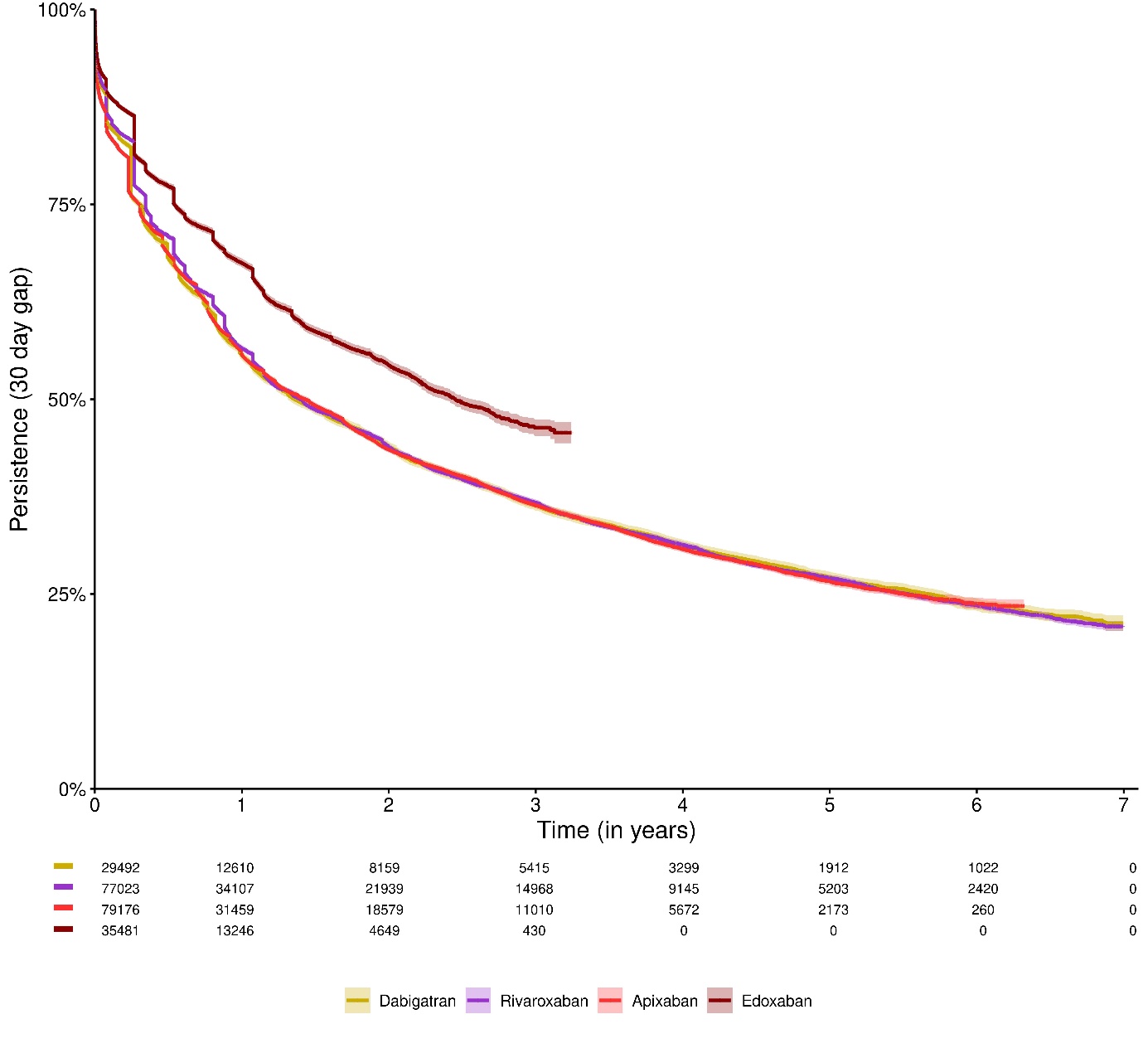


**eFigure 3:** Kaplan-Meier analysis of persistence to **A)** NOACs versus VKAs; and **B)** NOAC types (dabigatran, rivaroxaban, apixaban and edoxaban), defining discontinuation using an arbitrary supply gap of >30 days after the calculated last day of supply, with the possibility to extent this gap for VKAs in case of intervening INR testing at least every 42 days.

Data shown as Kaplan-Meier estimates with 95% confidence interval and risk table (number of patients at risk). In analysis A, subjects were censored in case of switching from NOACs to VKAs or vice versa (allowing switching between NOAC or VKA types), death, emigration or end of the study period; in analysis B, subjects were censored in case of switching to any other OAC type, death, emigration or end of the study period. Due to their respective approval in September 2013 and October 2016, and the study period ending on December 31^st^, 2019, the maximum follow-up duration of apixaban and edoxaban users was limited to 6.3 years and 3.25 years, respectively. INR: International Normalized Ratio; NOAC: non-vitamin K antagonist oral anticoagulant; OAC: oral anticoagulant; VKA: vitamin K antagonist.

## eFigure 4: Persistence (90-day gap) (sensitivity analysis)

**A)**

**
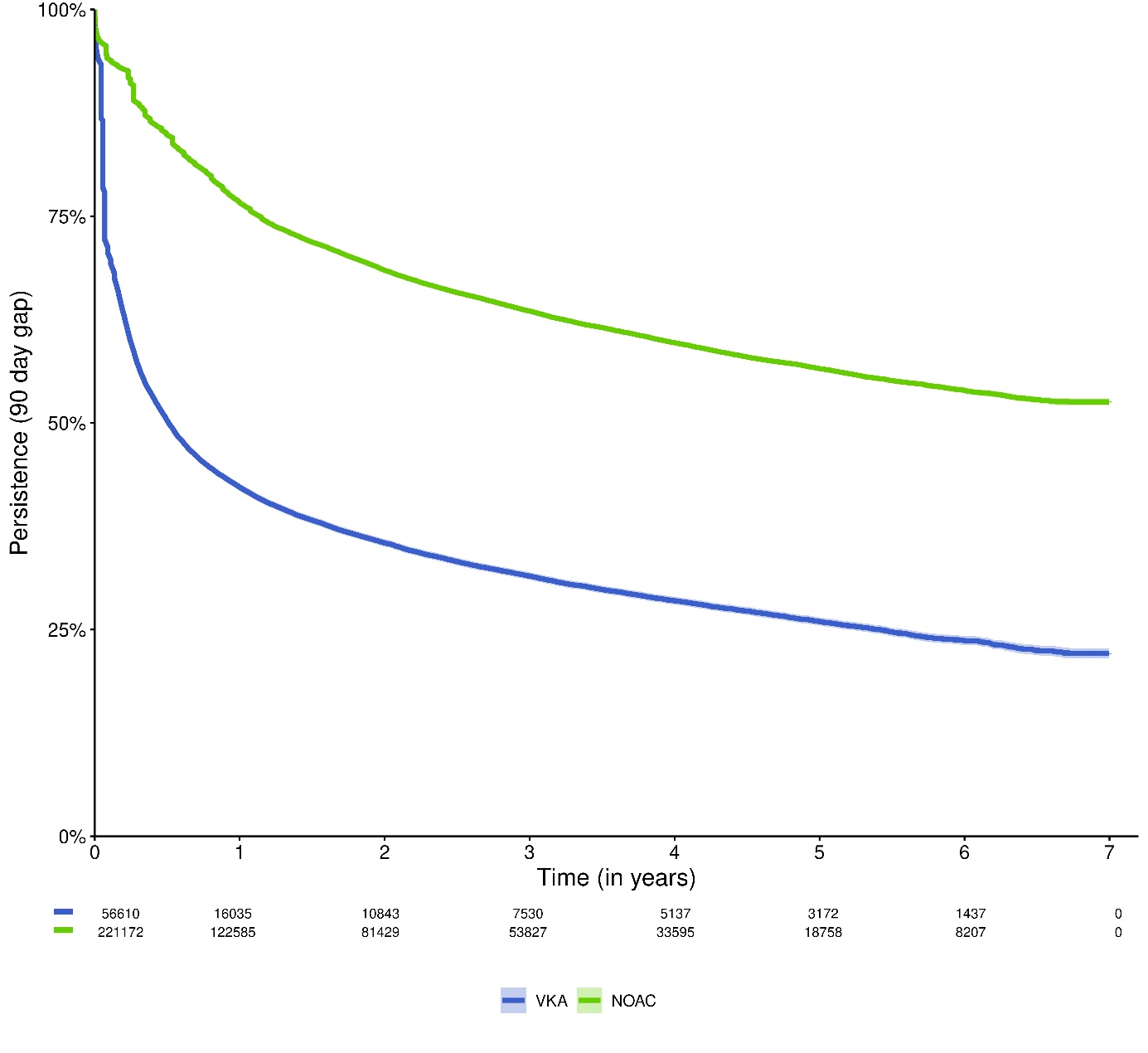
**

**B)**


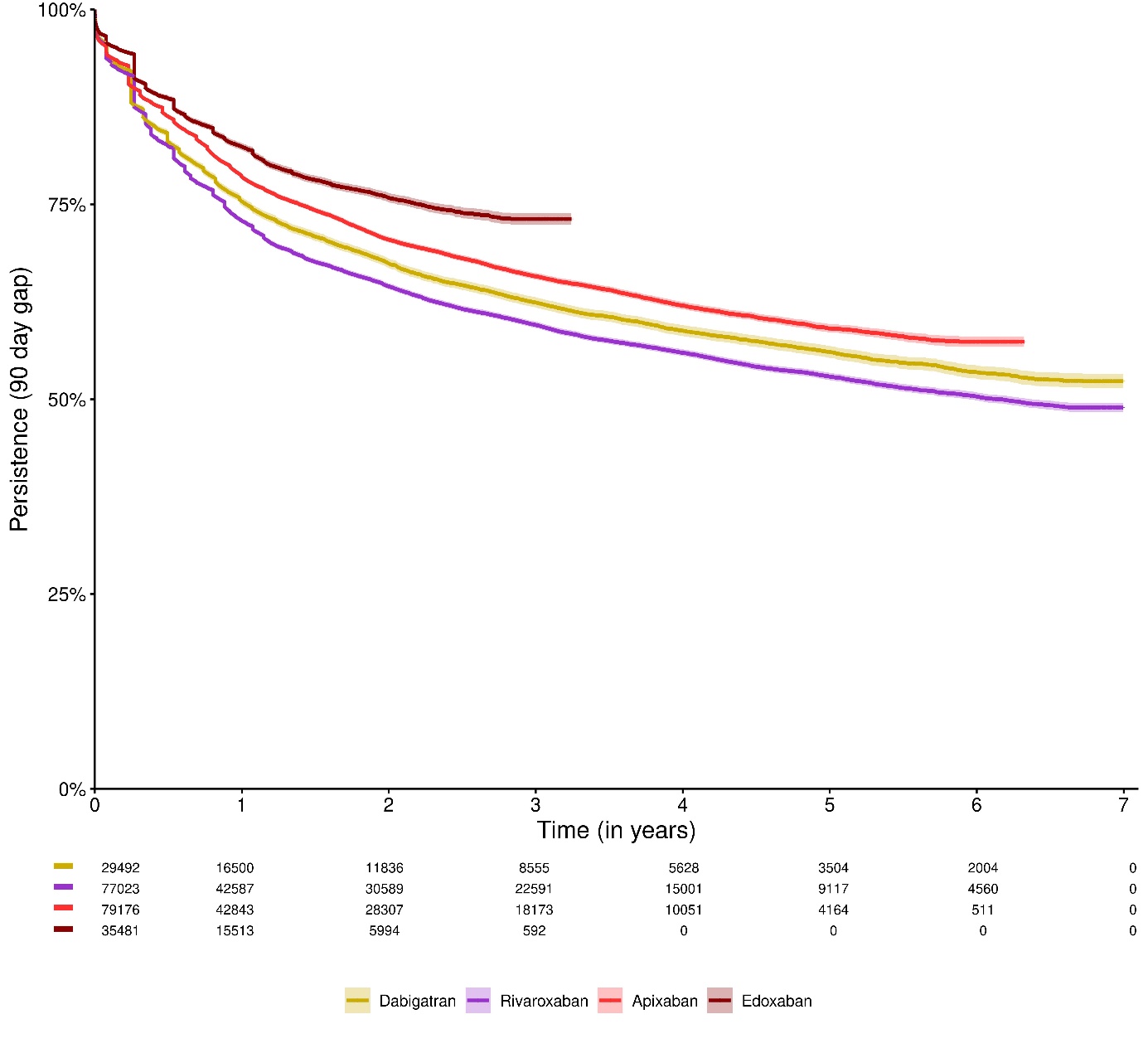


**eFigure 4:** Kaplan-Meier analysis of persistence to **A)** NOACs versus VKAs; and **B)** NOAC types (dabigatran, rivaroxaban, apixaban and edoxaban), defining discontinuation using an arbitrary supply gap of >90 days after the calculated last day of supply, with the possibility to extent this gap for VKAs in case of intervening INR testing at least every 42 days.

Data shown as Kaplan-Meier estimates with 95% confidence interval and risk table (number of patients at risk). In analysis A, subjects were censored in case of switching from NOACs to VKAs or vice versa (allowing switching between NOAC or VKA types), death, emigration or end of the study period; in analysis B, subjects were censored in case of switching to any other OAC type, death, emigration or end of the study period. Due to their respective approval in September 2013 and October 2016, and the study period ending on December 31^st^, 2019, the maximum follow-up duration of apixaban and edoxaban users was limited to 6.3 years and 3.25 years, respectively. NOAC: non-vitamin K antagonist oral anticoagulant; OAC: oral anticoagulant; VKA: vitamin K antagonist.

## eFigure 5: Adherence (PDC) in persistent and non-persistent NOAC users (sensitivity analysis)

**A)**
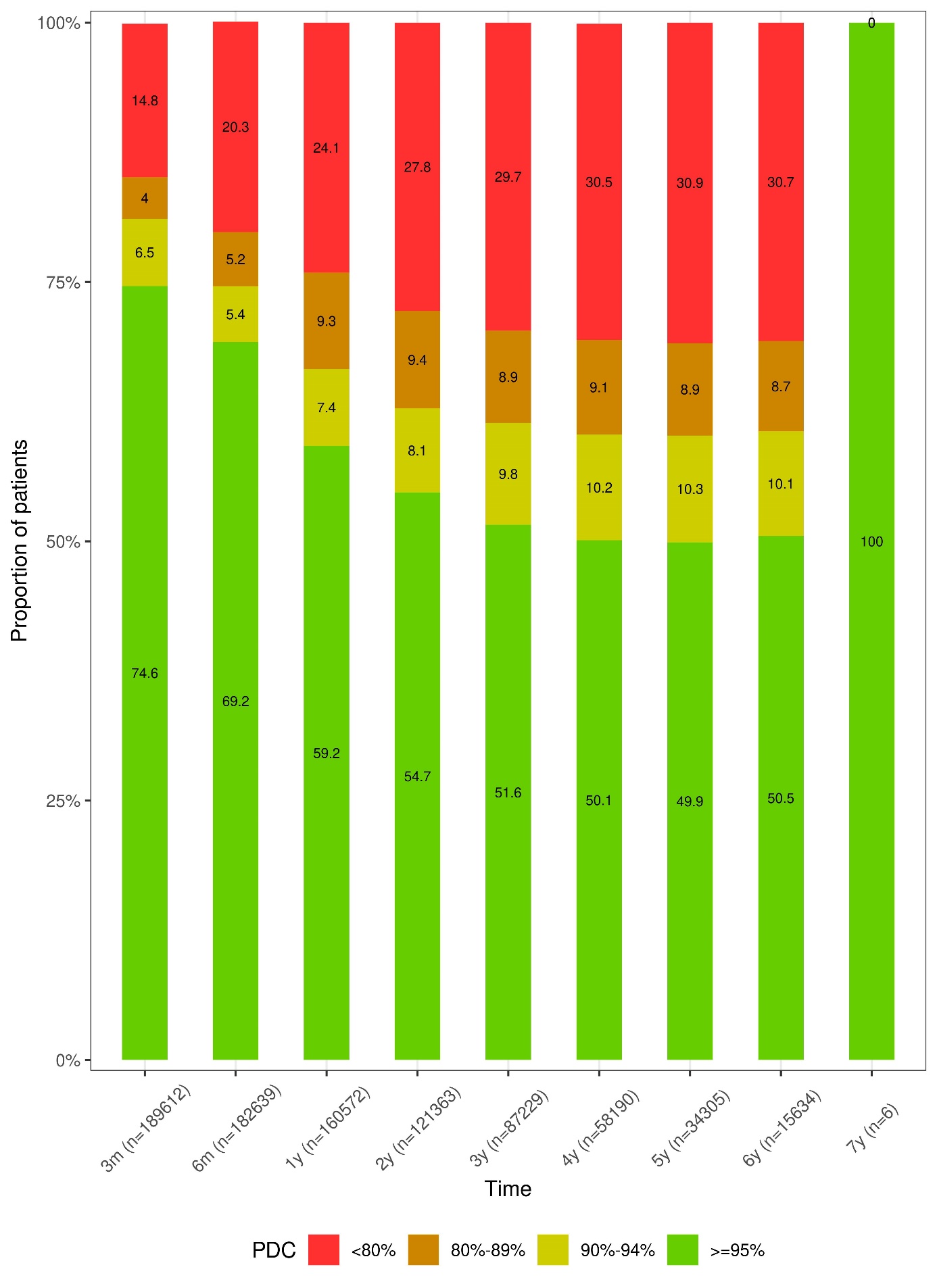


**B)**

**
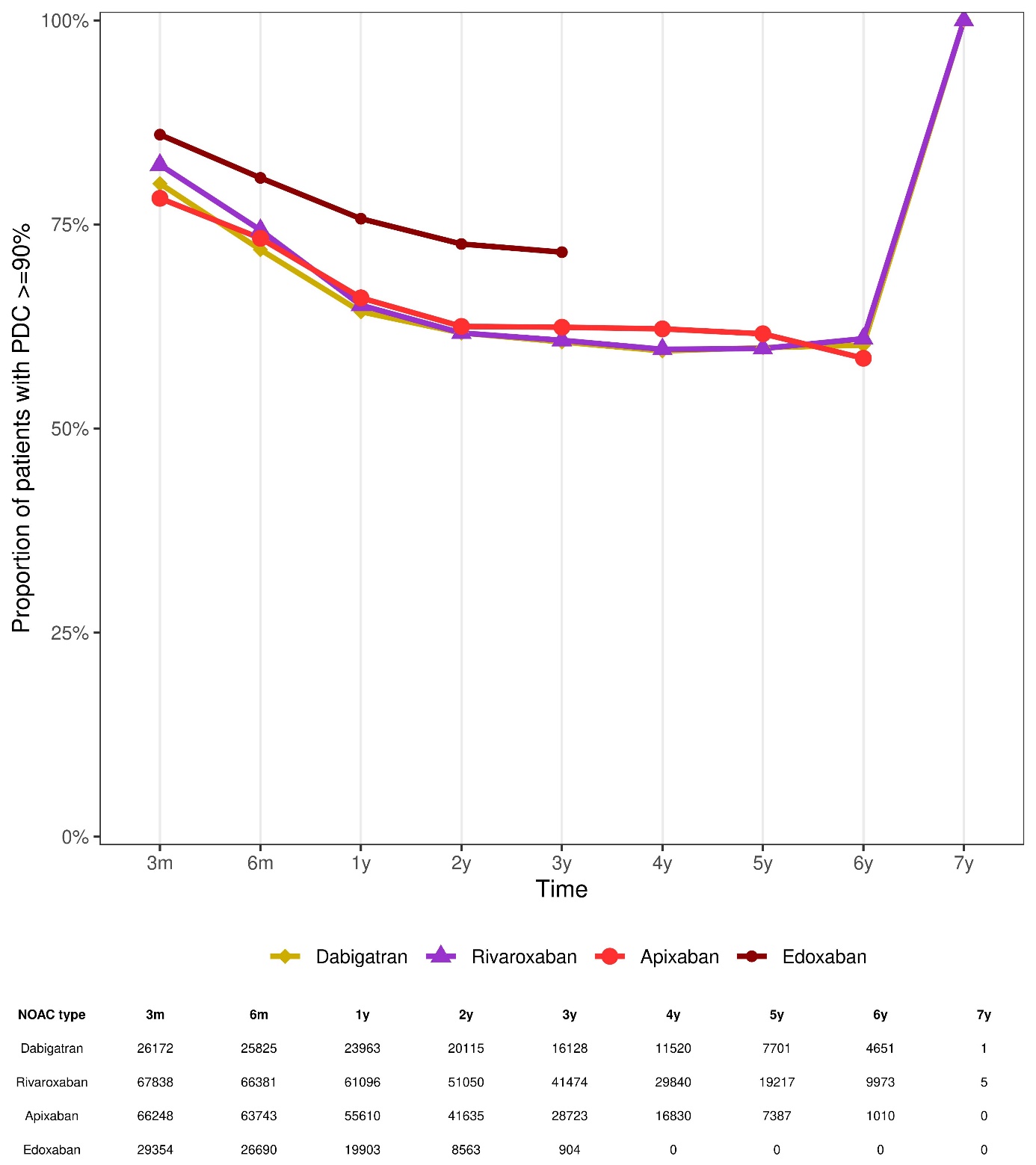
**

**eFigure 5:** Therapy adherence of **A)** persistent and non-persistent NOAC users, categorized according to a PDC of <80%, 80-<90%, 90-<94% and ≥95%, and **B)** the proportion of persistent and non-persistent dabigatran, rivaroxaban, apixaban and edoxaban users with a PDC of ≥90% at specific time intervals (sensitivity analysis).

Subjects were censored in case of death, emigration or end of the study period. Due to their respective approval in September 2013 and October 2016, and the study period ending on December 31^st^, 2019, the maximum follow-up duration of apixaban and edoxaban users was limited to 6.3 years and 3.25 years, respectively. Only persistent and non-persistent NOAC users with a follow-up at least corresponding with the examined time interval were investigated (e.g. PDC after 1 year in the subgroup of subjects with ≥1 year of follow-up), as illustrated by the number of investigated subjects (n) per time point. M: month; NOAC: non-vitamin K antagonist oral anticoagulant; OAC: oral anticoagulant; PDC: proportion of days covered; VKA: vitamin K antagonist; y: year.

## eFigure 6: Adherence (PDC) (ambulatory and hospital dispensings) (sensitivity analysis)

**A)**
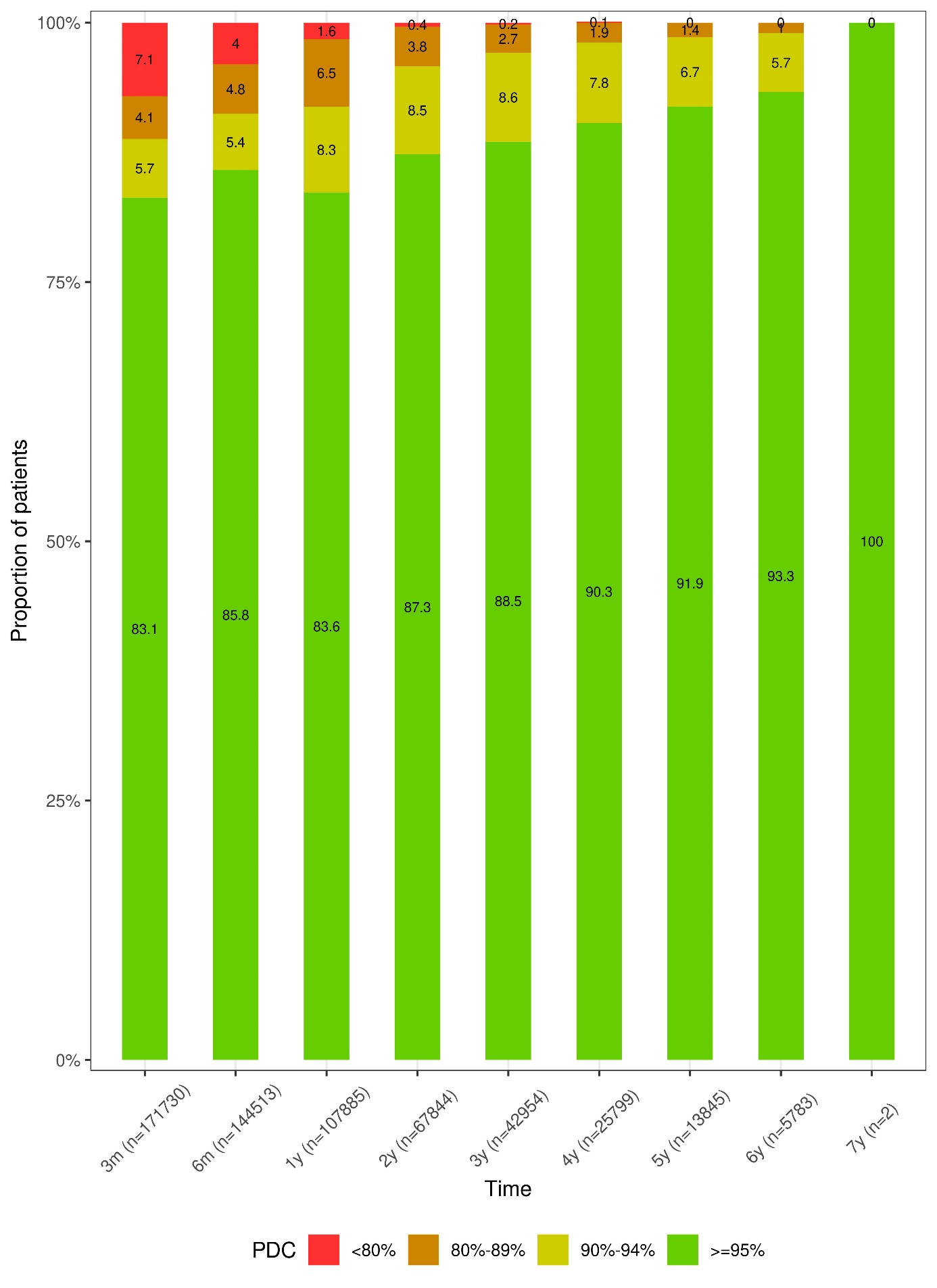


**B)**


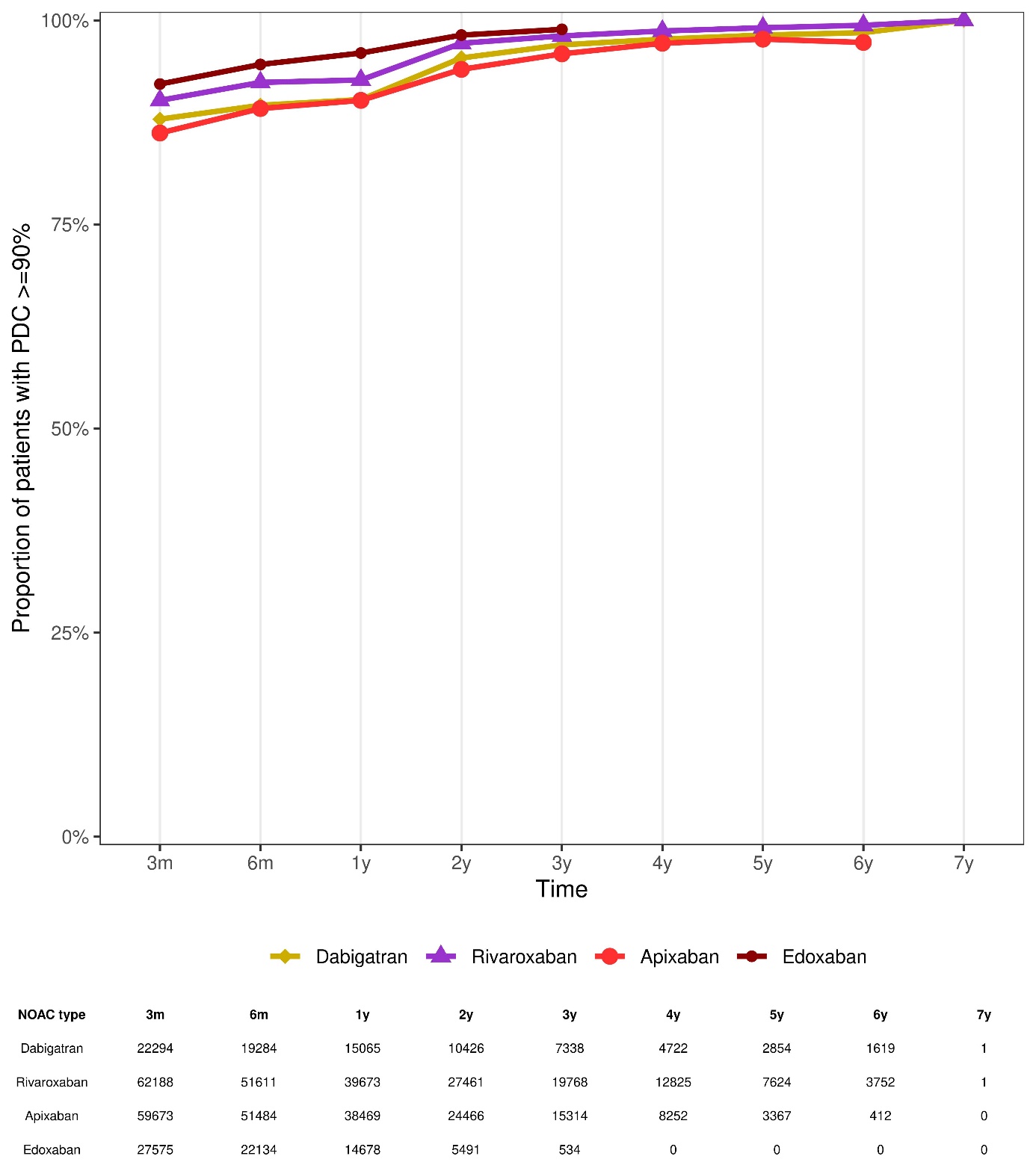


**eFigure 6:** Therapy adherence of **A)** persistent NOAC users, categorized according to a PDC of <80%, 80-<90%, 90-<94% and ≥95%, and **B)** the proportion of persistent dabigatran, rivaroxaban, apixaban and edoxaban users with a PDC of ≥90% at specific time intervals, considering both ambulatory and hospital dispensings of NOACs for the PDC calculation (sensitivity analysis).

Subjects were censored in case of NOAC discontinuation, switching to any other OAC type, death, emigration or end of the study period. Due to their respective approval in September 2013 and October 2016, and the study period ending on December 31^st^, 2019, the maximum follow-up duration of apixaban and edoxaban users was limited to 6.3 years and 3.25 years, respectively. Only persistent NOAC users with a follow-up at least corresponding with the examined time interval were investigated (e.g. PDC after 1 year in the subgroup of subjects with ≥1 year of follow-up), as illustrated by the number of investigated subjects (n) per time point. M: month; NOAC: non-vitamin K antagonist oral anticoagulant; OAC: oral anticoagulant; PDC: proportion of days covered; VKA: vitamin K antagonist; y: year.

## eFigure 7: Persistence in subjects with a non-sex-related CHA_2_DS_2_-VASc score of ≥2 (sensitivity analysis)

**A)**


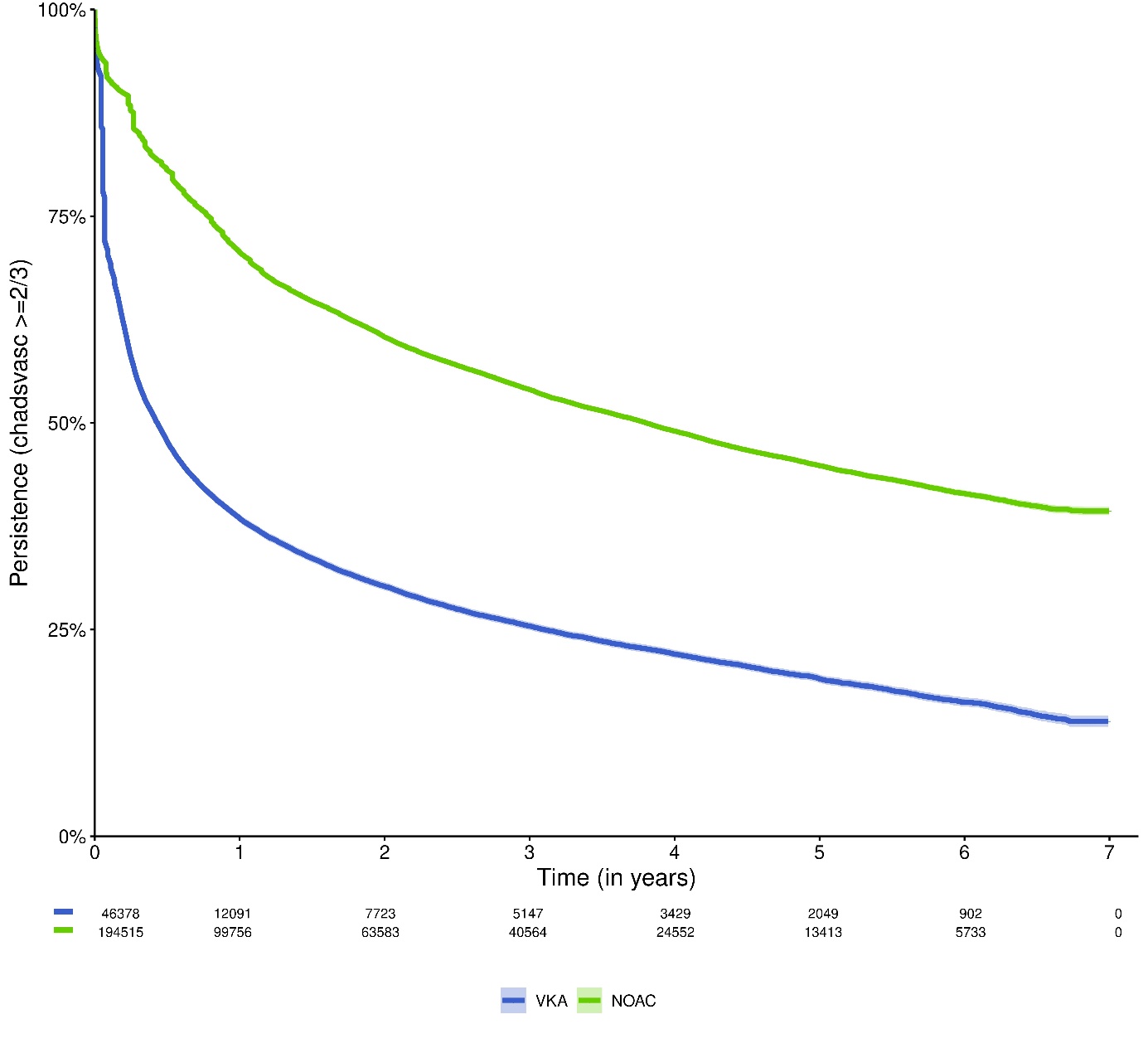


**B)**


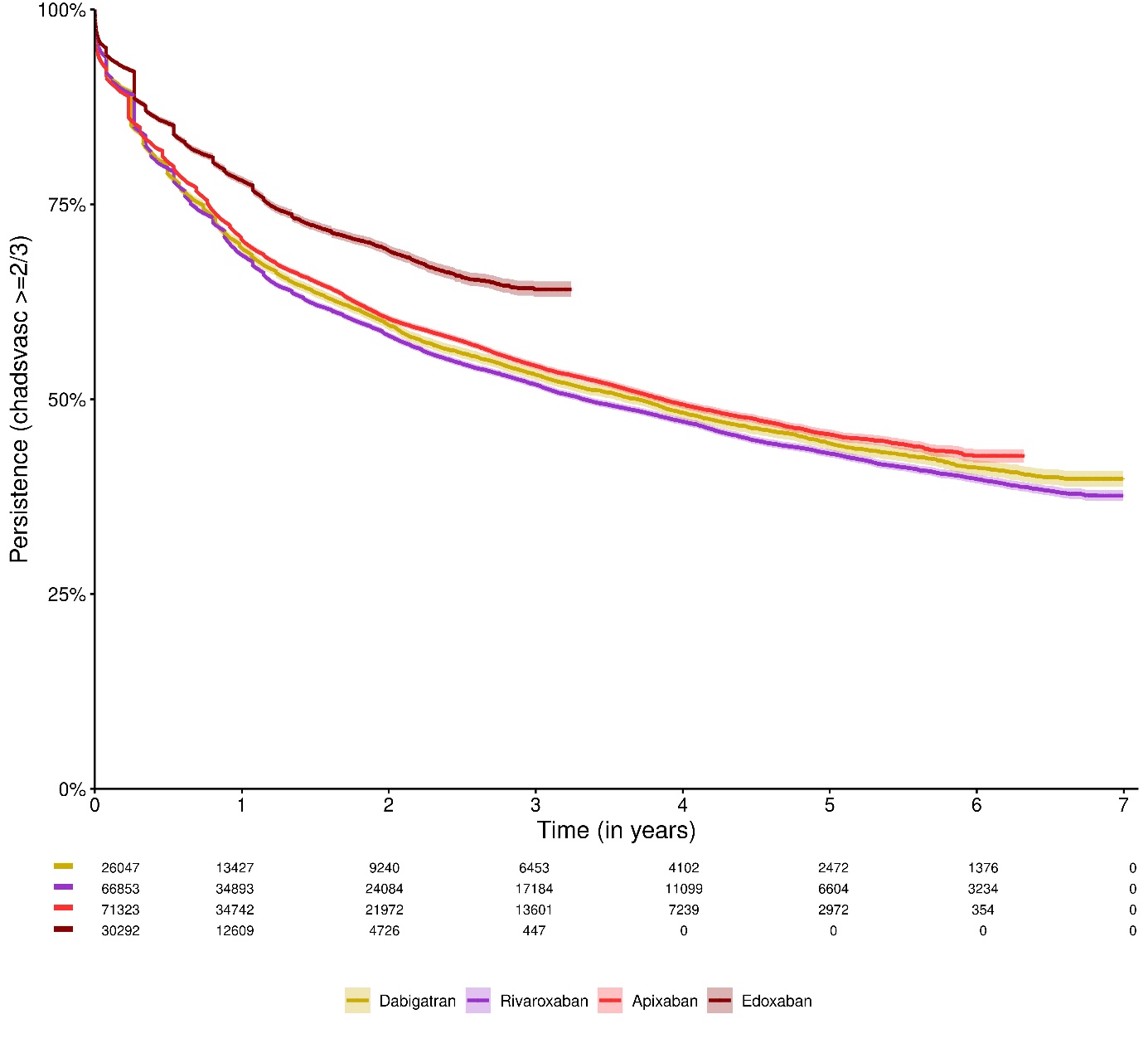


**eFigure 7:** Kaplan-Meier analysis of persistence to **A)** NOACs versus VKAs; and **B)** NOAC types (dabigatran, rivaroxaban, apixaban and edoxaban) in subjects with a CHA_2_DS_2_-VASc score of ≥2 in men and ≥3 in women (sensitivity analysis).

Data shown as Kaplan-Meier estimates with 95% confidence interval and risk table (number of patients at risk). In analysis A, subjects were censored in case of switching from NOACs to VKAs or vice versa (allowing switching between NOAC or VKA types), death, emigration or end of the study period; in analysis B, subjects were censored in case of switching to any other OAC type, death, emigration or end of the study period. Due to their respective approval in September 2013 and October 2016, and the study period ending on December 31^st^, 2019, the maximum follow-up duration of apixaban and edoxaban users was limited to 6.3 years and 3.25 years, respectively. NOAC: non-vitamin K antagonist oral anticoagulant; OAC: oral anticoagulant; VKA: vitamin K antagonist.

eFigure 8: Reinitiation in subjects with a non-sex-related CHA_2_DS_2_-VASc score of ≥2 (sensitivity analysis)

**A)**

**
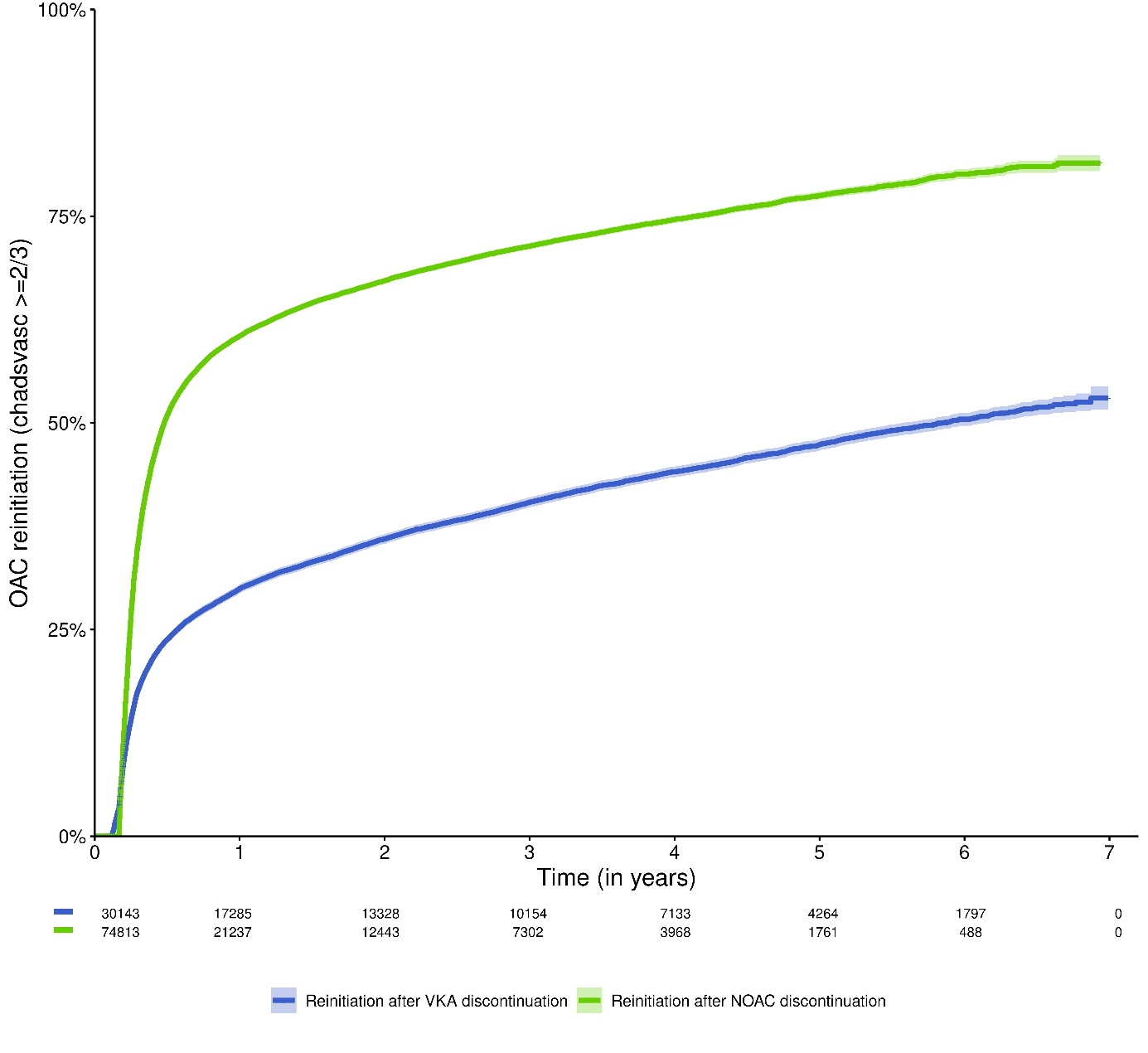
**

**B)**


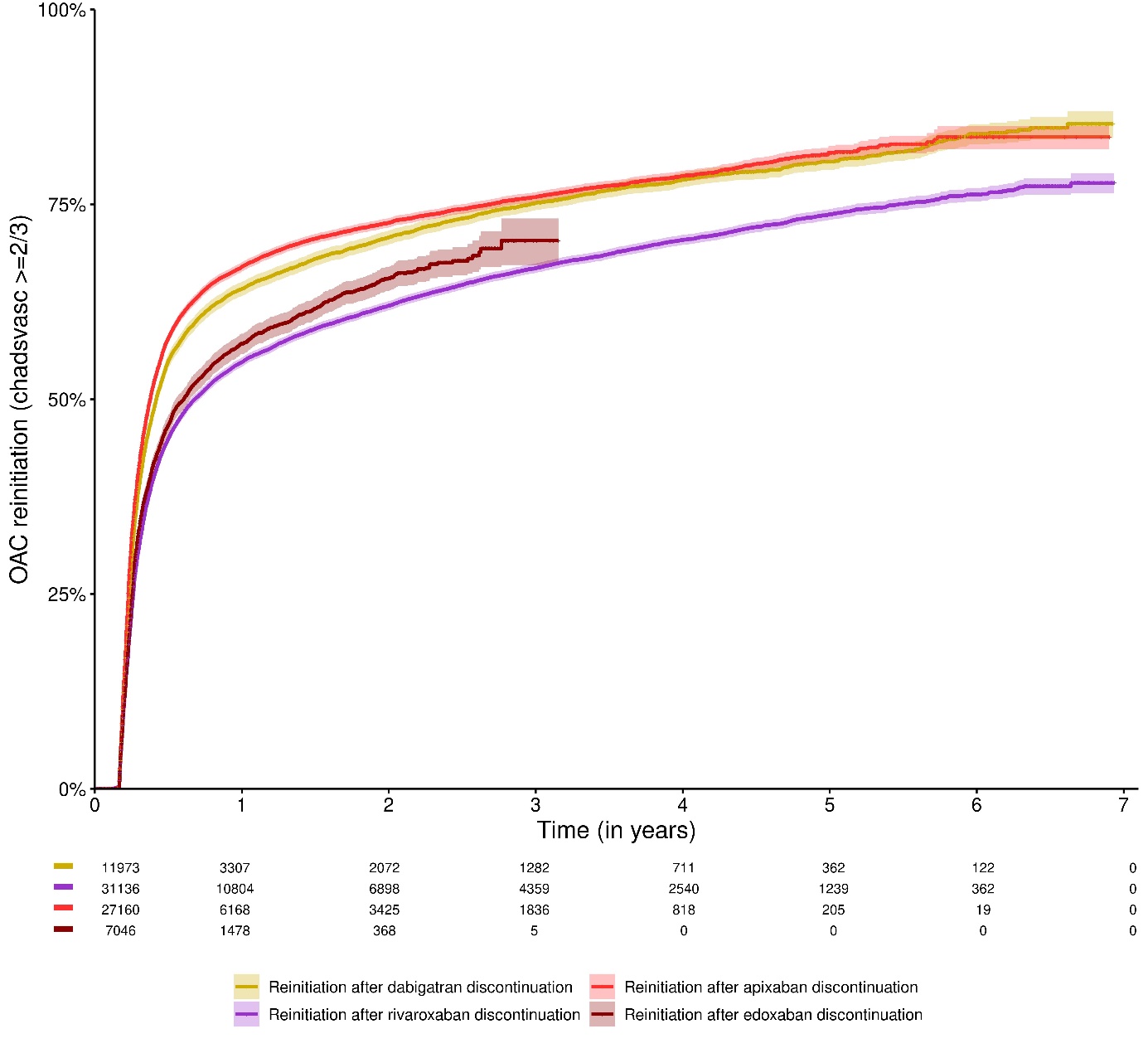


**eFigure 8:** Cumulative incidence curve of reinitiation of an anticoagulant after discontinuation of **A)** NOACs and VKAs; and **B)** NOAC types (dabigatran, rivaroxaban, apixaban and edoxaban) in subjects with a CHA_2_DS_2_-VASc score of ≥2 in men and ≥3 in women (sensitivity analysis).

Data shown as cumulative incidence with 95% confidence interval and risk table (number of patients at risk). Subjects were included at the date of discontinuation. Subjects were censored in case of death, emigration or end of the study period. The initial lag period in the cumulative incidence curves (60 days for NOACs, 42-60 days for VKAs) is due to the definition of discontinuation (arbitrary supply gap of >60 days after the calculated last day of supply, with the possibility to extent this gap for VKAs in case of intervening INR testing at least every 42 days). Due to their respective approval in September 2013 and October 2016, and the study period ending on December 31^st^, 2019, the maximum follow-up duration of apixaban and edoxaban users was limited to 6.3 years and 3.25 years, respectively. NOAC: non-vitamin K antagonist oral anticoagulant; OAC: oral anticoagulant; VKA: vitamin K antagonist.

eFigure 9: Switching in subjects with a non-sex-related CHA_2_DS_2_-VASc score of ≥2 (sensitivity analysis)

**A)**


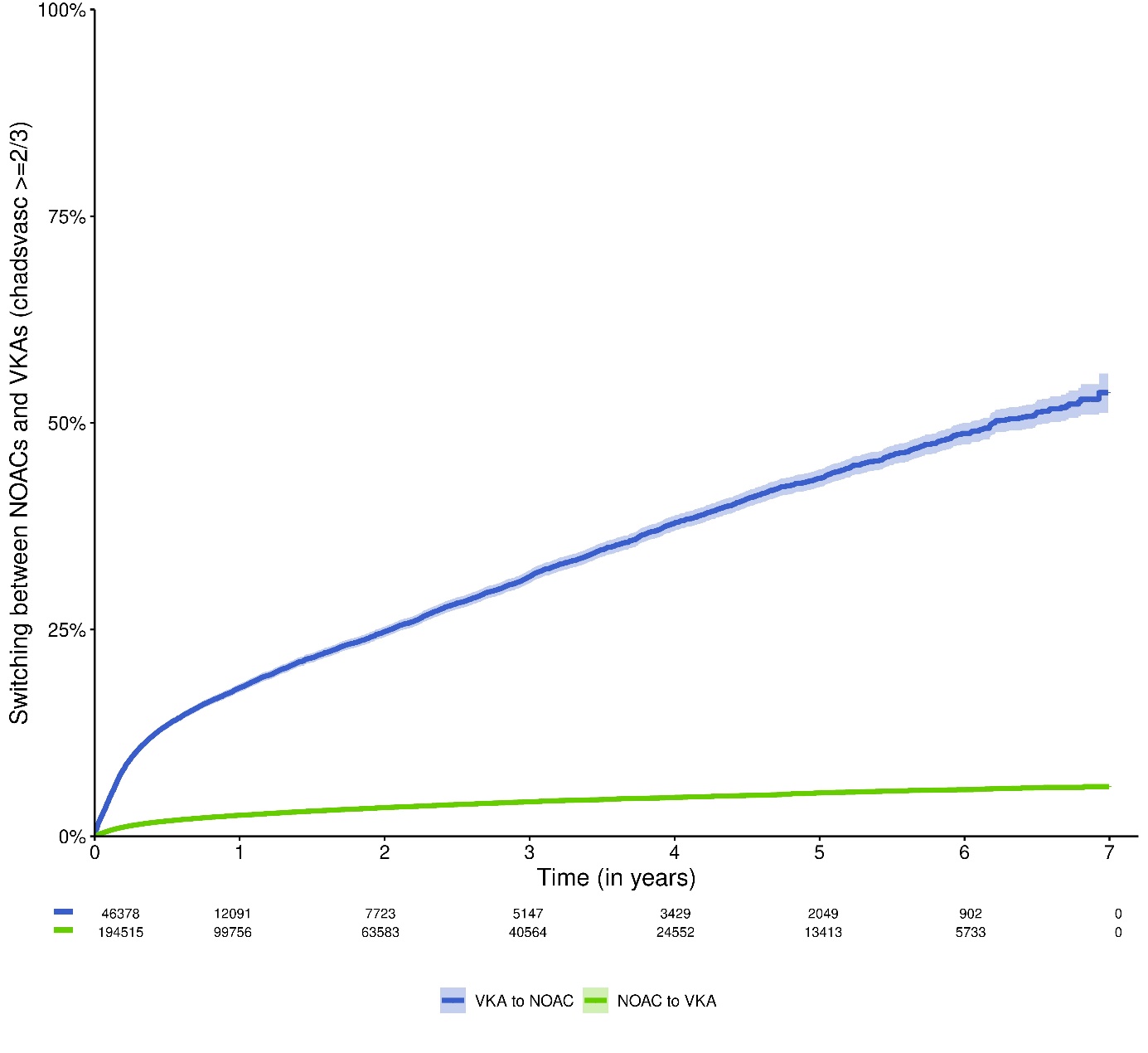


**B)**

**
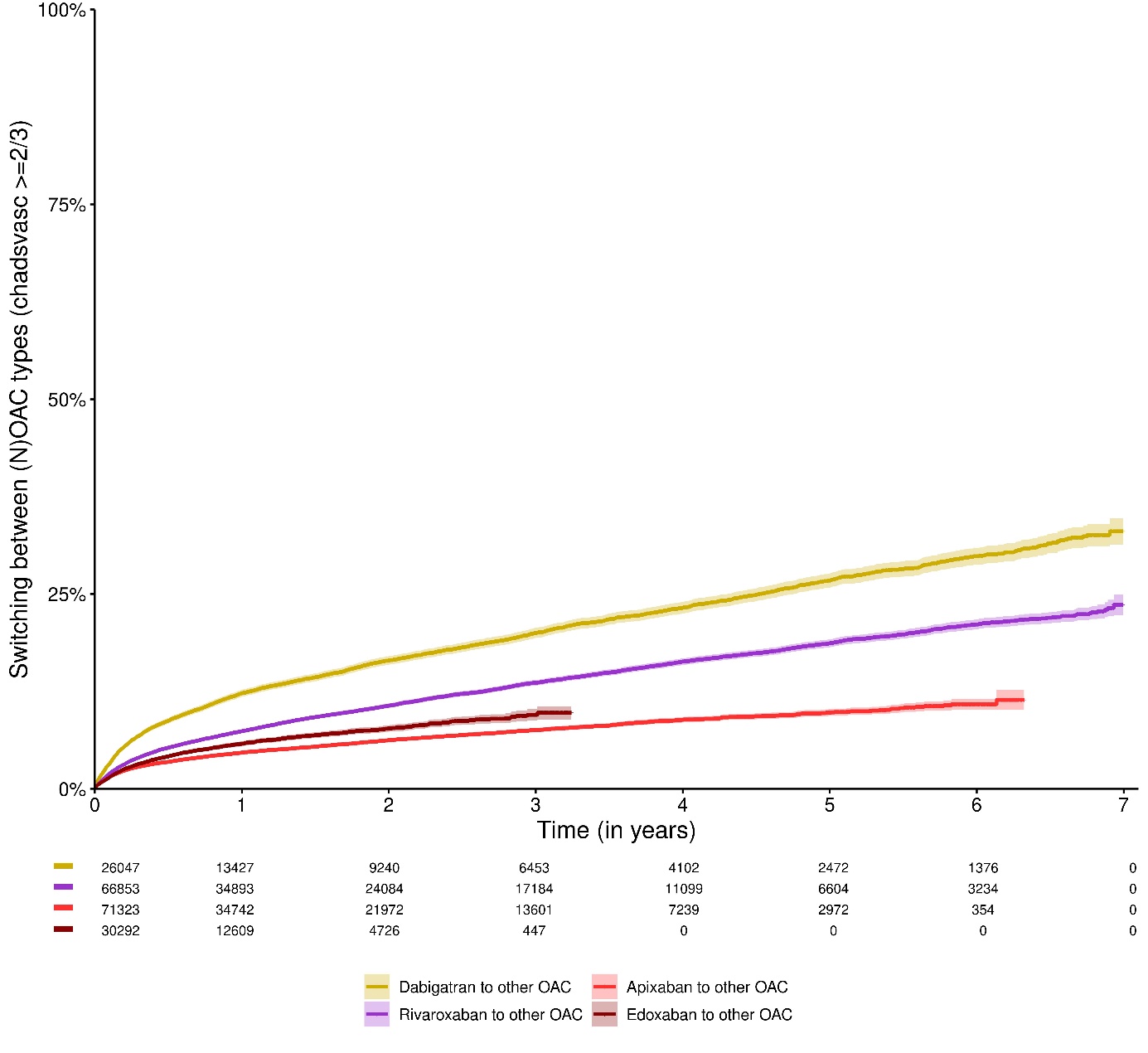
**

**eFigure 9:** Cumulative incidence curve of switching **A)** NOACs to VKAs or vice versa; and **B)** switching between any type of OAC (e.g. from dabigatran to rivaroxaban) in subjects with a CHA_2_DS_2_-VASc score of ≥2 in men and ≥3 in women (sensitivity analysis).

Data shown as cumulative incidence with 95% confidence interval and risk table (number of patients at risk). In analysis A, subjects were censored in case of discontinuation of NOACs or VKAs (allowing switching between NOAC types and VKA types, respectively), death, emigration or end of the study period; in analysis B, subjects were censored in case of discontinuation of the index OAC type, death, emigration or end of the study period. Due to their respective approval in September 2013 and October 2016, and the study period ending on December 31^st^, 2019, the maximum follow-up duration of apixaban and edoxaban users was limited to 6.3 years and 3.25 years, respectively. NOAC: non-vitamin K antagonist oral anticoagulant; OAC: oral anticoagulant; VKA: vitamin K antagonist.

eFigure 10: Adherence (PDC) in subjects with a non-sex-related CHA_2_DS_2_-VASc score of ≥2 (sensitivity analysis)

**A)**

**
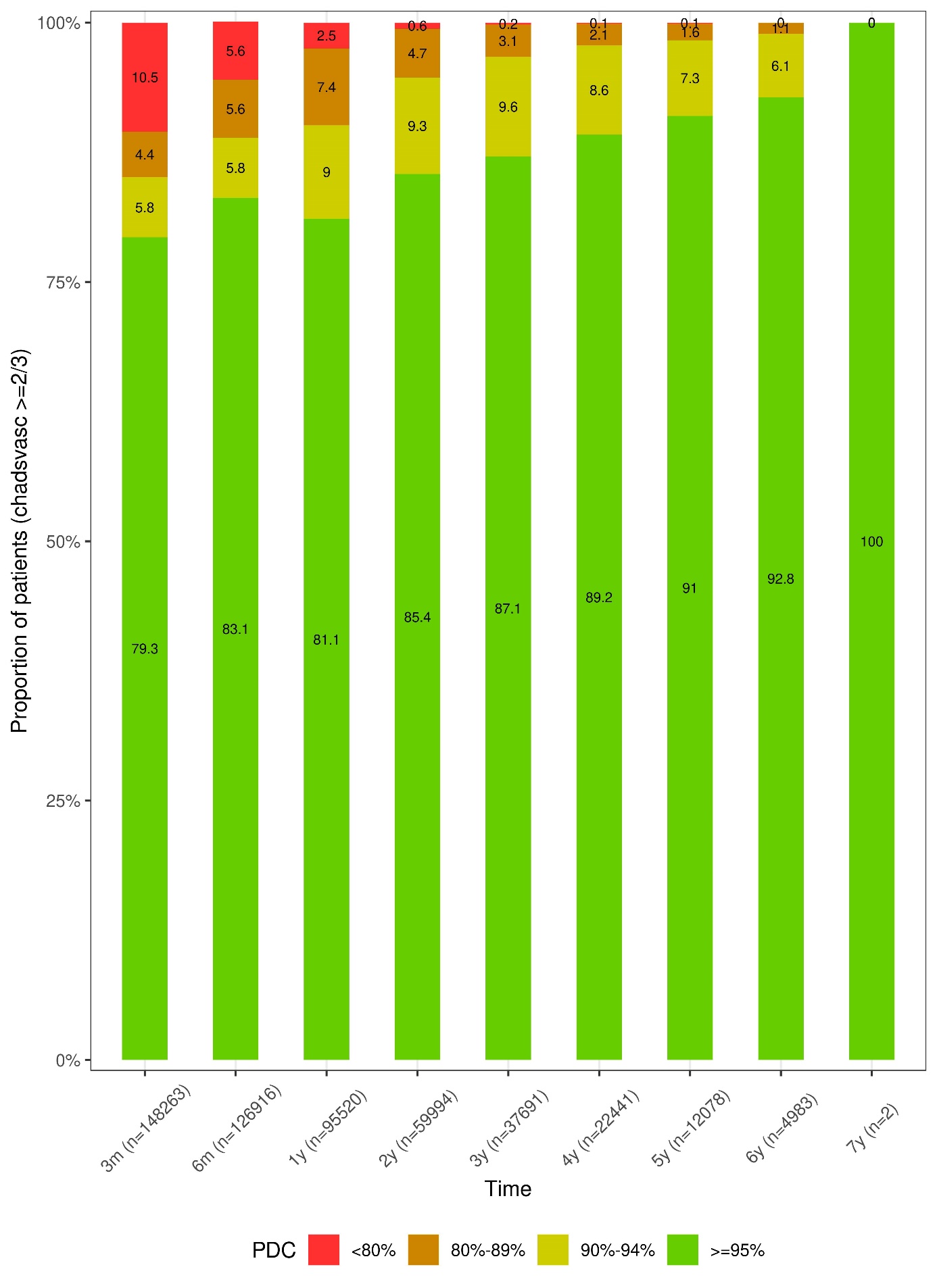
**

**B)**


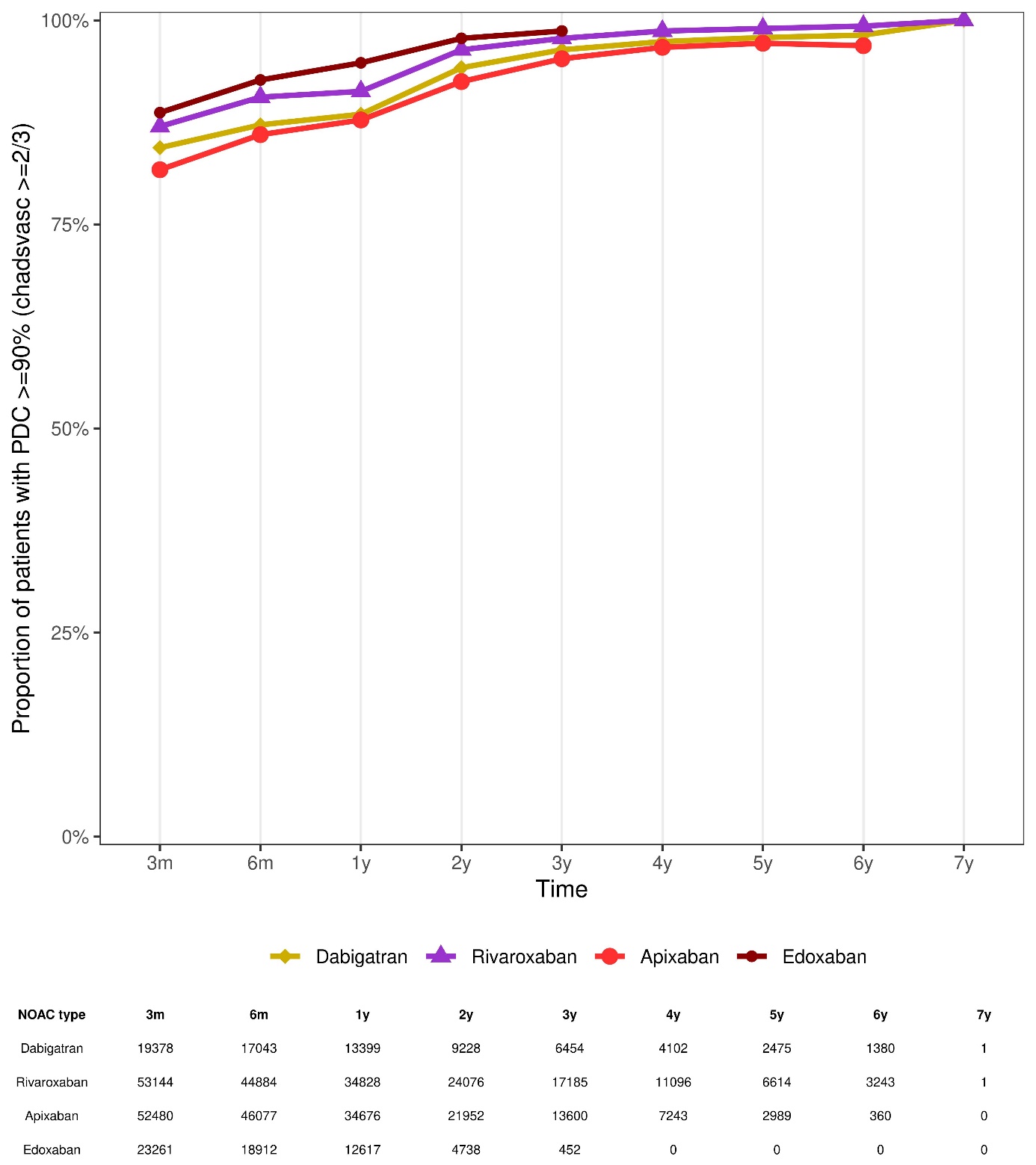


**eFigure 10:** Therapy adherence of **A)** persistent NOAC users, categorized according to a PDC of <80%, 80-<90%, 90-<94% and ≥95%, and **B)** the proportion of persistent dabigatran, rivaroxaban, apixaban and edoxaban users with a PDC of ≥90% at specific time intervals in NOAC-treated subjects with a CHA_2_DS_2_-VASc score of ≥2 in men and ≥3 in women (sensitivity analysis).

Subjects were censored in case of NOAC discontinuation, switching to any other OAC type, death, emigration or end of the study period. Due to their respective approval in September 2013 and October 2016, and the study period ending on December 31^st^, 2019, the maximum follow-up duration of apixaban and edoxaban users was limited to 6.3 years and 3.25 years, respectively. Only persistent NOAC users with a follow-up at least corresponding with the examined time interval were investigated (e.g. PDC after 1 year in the subgroup of subjects with ≥1 year of follow-up), as illustrated by the number of investigated subjects (n) per time point. M: month; NOAC: non-vitamin K antagonist oral anticoagulant; OAC: oral anticoagulant; PDC: proportion of days covered; VKA: vitamin K antagonist; y: year.

## eFigure 11: Persistence in subjects having initiated treatment between October 2016 and December 2019 (sensitivity analysis)

**A)**


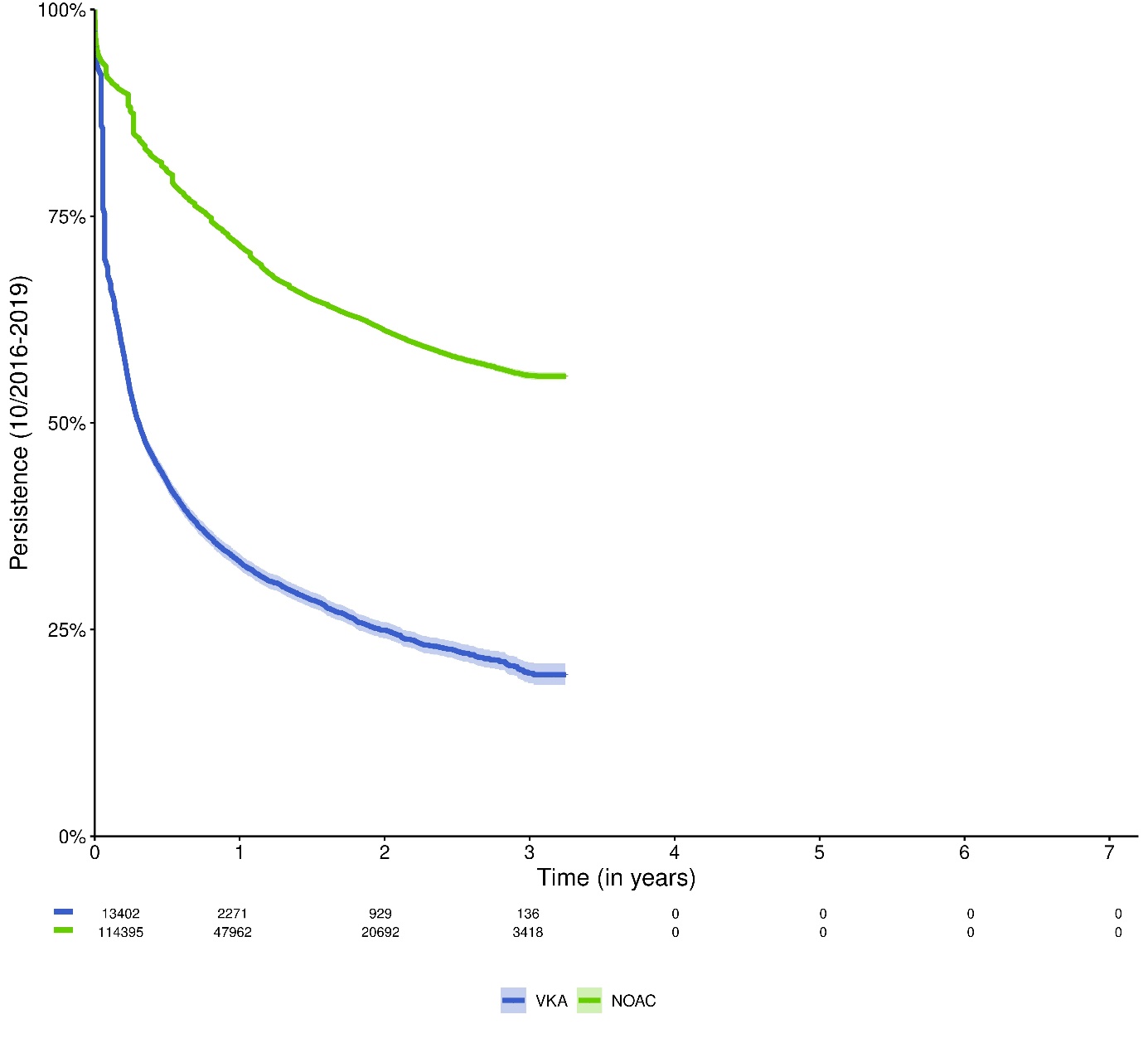


**B)**


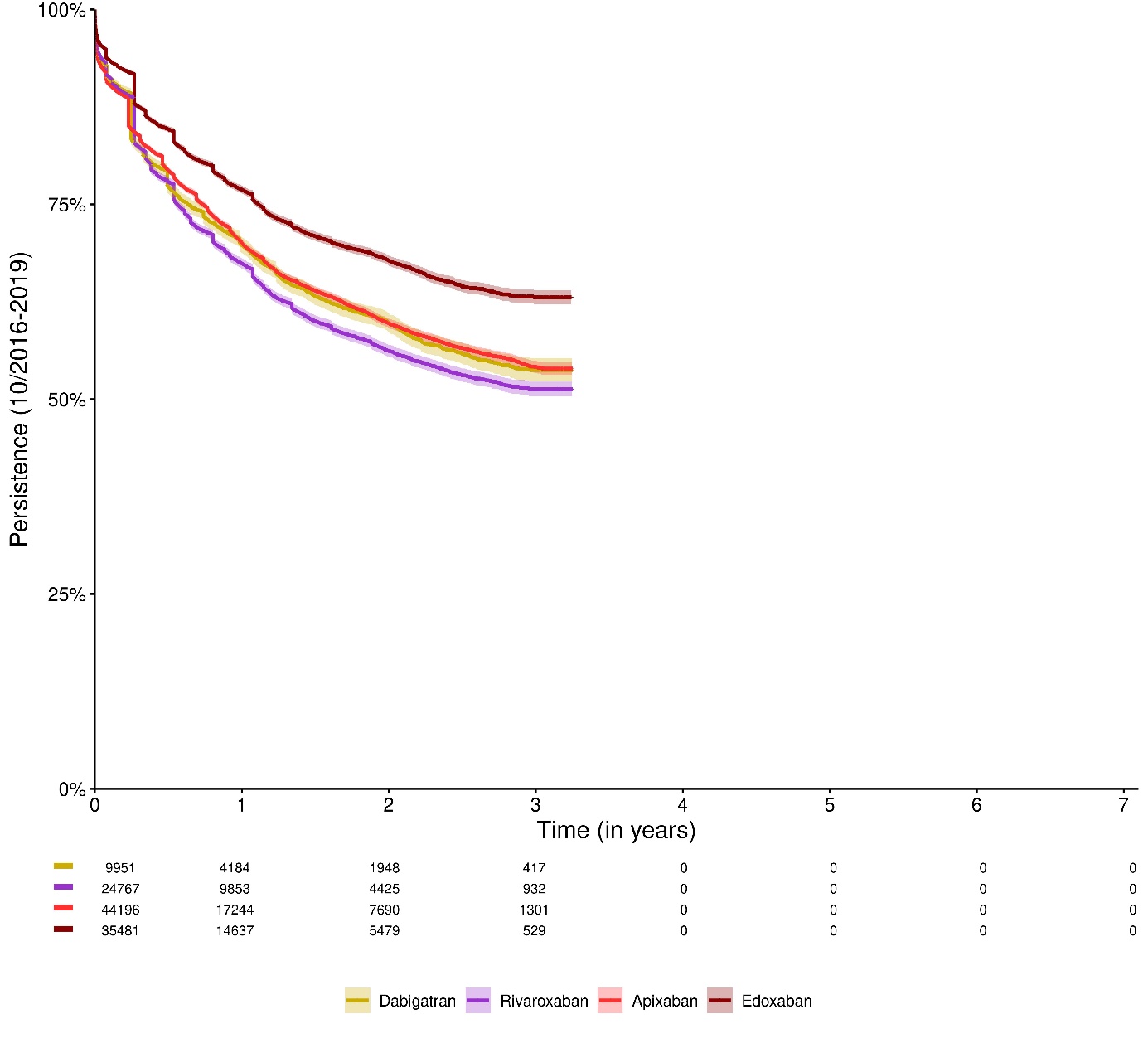


**eFigure 11:** Kaplan-Meier analysis of persistence to **A)** NOACs versus VKAs; and **B)** NOAC types (dabigatran, rivaroxaban, apixaban and edoxaban) in subjects having initiated treatment between October 1^st^, 2016 and December 31^st^, 2019 (sensitivity analysis).

Data shown as Kaplan-Meier estimates with 95% confidence interval and risk table (number of patients at risk). In analysis A, subjects were censored in case of switching from NOACs to VKAs or vice versa (allowing switching between NOAC or VKA types), death, emigration or end of the study period; in analysis B, subjects were censored in case of switching to any other OAC type, death, emigration or end of the study period. NOAC: non-vitamin K antagonist oral anticoagulant; OAC: oral anticoagulant; VKA: vitamin K antagonist.

eFigure 12: Reinitiation in subjects having initiated treatment between October 2016 and December 2019 (sensitivity analysis)

**A)**

**
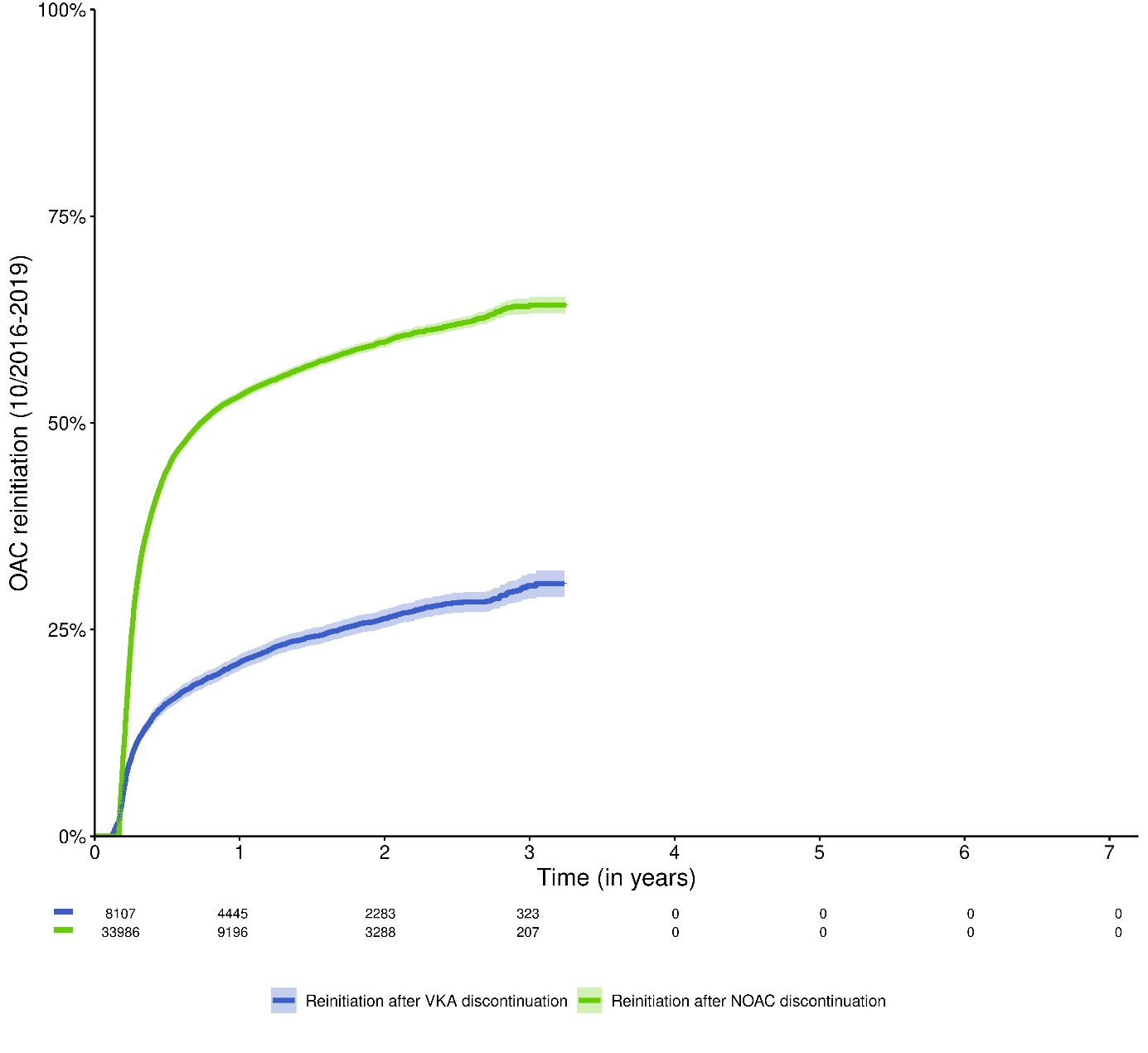
**

**B)**


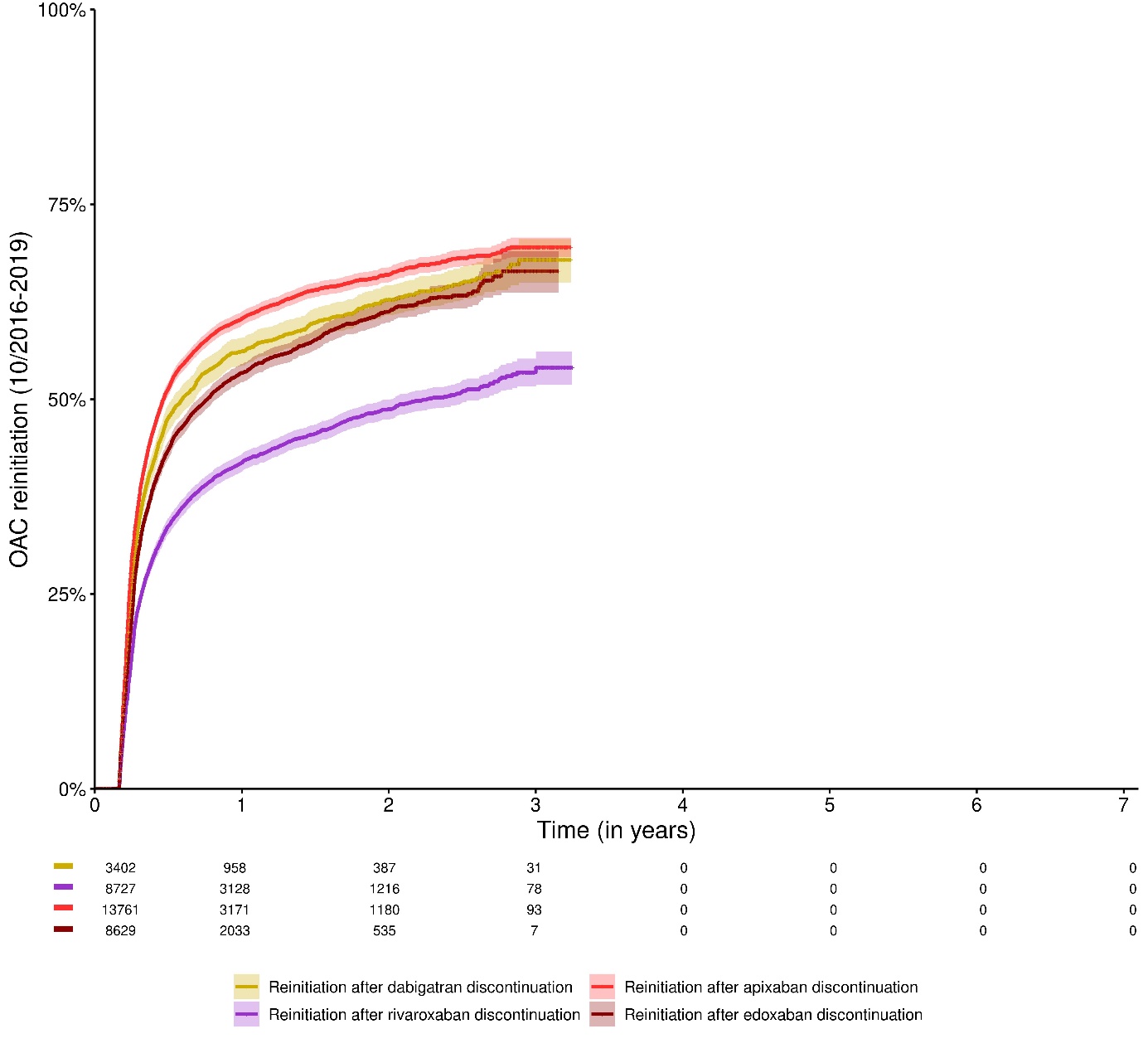


**eFigure 12:** Cumulative incidence curve of reinitiation of an anticoagulant after discontinuation of **A)** NOACs and VKAs; and **B)** NOAC types (dabigatran, rivaroxaban, apixaban and edoxaban) in subjects having initiated treatment between October 1^st^, 2016 and December 31^st^, 2019 (sensitivity analysis).

Data shown as cumulative incidence with 95% confidence interval and risk table (number of patients at risk). Subjects were included at the date of discontinuation. Subjects were censored in case of death, emigration or end of the study period. The initial lag period in the cumulative incidence curves (60 days for NOACs, 42-60 days for VKAs) is due to the definition of discontinuation (arbitrary supply gap of >60 days after the calculated last day of supply, with the possibility to extent this gap for VKAs in case of intervening INR testing at least every 42 days). NOAC: non-vitamin K antagonist oral anticoagulant; OAC: oral anticoagulant; VKA: vitamin K antagonist.

eFigure 13: Switching in subjects having initiated treatment between October 2016 and December 2019 (sensitivity analysis)

**A)**


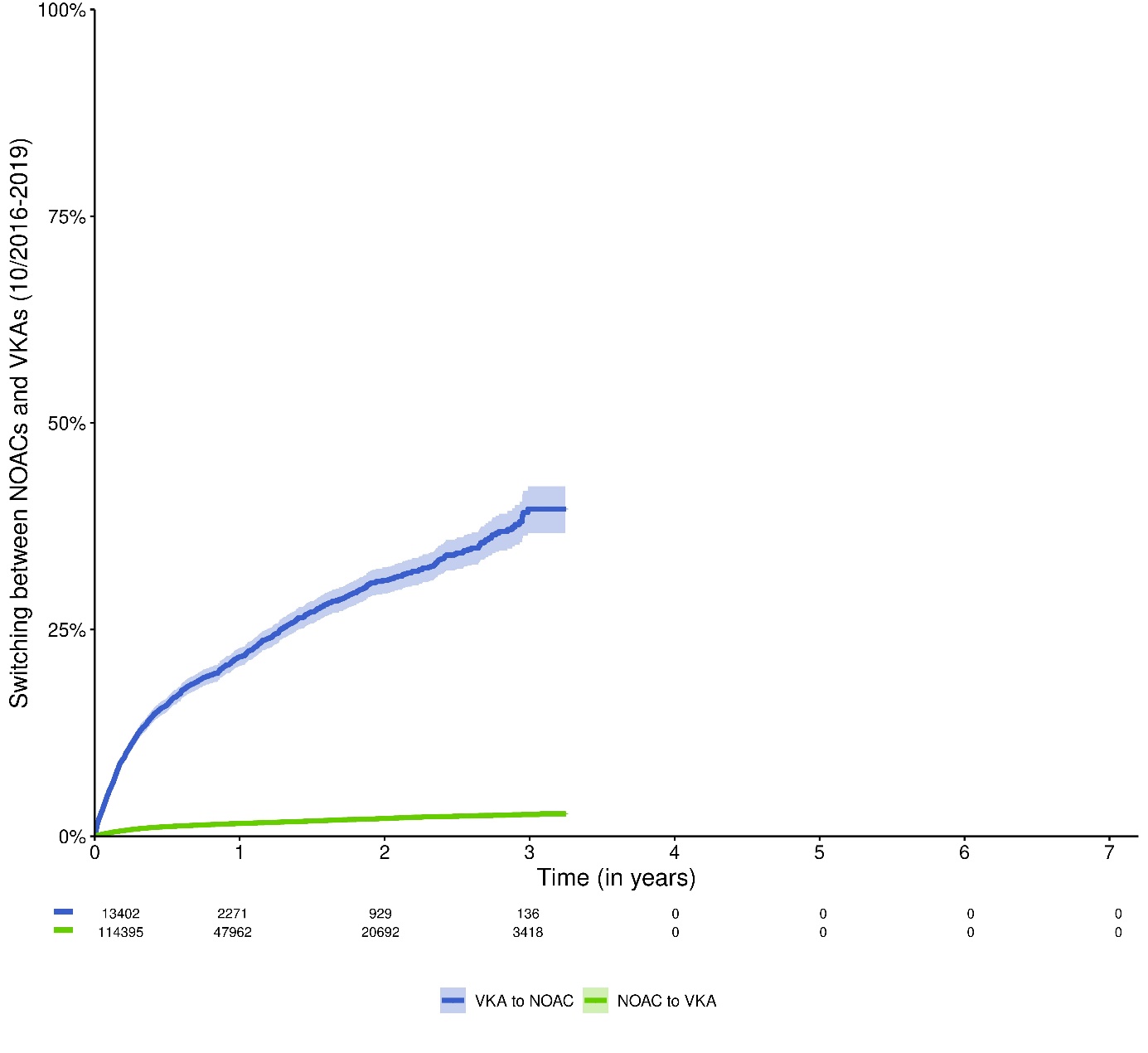


**B)**


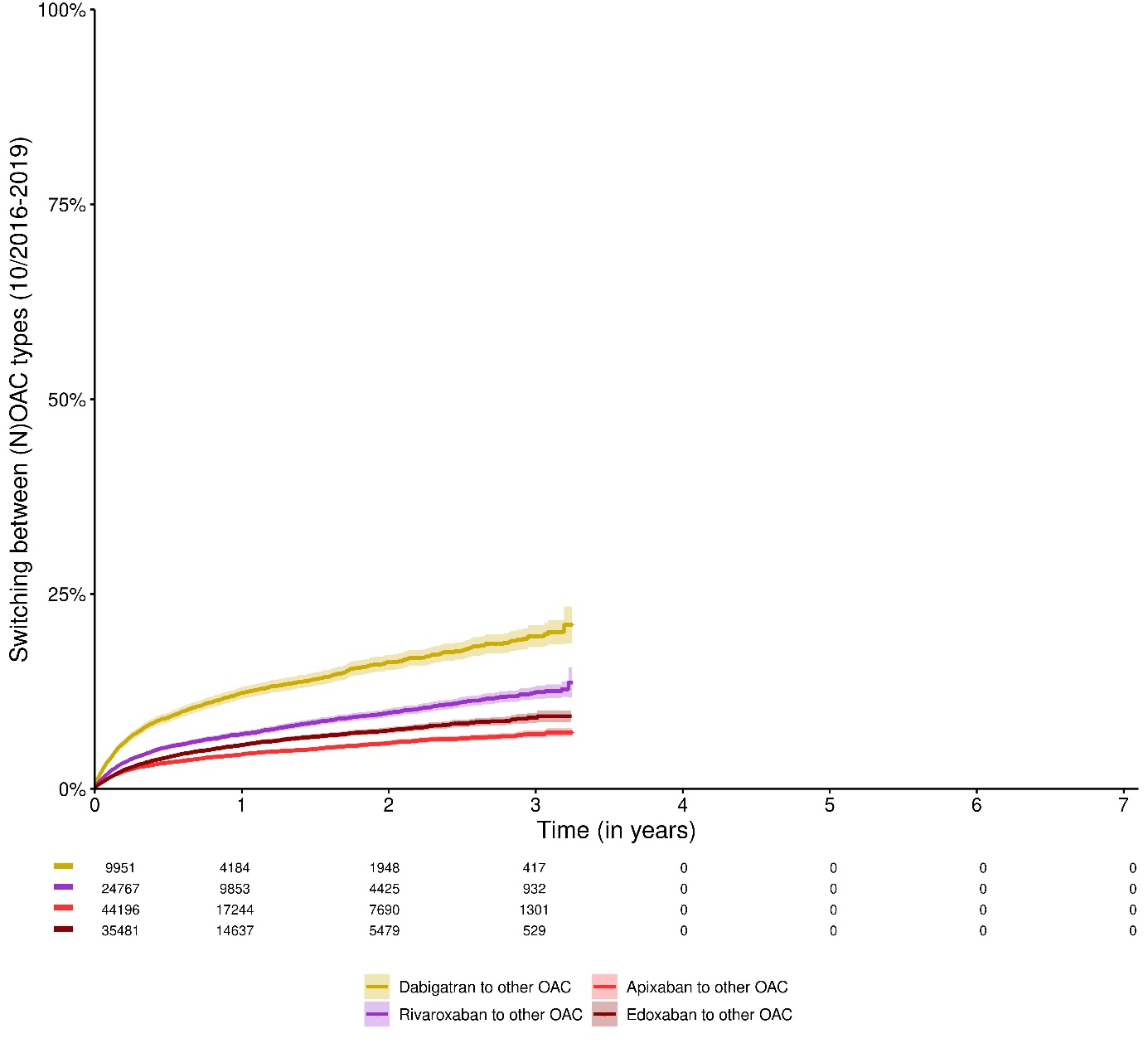


**eFigure 13:** Cumulative incidence curve of switching **A)** NOACs to VKAs or vice versa; and **B)** switching between any type of OAC (e.g. from dabigatran to rivaroxaban) in subjects having initiated treatment between October 1^st^, 2016 and December 31^st^, 2019 (sensitivity analysis).

Data shown as cumulative incidence with 95% confidence interval and risk table (number of patients at risk). In analysis A, subjects were censored in case of discontinuation of NOACs or VKAs (allowing switching between NOAC types and VKA types, respectively), death, emigration or end of the study period; in analysis B, subjects were censored in case of discontinuation of the index OAC type, death, emigration or end of the study period. NOAC: non-vitamin K antagonist oral anticoagulant; OAC: oral anticoagulant; VKA: vitamin K antagonist.

eFigure 14: Adherence (PDC) in subjects having initiated treatment between October 2016 and December 2019 (sensitivity analysis)

**A)**


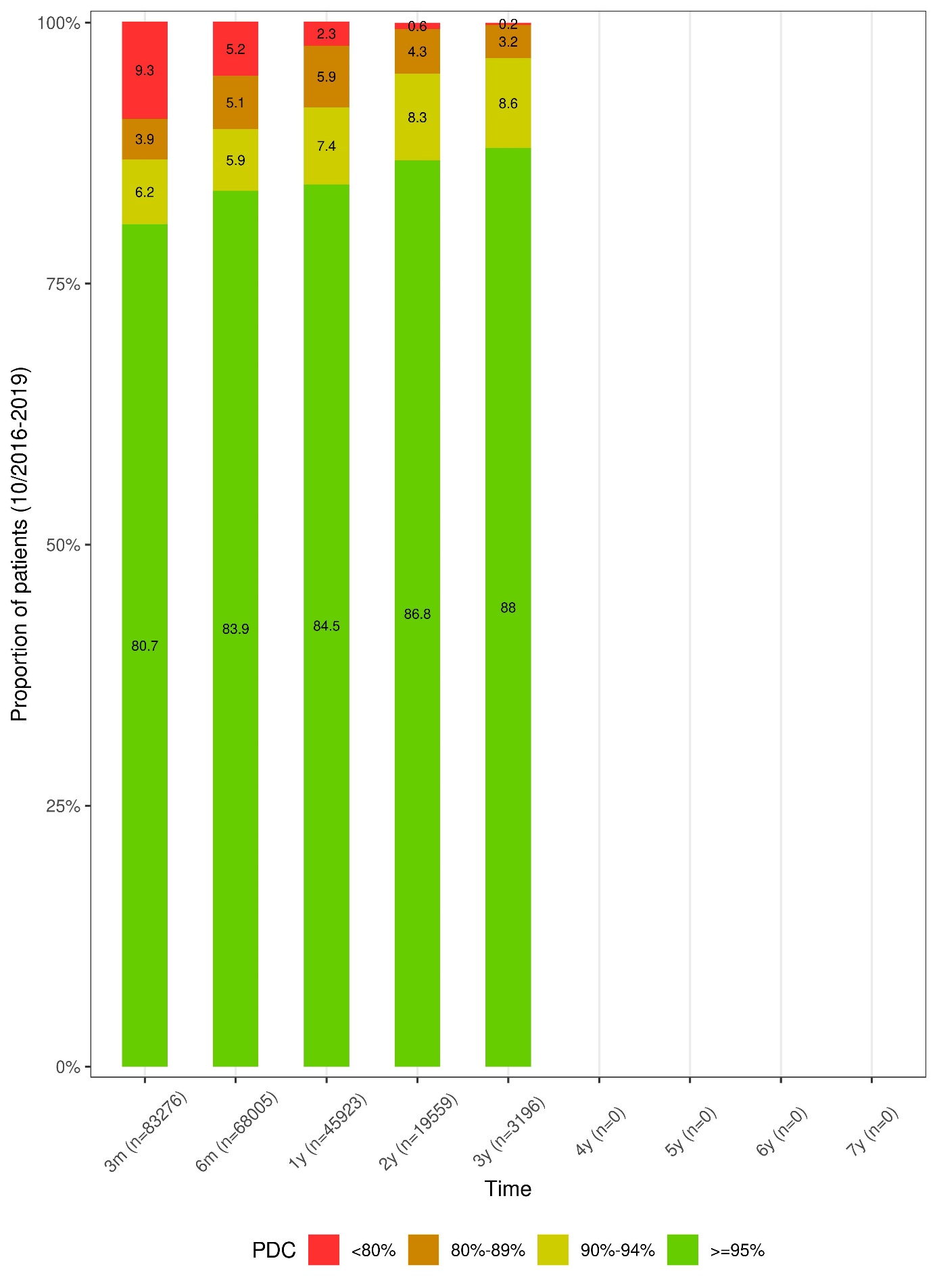


**B)**


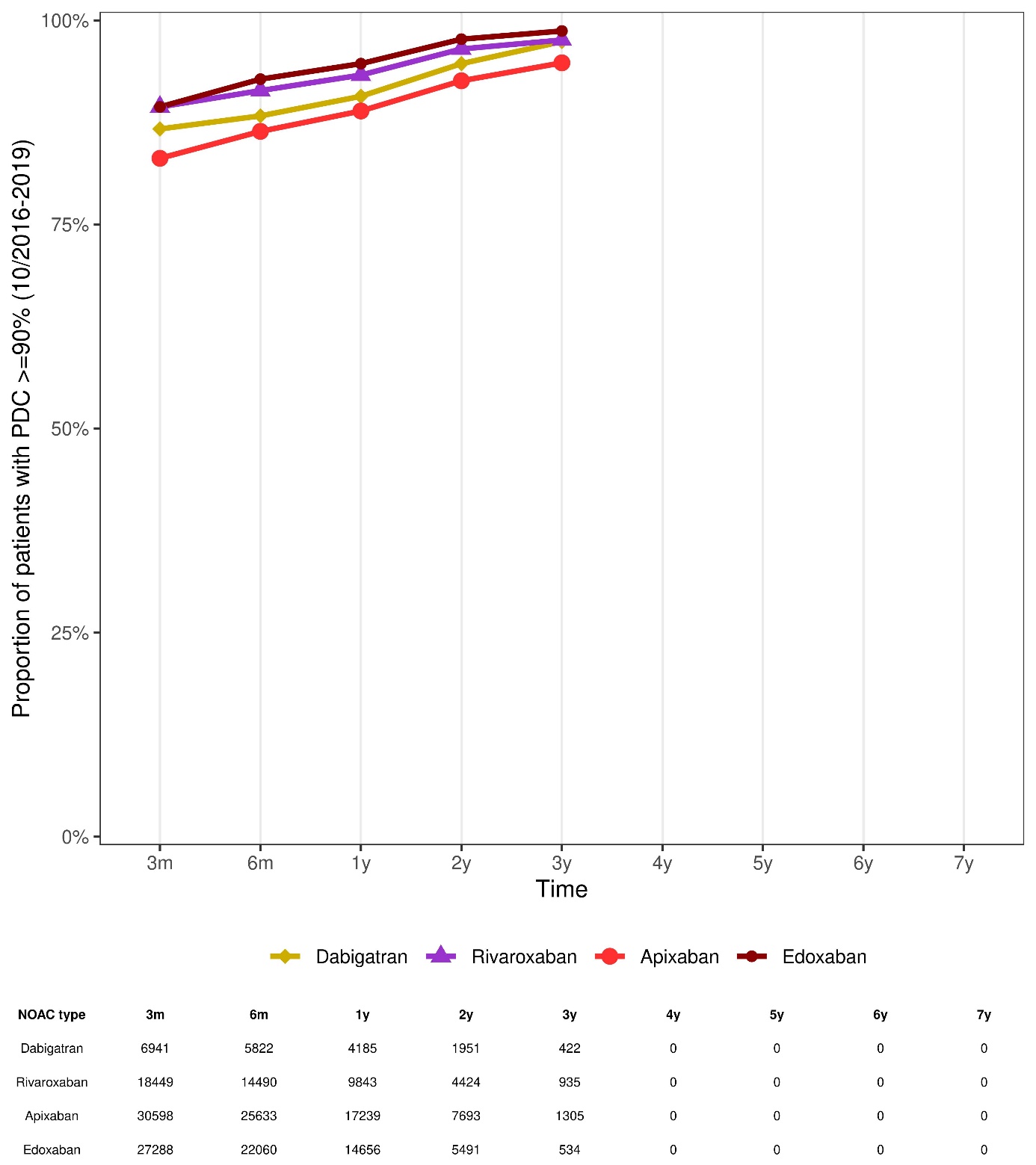


**eFigure 14:** Therapy adherence of **A)** persistent NOAC users, categorized according to a PDC of <80%, 80-<90%, 90-<94% and ≥95%, and **B)** the proportion of persistent dabigatran, rivaroxaban, apixaban and edoxaban users with a PDC of ≥90% at specific time intervals in NOAC-treated subjects having initiated treatment between October 1^st^, 2016 and December 31^st^, 2019 (sensitivity analysis).

Subjects were censored in case of NOAC discontinuation, switching to any other OAC type, death, emigration or end of the study period. Due to their respective approval in September 2013 and October 2016, and the study period ending on December 31^st^, 2019, the maximum follow-up duration of apixaban and edoxaban users was limited to 6.3 years and 3.25 years, respectively. M: month; NOAC: non-vitamin K antagonist oral anticoagulant; OAC: oral anticoagulant; PDC: proportion of days covered; VKA: vitamin K antagonist; y: year.

## eFigure 15: Persistence in subjects with ≥1 year of follow-up (sensitivity analysis)

**A)**


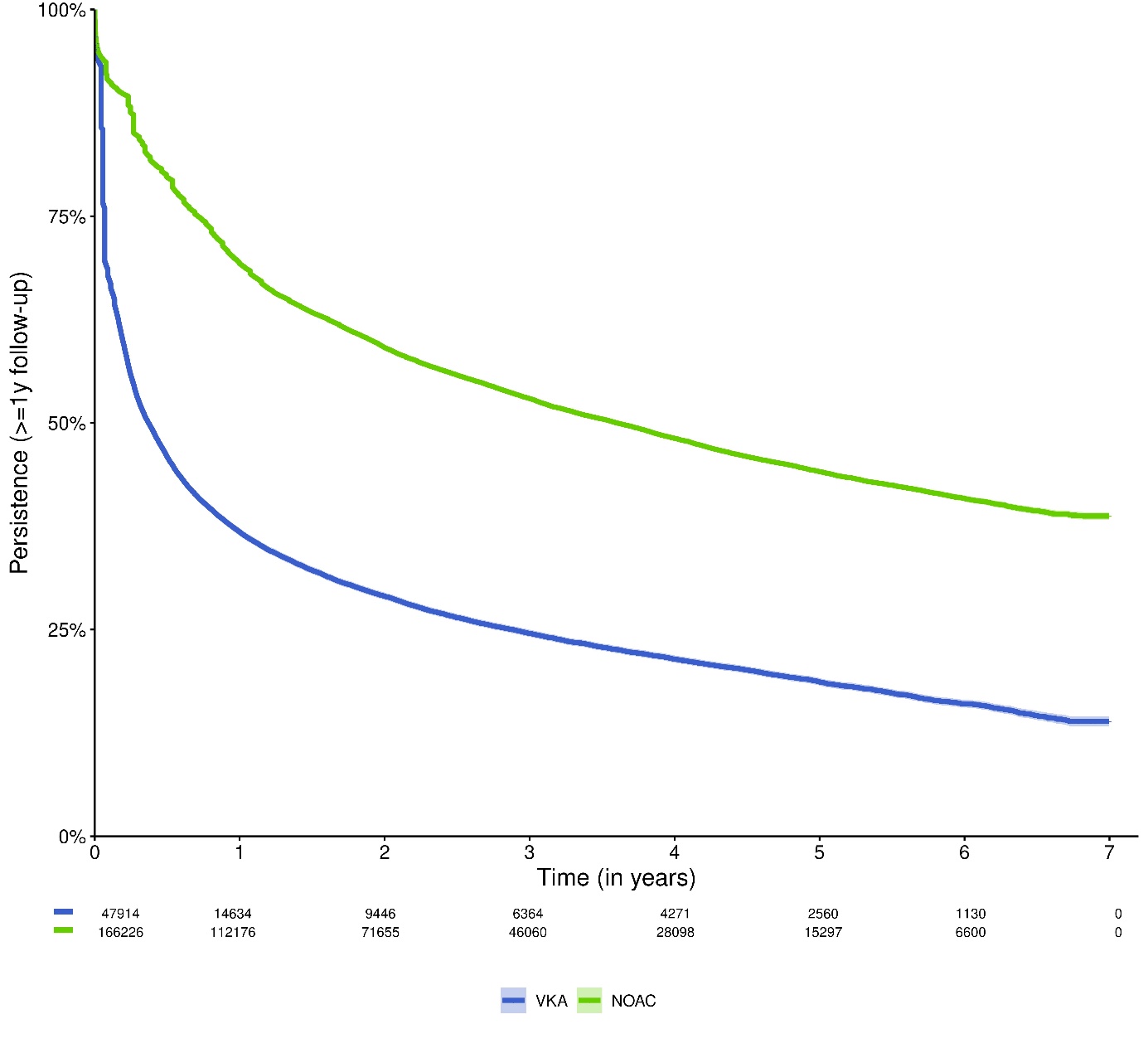


**B)**


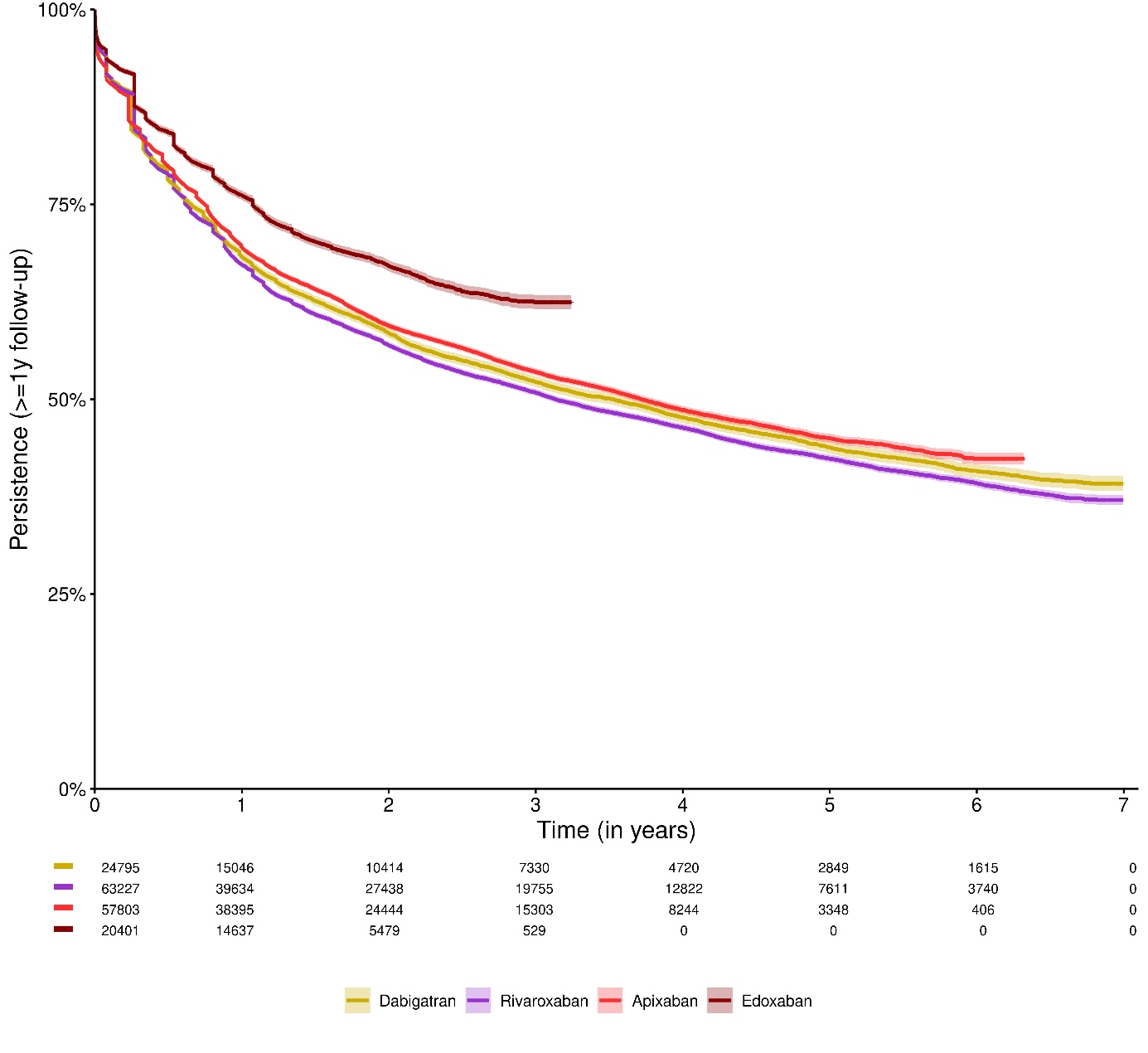


**eFigure 15:** Kaplan-Meier analysis of persistence to **A)** NOACs versus VKAs; and **B)** NOAC types (dabigatran, rivaroxaban, apixaban and edoxaban) in subjects with ≥1 year of follow-up (sensitivity analysis).

Data shown as Kaplan-Meier estimates with 95% confidence interval and risk table (number of patients at risk). In analysis A, subjects were censored in case of switching from NOACs to VKAs or vice versa (allowing switching between NOAC or VKA types), death, emigration or end of the study period; in analysis B, subjects were censored in case of switching to any other OAC type, death, emigration or end of the study period. Due to their respective approval in September 2013 and October 2016, and the study period ending on December 31^st^, 2019, the maximum follow-up duration of apixaban and edoxaban users was limited to 6.3 years and 3.25 years, respectively. NOAC: non-vitamin K antagonist oral anticoagulant; OAC: oral anticoagulant; VKA: vitamin K antagonist; y: year.

eFigure 16: Reinitiation in subjects with ≥1 year of follow-up (sensitivity analysis)

**A)**

**
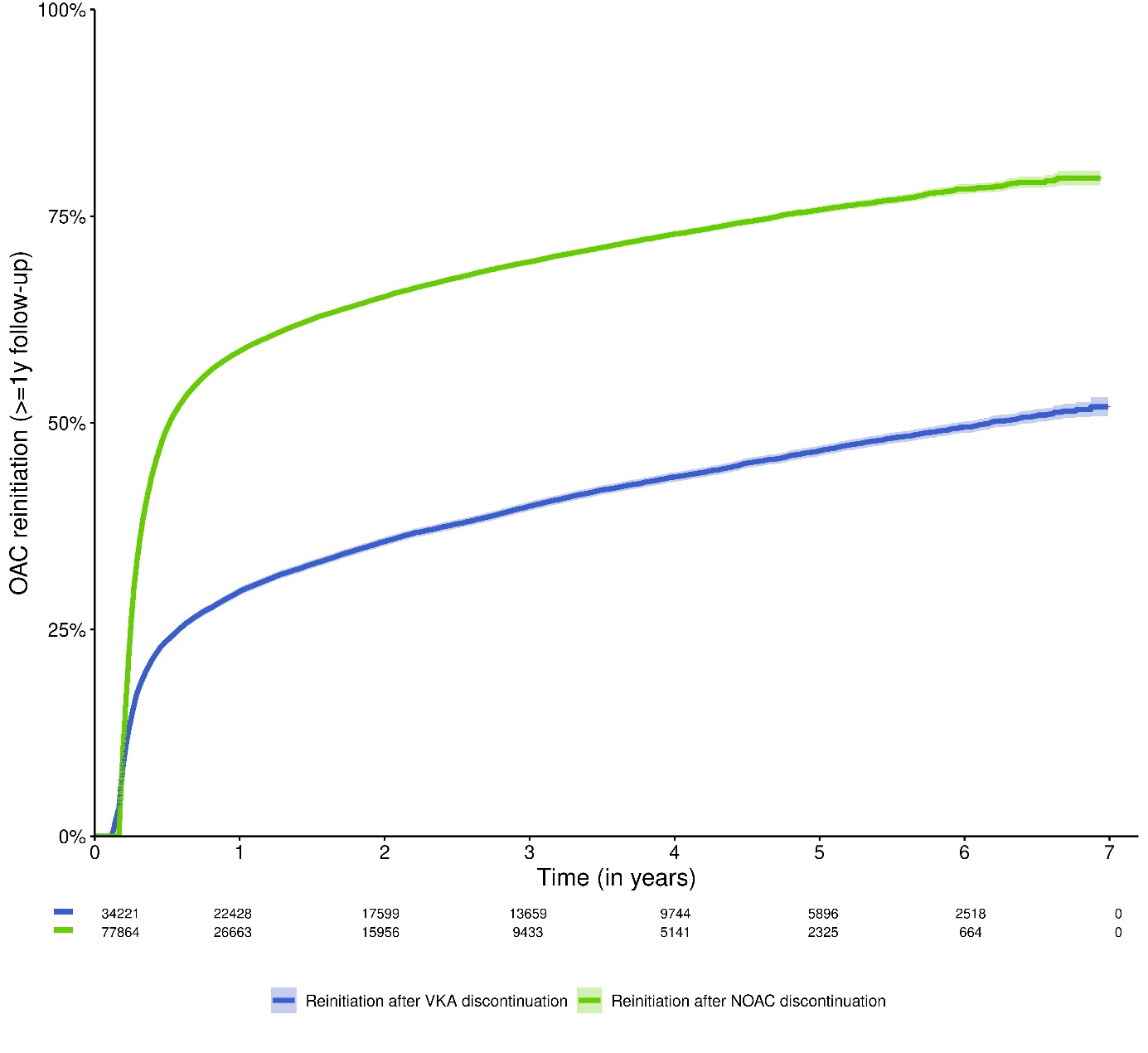
**

**B)**


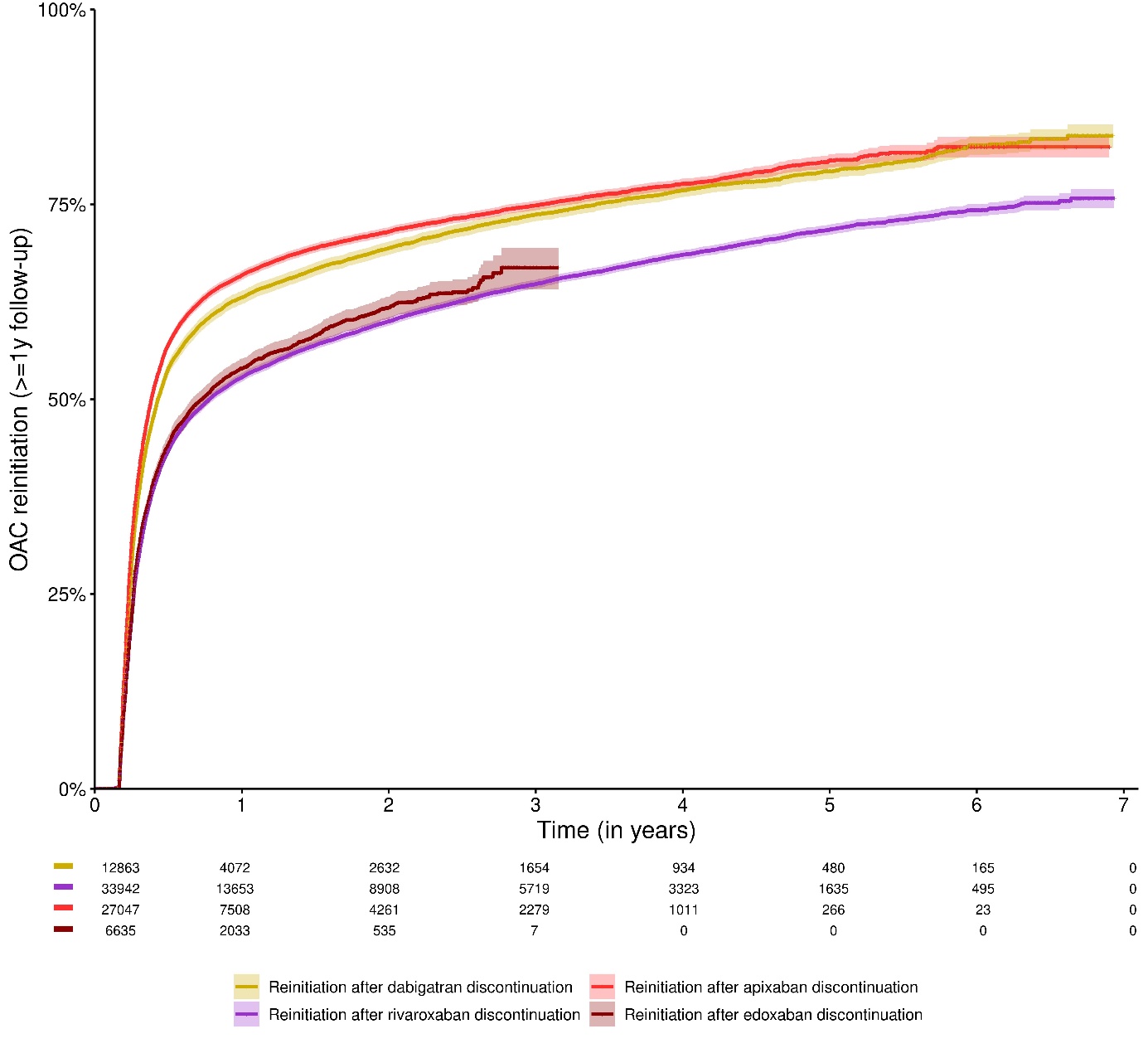


**eFigure 16:** Cumulative incidence curve of reinitiation of an anticoagulant after discontinuation of **A)** NOACs and VKAs; and **B)** NOAC types (dabigatran, rivaroxaban, apixaban and edoxaban) in subjects with ≥1 year of follow-up (sensitivity analysis).

Data shown as cumulative incidence with 95% confidence interval and risk table (number of patients at risk). Subjects were included at the date of discontinuation. Subjects were censored in case of death, emigration or end of the study period. The initial lag period in the cumulative incidence curves (60 days for NOACs, 42-60 days for VKAs) is due to the definition of discontinuation (arbitrary supply gap of >60 days after the calculated last day of supply, with the possibility to extent this gap for VKAs in case of intervening INR testing at least every 42 days). Due to their respective approval in September 2013 and October 2016, and the study period ending on December 31^st^, 2019, the maximum follow-up duration of apixaban and edoxaban users was limited to 6.3 years and 3.25 years, respectively. NOAC: non-vitamin K antagonist oral anticoagulant; OAC: oral anticoagulant; VKA: vitamin K antagonist; y: year.

eFigure 17: Switching in subjects with ≥1 year of follow-up (sensitivity analysis)

**A)**


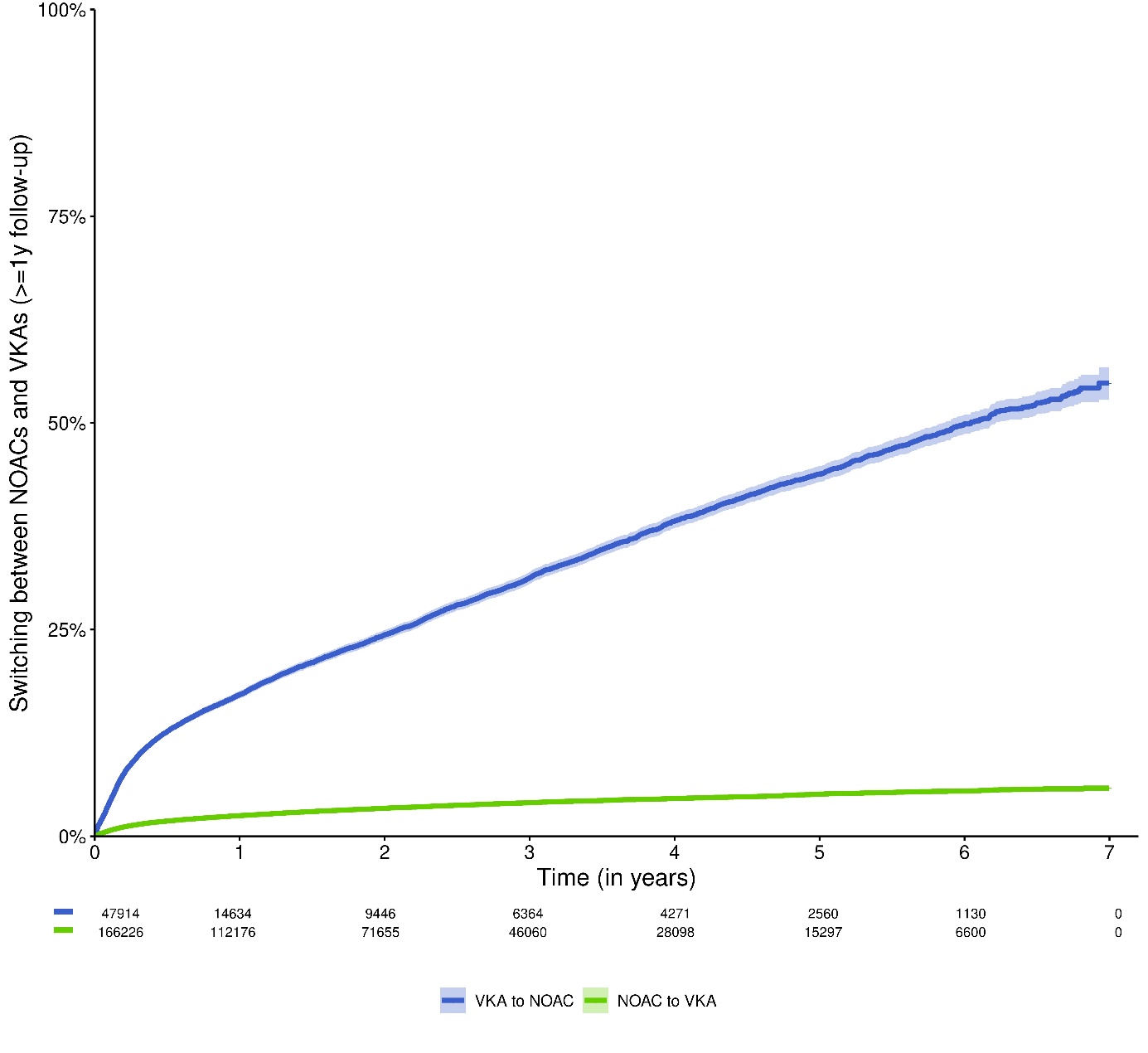


**B)**

**
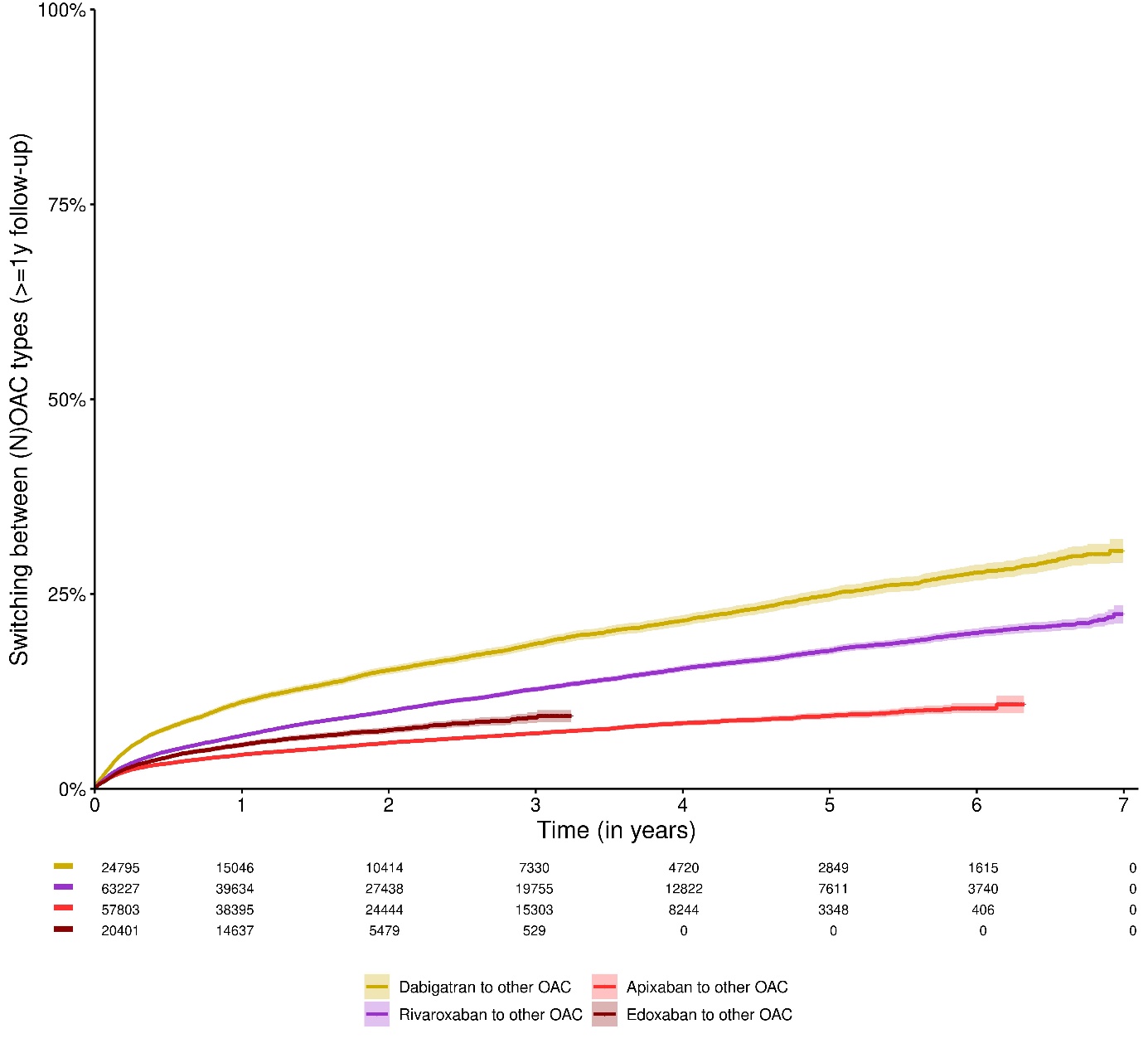
**

**eFigure 17:** Cumulative incidence curve of switching **A)** NOACs to VKAs or vice versa; and **B)** switching between any type of OAC (e.g. from dabigatran to rivaroxaban) in subjects with ≥1 year of follow-up (sensitivity analysis).

Data shown as cumulative incidence with 95% confidence interval and risk table (number of patients at risk). In analysis A, subjects were censored in case of discontinuation of NOACs or VKAs (allowing switching between NOAC types and VKA types, respectively), death, emigration or end of the study period; in analysis B, subjects were censored in case of discontinuation of the index OAC type, death, emigration or end of the study period. Due to their respective approval in September 2013 and October 2016, and the study period ending on December 31^st^, 2019, the maximum follow-up duration of apixaban and edoxaban users was limited to 6.3 years and 3.25 years, respectively. NOAC: non-vitamin K antagonist oral anticoagulant; OAC: oral anticoagulant; VKA: vitamin K antagonist; y: year.

eFigure 18: Adherence (PDC) in subjects with ≥1 year of follow-up (sensitivity analysis)

**A)**
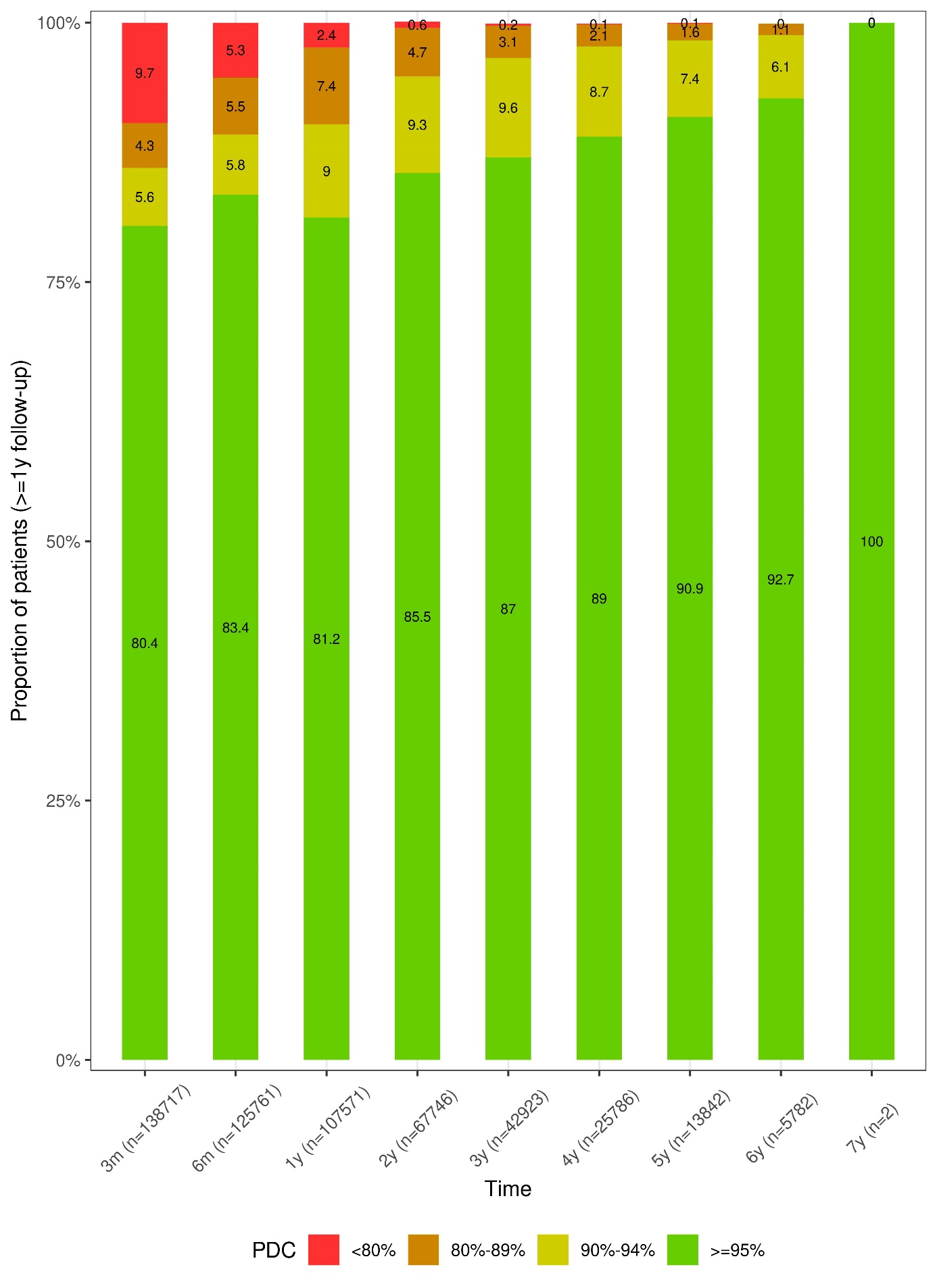


**B)**


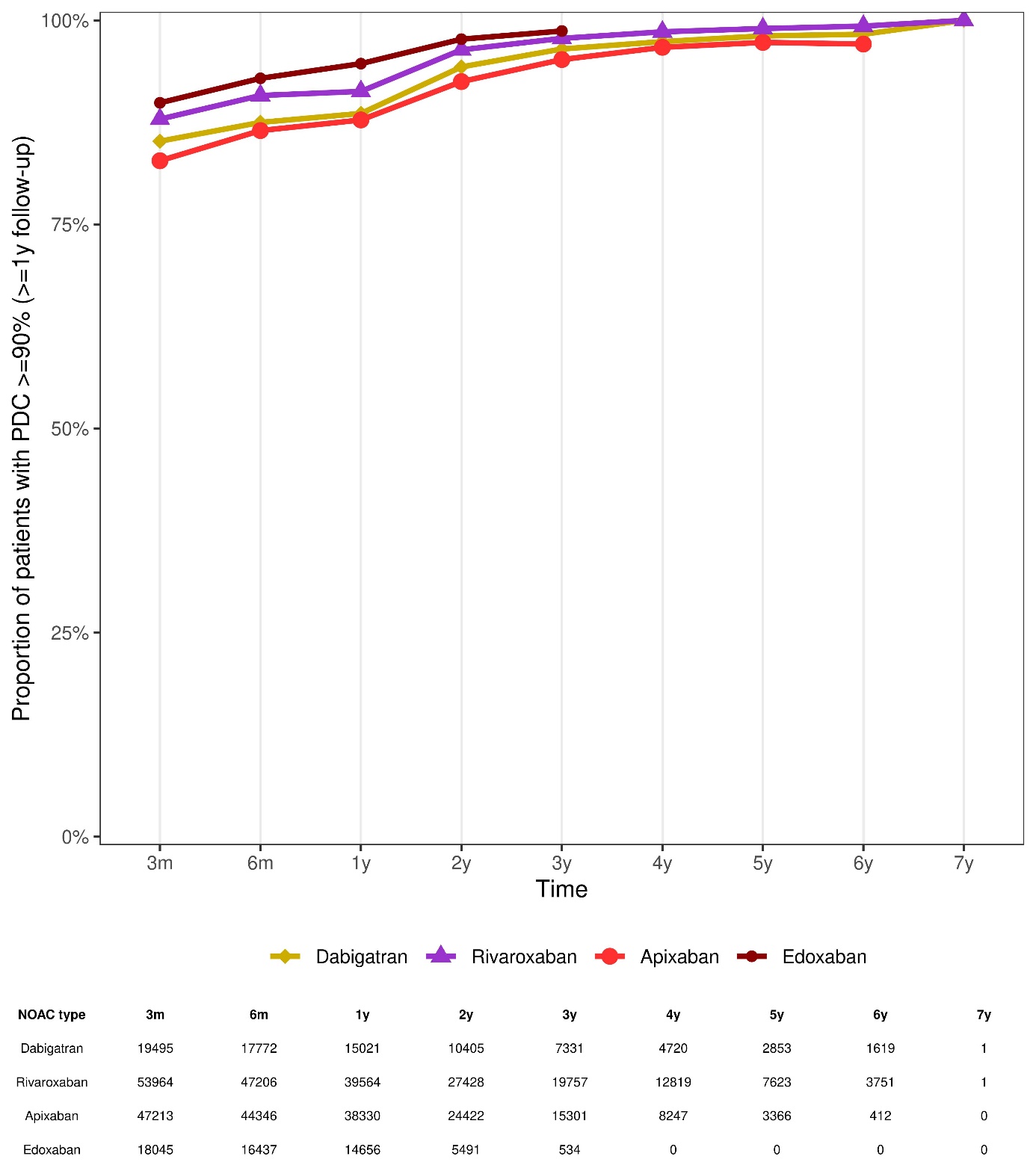


**eFigure 18:** Therapy adherence of **A)** persistent NOAC users, categorized according to a PDC of <80%, 80-<90%, 90-<94% and ≥95%, and **B)** the proportion of persistent dabigatran, rivaroxaban, apixaban and edoxaban users with a PDC of ≥90% at specific time intervals in NOAC-treated subjects with ≥1 year of follow-up (sensitivity analysis).

Subjects were censored in case of NOAC discontinuation, switching to any other OAC type, death, emigration or end of the study period. Due to their respective approval in September 2013 and October 2016, and the study period ending on December 31^st^, 2019, the maximum follow-up duration of apixaban and edoxaban users was limited to 6.3 years and 3.25 years, respectively. Only persistent NOAC users with a follow-up at least corresponding with the examined time interval were investigated (e.g. PDC after 1 year in the subgroup of subjects with ≥1 year of follow-up), as illustrated by the number of investigated subjects (n) per time point. M: month; NOAC: non-vitamin K antagonist oral anticoagulant; OAC: oral anticoagulant; PDC: proportion of days covered; VKA: vitamin K antagonist; y: year.

# References

1. von Elm E, Altman DG, Egger M, Pocock SJ, Gøtzsche PC, Vandenbroucke JP. The Strengthening the Reporting of Observational Studies in Epidemiology (STROBE) statement: guidelines for reporting observational studies. Lancet. 2007;370(9596):1453-7.

2. The International Classification of Diseases (ICD), Clinical Modification. Available from: <https://www.cdc.gov/nchs/icd/index.htm>. Accessed 25 November 2021.

3. RIZIV/INAMI (Rijksinstituut voor ziekte- en invaliditeitsverzekering/Institut national d'assurance maladie-invalidité) medical procedure codes for claims of ambulatory and hospital care. Available from: <https://www.riziv.fgov.be/nl/nomenclatuur/Paginas/default.aspx> (in Dutch/French). Accessed 25 November 2021.

4. WHO Collaborating Centre for Drug Statistics Methodology. Available from: <https://www.whocc.no/>. Accessed 25 November 2021.

5. RIZIV/INAMI (Rijksinstituut voor ziekte- en invaliditeitsverzekering/Institut national d'assurance maladie-invalidité) physician’s speciality codes. Available from: <https://www.riziv.fgov.be/nl/professionals/informatie-algemeen/Paginas/bevoegdheidscodes-riziv-nummer-zorgverleners.aspx> (in Dutch/French). Accessed 25 November 2021.

6. Hindricks G, Potpara T, Dagres N, Arbelo E, Bax JJ, Blomström-Lundqvist C, et al. 2020 ESC Guidelines for the diagnosis and management of atrial fibrillation developed in collaboration with the European Association of Cardio-Thoracic Surgery (EACTS). Eur Heart J. 2021;42(5):373-498.

7. Quan H, Li B, Couris CM, Fushimi K, Graham P, Hider P, et al. Updating and validating the Charlson comorbidity index and score for risk adjustment in hospital discharge abstracts using data from 6 countries. Am J Epidemiol. 2011;173(6):676-82.

8. Charlson ME, Pompei P, Ales KL, MacKenzie CR. A new method of classifying prognostic comorbidity in longitudinal studies: development and validation. J Chronic Dis. 1987;40(5):373-83.

9. Segal JB, Chang HY, Du Y, Walston JD, Carlson MC, Varadhan R. Development of a Claims-based Frailty Indicator Anchored to a Well-established Frailty Phenotype. Med Care. 2017;55(7):716-22.
